# Proton-Coupled Electron Transfer at the Pu^5+/4+^ Couple

**DOI:** 10.1021/jacs.4c06319

**Published:** 2024-07-25

**Authors:** Kaitlyn S. Otte, Julie E. Niklas, Chad M. Studvick, Charlotte L. Montgomery, Alexandria R. C. Bredar, Ivan A. Popov, Henry S. La Pierre

**Affiliations:** † School of Chemistry and Biochemistry, Georgia Institute of Technology, Atlanta, Georgia 30332-0400, United States; ‡ Department of Chemistry, University of Akron, Akron, Ohio 44325-3601, United States; § Department of Chemistry, University of North Carolina at Chapel Hill, Chapel Hill, North Carolina 27599-3290, United States; ∥ Department of Chemistry, Washington State University, Pullman, Washington 99164, United States; ⊥ Nuclear and Radiological Engineering and Medical Physics Program, School of Mechanical Engineering, Georgia Institute of Technology, Atlanta, Georgia 30332-0400, United States; # Physical Sciences Division, Pacific Northwest National Laboratory, Richland, Washington 99352, United States

## Abstract

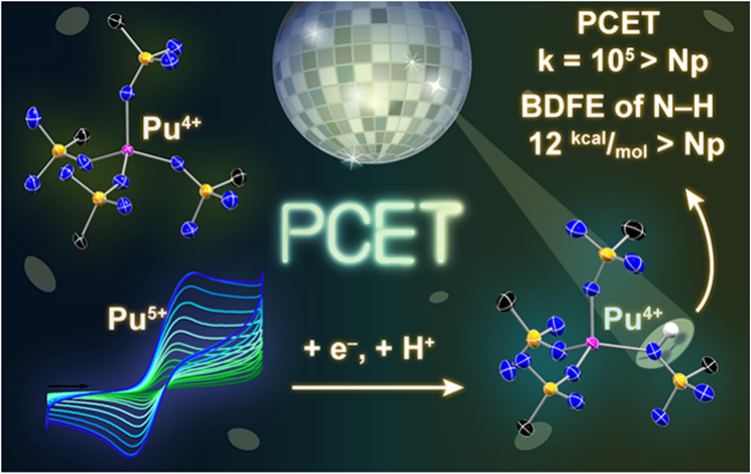

The synthesis and solution and solid-state characterization of [Pu^4+^(NPC)_4_], **1-Pu**, (NPC = [NP^*t*^Bu(pyrr)_2_]^−^; ^*t*^Bu = C(CH_3_)_3_; pyrr = pyrrolidinyl) and [Pu^3+^(NPC)_4_][K(2.2.2.-cryptand)], **2-Pu**, is described. Cyclic voltammetry studies of **1-Pu** reveal a quasi-reversible Pu^4+/3+^ couple, an irreversible Pu^5+/4+^ couple, and a third couple evincing a rapid proton-coupled electron transfer (PCET) reaction occurring after the electrochemical formation of Pu^5+^. The chemical identity of the product of the PCET reaction was confirmed by independent chemical synthesis to be [Pu^4+^(NPC)_3_(HNPC)][B(ArF_5_)_4_], **3-Pu**, (B(ArF_5_)_4_ = tetrakis(2,3,4,5,6-pentafluourophenyl)borate) via two mechanistically distinct transformations of **1-Pu**: protonation and oxidation. The kinetics and thermodynamics of this PCET reaction are determined via electrochemical analysis, simulation, and density functional theory. The computational studies demonstrate a direct correlation between the changing nature of 5*f* and 6*d* orbital participation in metal–ligand bonding and the electron density on the N_im_ atom with the thermodynamics of the PCET reaction from Np to Pu, and an indirect correlation with the roughly 5-orders of magnitude faster Pu PCET compared to Np for the An^5+^ species.

## Introduction

The delineation of periodic trends is critical to mapping the unique bonding^1−12^ and reactivity^13−21^ of the actinides. The recent renewed interest in plutonium chemistry has greatly expanded the number of well-characterized complexes.^13,20,22−28^ However, comparative reactivity studies across the mid-actinides are rare.^13,26,29−36^ Recently, we reported the redox properties of a valence series of four-coordinate neptunium complexes supported by the NPC ligand (NPC = [NP^*t*^Bu(pyrr)_2_]^−^; ^*t*^Bu = C(CH_3_)_3_; pyrr = pyrrolidinyl).^37,38^ These studies established the first Np^5+/4+^ couple in a complex without a metal–ligand multiple bond (MLMB),^37^ the isolation of a 4-coordinate Np^5+^ complex, and the characterization of the proton-coupled electron transfer (PCET) reactivity of this Np^5+^ complex in tetrahydrofuran (THF).^38^ PCET chemistry has been invoked as a critical mechanism in the redox chemistry of the actinyls,^15,16,39−51^ but the thermodynamic and kinetic parameters of this fundamental transformation have not been measured. The lack of mechanistic analysis of these reactions is due in part to chemical issues, as the fundamental step in aqueous transformations of actinyl complexes is often masked by subsequent transformations including disproportionation, hydrolysis, and oligomerization. These measurements are further complicated by the significant radiological constraints in handling transuranic materials.

Herein, we present the isolation, characterization, and redox chemistry of a tetrahomoleptic complex, [Pu^4+^(NPC)_4_] (**1-Pu**), and its chemical reduction to [Pu^3+^(NPC)_4_][K(2.2.2.-cryptand)] (**2-Pu**). Cyclic voltammetry studies of **1-Pu** indicate an electrochemical-chemical (EC) transformation at the Pu^5+/4+^ couple, and the chemical identity of the reaction product was established by independent synthesis via two methods. Oxidation of **1-Pu** with [Cp_2_Fe][B(ArF_5_)_4_] (B(ArF_5_)_4_ = tetrakis(2,3,4,5,6-pentafluourophenyl)borate) in THF or protonation of **1-Pu** with [H(OEt_2_)_2_][B(ArF_5_)_4_] yields [Pu^4+^(NPC)_3_(HNPC)][B(ArF_5_)_4_] (**3-Pu**) and implicate a rapid PCET reaction proceeding in THF after either chemical or electrochemical oxidation. Electrochemical analysis and simulation, paired with density functional theory (DFT), are used to define the thermodynamic and kinetic parameters of this transformation. Critically, this data facilitates the comparative analysis of PCET at an actinide An^5+/4+^ (An = Np or Pu) couple within a conserved ligand sphere. Changes in the nature of metal–ligand covalency in Pu^4+^ and Pu^5+^ (versus Np^4+^ and Np^5+^) drive the significant differences in the thermodynamics of this PCET reaction.

## Results and Discussion

### Synthesis and Solution and Solid-State Characterization

**Figure 1:**
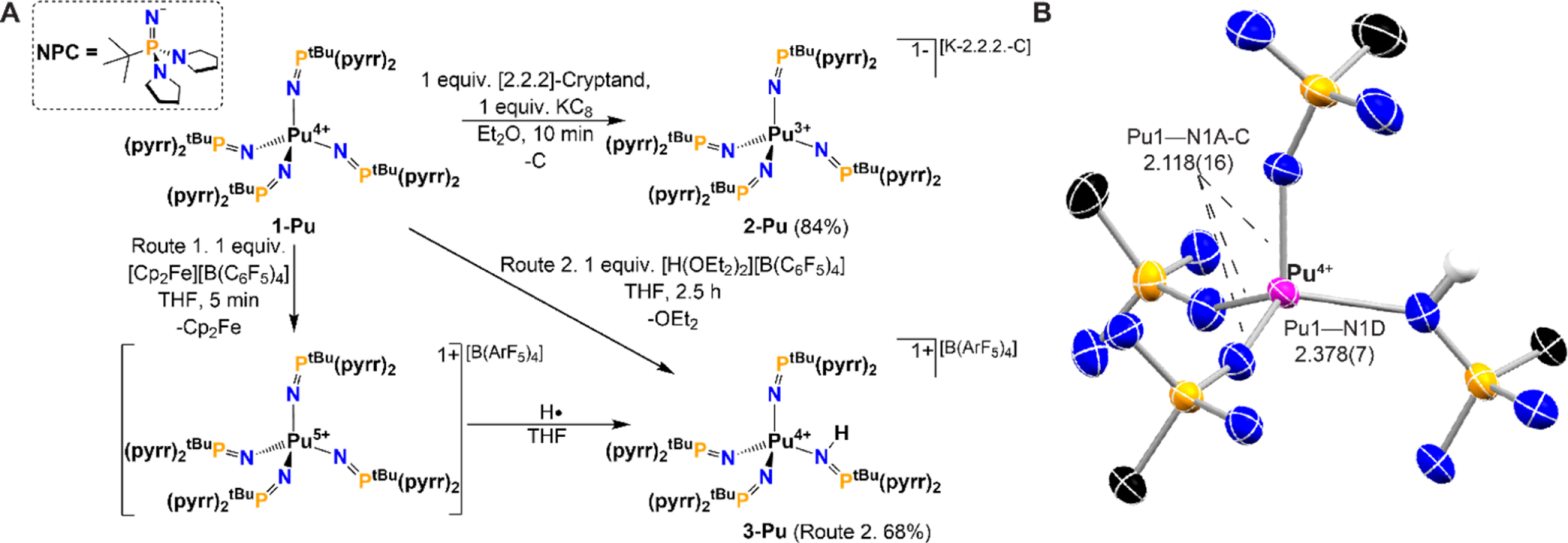
(A) Reduction of complex **1-Pu** to **2-Pu**, and the synthesis of **3-Pu** by (Route 1) oxidation of **1-Pu** and subsequent PCET reaction or (Route 2) direct protonation of **1-Pu**. (B) Molecular structure of **3-Pu** as determined by single-crystal X-ray diffraction (SC-XRD). Thermal ellipsoids are shown at 50% (atom colors: Pu, pink; N, blue; P, orange; C, black; H, white). [B(ArF_5_)_4_] anion, H atoms (except the HNPC ligand), and all except quaternary C atoms of the cation omitted for clarity. Only one component of the disordered molecule is shown for clarity.

The neutral, homoleptic, tetravalent Pu complex, **1-Pu**, supported by the NPC ligand was prepared in high yield (91%, see Supporting Information (SI) for details) from [PuCl_4_(DME)_2_] (DME = 1,2-dimethoxyethane).^22^**1-Pu** is isostructural with the previously reported Ce, U, and Np analogs.^37,52^ Bond metrics across the four complexes are comparable (Table S8 for comparison of metrics). **1-Pu** was reduced with KC_8_ to produce the trivalent **2-Pu** in 84% yield. The complex is a salt supported by an outer sphere potassium-2.2.2-cryptand cation, as shown in Figure 1. **2-Pu** is also isostructural with the trivalent Ce and Np analogues despite it crystallizing in a higher symmetry space group (*Pbca* vs. *P*2_1_/*c*). A τ_4_ value was calculated for both complexes as a measure of the structural distortion from a perfectly tetrahedral geometry using the inner N atom coordination sphere, defined as (360° – (α + β))/141° (where α and β are the largest angles in the coordination tetrahedron).^53^ Both **1-Pu** and **2-Pu** have τ_4_ values showing they are nearly tetrahedral in the inner coordination sphere (Table 1). For these NPC complexes, the average An–N distance contracts as expected with ionic radius^54^ across the actinide series, from 2.167(7) Å for U to 2.141(11) Å for **1-Pu** in the 4+ oxidation state, and from 2.291(5) Å for Np to 2.257(10) Å for **2-Pu** in the 3+ oxidation state.^37,52^

**Table 1:** Selected Bond Metrics^a^

	**1-Pu**	**2-Pu**	**3-Pu**
M–N (Å)	2.141(11)^b^	2.257(10)^b^	2.118(16)
			2.378(7)^c^
P=N (Å)	1.540(13)	1.537(15)	1.557(4)
			1.583(7)^c^
τ_4_	0.97(2)	0.99(4)	0.92(2)
N–M–N (deg)	109(5)	109(1)	109(5)
M–N=P (deg)	157(6)	147(8)	156(7)
			139.7(3)^c^

a Values are averages, and numbers given in parentheses are standard deviations.

b Denotes that an average esd value is given, as the average lengths are from 4–20 independent measurements (due to symmetry, *Z*, and disorder) which have a wide distribution of M–N distances.

c Shows the metric pertaining to the one unique protonated ligand.

The solution electronic absorption (Figures S4–S7, S10–S11) and nuclear magnetic resonance (NMR) (multinuclear and two-dimensional (2D) techniques, Figures S13–S17) spectroscopic analyses of **1-Pu** and **2-Pu** are fully congruent with the solid-state structural assignments. The resonances observed in the ^1^H NMR spectra of **1-Pu** occupy a narrow window from 1 to 3.5 ppm, falling within the 0 to 4 ppm range seen for Ce, U, and Np. There is, however, a large shift in the resonance of the ^31^P{^1^H} NMR (139.8 ppm for Pu vs. 299.9 ppm of Np, 332.7 ppm U, and 1.6 ppm of Ce), reflecting the sensitivity of phosphorus NMR to the number of unpaired *f* electrons for these tetravalent ions: Ce, 4*f*^0^; U, 5*f*^2^; Np, 5*f*^3^; Pu, 5*f*^4^. The trivalent state is confirmed for **2-Pu** by the presence of identifying *f*–*f* transitions near 580, 620, 900, and 1430 nm with extinction coefficients of 10–80 M^–1^ cm^–1^.^20,55^ Likewise, two diagnostic *f*–*f* transitions centered near 1100 nm affirm the tetravalent assignment of **1-Pu**.^55^

### Electrochemical Analysis

**Figure 2:**
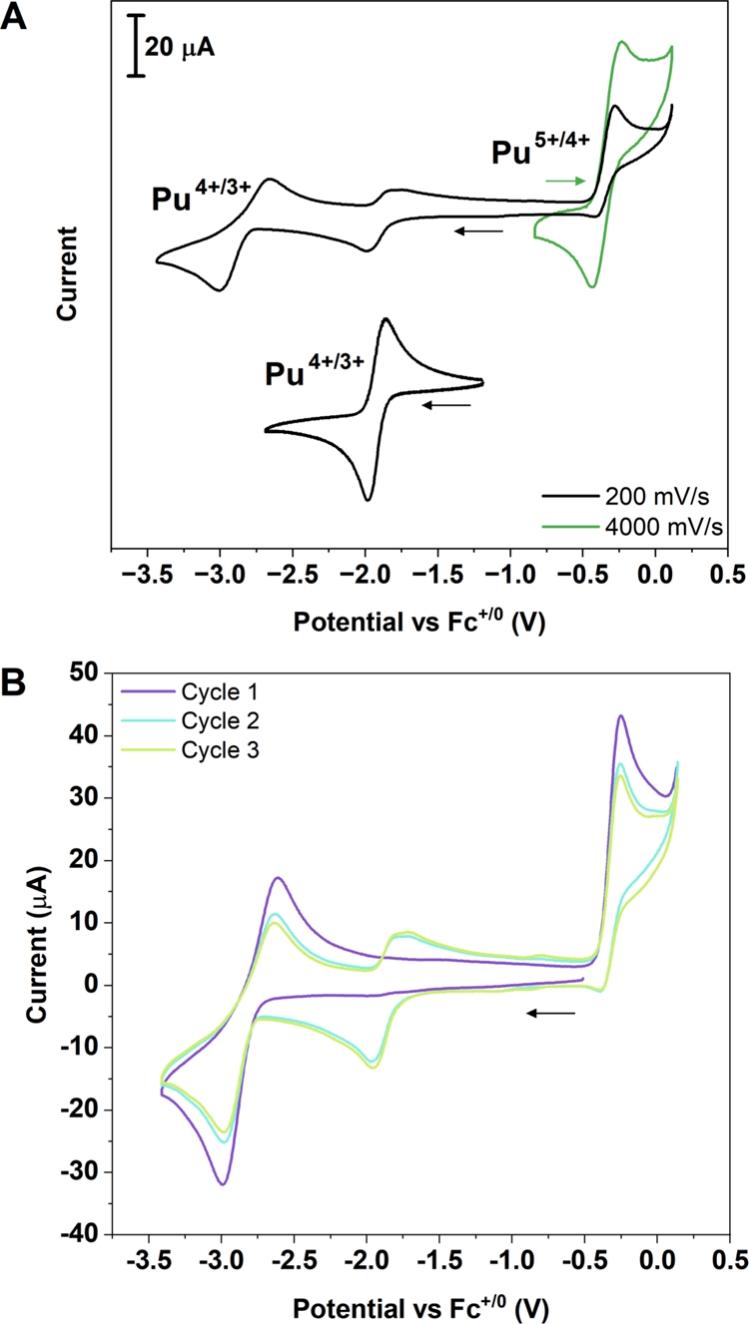
(A) Cyclic voltammograms (CVs) of 2.5 mM **1-Pu** (top) and 2.5 mM 3-Pu (bottom). (B) Voltammogram cycles 1–3 of **1-Pu** at 200 mV/s. All CVs in this work were recorded under a N_2_ atmosphere in THF with 0.05 M [N(*^n^*Bu)_4_][BPh_4_] as a supporting electrolyte (WE: glassy carbon; RE: polished Ag wire pseudoreference, fritted; CE: Pt wire). CVs are plotted in IUPAC convention and individually referenced to an internal Fc^+/0^ couple, with arrows indicating direction and starting point of scan. Applied voltage was corrected for Ohmic drop using positive feedback *R*_u_. Additional details in SI.

**Figure 3:**
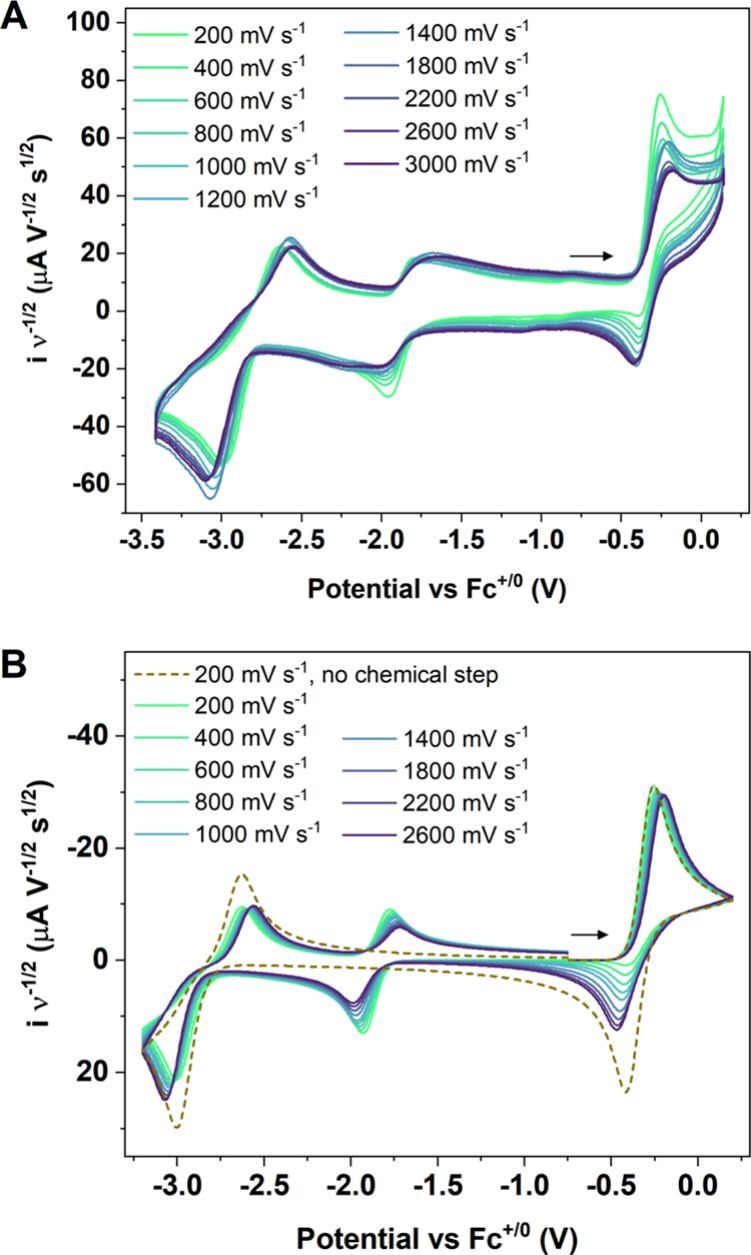
(A) CVs of 2.3 mM **1-Pu** in THF with 0.05 M [N(*^n^*Bu)_4_][BPh_4_] as a supporting electrolyte at scan rates ranging from 200 to 3000 mV/s. Current values have been normalized to scan rate. (B) Simulated CVs of 2.3 mM **1-Pu** using simulation parameters in Tables S3, S5, and S6.

A cyclic voltammogram (CV) of 2.3 mM **1-Pu** in THF with 0.05 M [N(*^n^*Bu)_4_][BPh_4_] as supporting electrolyte recorded at 200 mV/s scan rate reveals a quasi-reversible wave with an *E*_1/2_ = −2.83 V, assigned to the Pu^4+/3+^ wave, an irreversible oxidation with an *E*_pa_ = −0.20 V, assigned to the Pu^5+/4+^ wave, and a quasi-reversible feature with an *E*_1/2_ = −1.92 V, which will be assigned below (all values vs. Fc^+/0^, Fc = Cp_2_Fe; Figures 2–3).

To further understand the relationship between these redox couples, cycling experiments were performed (Figure 2B). During the first cycle, starting at the resting potential and scanning cathodically at 200 mV/s, the quasi-reversible peak Pu^4+/3+^ couple (*E*_1/2_ = −2.83 V, Figure 2B) was observed, and, on the anodic segment, the irreversible Pu^5+/4+^ oxidation (*E*_pa_ = −0.20 V) was observed (Figure 2B). When multiple cyclic voltammograms are collected, the subsequent cycles show an additional quasi-reversible feature with an *E*_1/2_ = −1.92 V, which only appears after the irreversible oxidation is completed (Figure 2B).

The irreversible wave of the Pu^5+/4+^ couple is indicative of an EC mechanism, where electrochemical oxidation from Pu^4+^ to Pu^5+^ is followed by a chemical step, which we establish (see below) is H atom abstraction to form **3-Pu**. Given this reactivity, the quasi-reversible feature at −1.92 V can be assigned to the Pu^4+/3+^ couple of **3-Pu**. Synthetic isolation of **3-Pu**, detailed below, supports this hypothesis since CVs of **3-Pu** show a reversible wave (*E*_1/2_ = −1.92 V) that overlays with the quasi-reversible feature present in the CV for **1-Pu** (*E*_1/2_ = −1.92 V, Figure 2A). This electrochemical behavior of **3-Pu** confirms the assignment of the third wave as the Pu^4+/3+^ wave of **3-Pu** that was formed *in situ* during the electrochemical experiment. The electrochemical conversion of **1-Pu** to **3-Pu** is not quantitative, as can be observed from the two anodic features present (Figure 2B), compared to the independently prepared **3-Pu**, which presents a single, clear reversible couple.

Importantly, scan-rate dependence studies of **1-Pu** showed that reversibility of the Pu^5+/4+^ wave can be enhanced at faster scan rates, indicating that electrochemical reduction can outcompete the chemical reactivity of Pu^5+^ at high scan rates (Figures 3 and S25). However, in the range of scan rates studied, full reversibility of the wave is not achieved, and the reduction of Pu^5+^ to **1-Pu** at the fastest rates studied was not quantitative. A redox feature at −1.92 V is observed for all scan rates investigated, indicating some chemical reactivity is retained at the Pu^5+/4+^ couple at even the fastest scan rates. Based on the established PCET reactivity of [Np^5+^(NPC)_4_][B(ArF_5_)_4_] in THF,^38^ the protonated form of **1-Pu**, namely **3-Pu**, is established as the net product of electrochemical oxidation and subsequent H atom abstraction.

### Kinetic Analysis

Analysis of the scan rate dependent voltammograms of **1-Pu** was conducted to obtain a rate constant for the chemical step in the proposed EC reactivity (electrochemical oxidation and subsequent H atom abstraction). Enhanced reversibility at faster scan rates for the Pu^5+/4+^ wave (Figure 3A) allowed for analysis of the data to obtain a rate constant for the consumption of the putative Pu^5+^ → **3-Pu**. This reaction was modeled as the decomposition of the Pu^5+^ species via an EC mechanism^56^ in the presence of a high excess of THF as the H atom source (akin to the conditions in NMR experiments on the Np^5+^ analogue). The ratio of the anodic and cathodic peak currents of the Pu^5+/4+^ wave was used to measure the rate constant (Figure S31), which is related to the kinetic competition between the experiment time (scan rate or switching potential) and the rate constant of the reaction. At slower scan rates, or at potentials well beyond the initiating redox event, there is more time for the reaction to occur and the returning peak current will be smaller.

**Figure 4:**
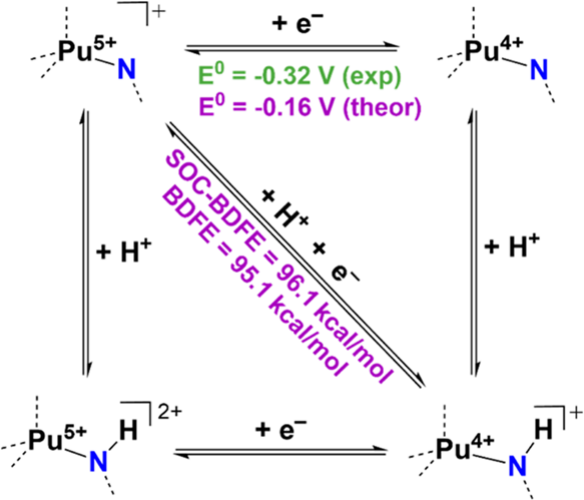
Square scheme for PCET mechanism with computed (violet) and experimental (green) values.

A working curve^56^ was used to plot the peak current ratios vs. log(*k*_f_τ), where τ is the experiment time and *k*_f_ is the rate constant of the reaction. This working curve can be fit to a sigmoid function that was used to solve for *k*_f_ (Figure S30). The rate constant, *k*_f_, was measured to be 1.7 ± 0.4 s^–1^. Electrochemical simulations were used to probe the voltammetric response using our hypothesized mechanism and experimentally determined rate constant. To properly simulate the data, diffusion coefficients and heterogeneous rate constants were measured using Randles–Sevcik analysis and trumpet plot analysis, respectively, for **1-Pu** and **3-Pu** (Figures S32–S37). Full simulation parameters can be found in the SI (Tables S1–S6). The proposed mechanism (oxidation for Pu^4+^ → Pu^5+^, followed by a PCET reaction of Pu^5+^ to form **3-Pu** as shown in Figure 4) gives a general CV shape that agrees with measured CVs.

In summary, the simulated cyclic voltammograms using the proposed EC mechanism with *k*_f_ = 1.7 s^–1^ agree very well with experimentally observed cyclic voltammograms (Figures 3B, S38). Simulations were also performed with rate constants an order of magnitude greater (30 s^–1^) (Figure S39) and smaller (0.03 s^–1^) (Figure S40) than the experimentally determined value. The lack of agreement between these simulations and experimental voltammograms, based on the differences in peak reversibility, further supports that *k*_f_ = 1.7 s^–1^ describes the rate of the chemical reaction well.

The determination of the rate constant for this reaction presents the opportunity for the first quantitative comparison of kinetic parameters for a PCET reaction of transuranic ions. The [U^5+^(NPC)_4_][B(ArF_5_)_4_] is stable in THF over the course of days (as observed by NMR).^37^ [Np^4+^(NPC)_4_][B(ArF_5_)_4_] is stable in the solid-state and undergoes a relatively slow PCET reaction in THF (*k*_f_ = 8 × 10^–5^ s^–1^, determined by NMR.^38^ The PCET reaction studied here occurs roughly 5-orders of magnitude faster than with Np. These results demonstrate that a PCET reaction can provide a mechanism for kinetic separation across these mid-actinides.

### Synthetic Identification of the PCET Product

As mentioned above, to demonstrate that the third couple in the CV of **1-Pu** is attributable to the *in situ* production of **3-Pu**, **3-Pu** was independently prepared via two mechanistically distinct reactions. Protonation of **1-Pu** with [H(OEt_2_)_2_][B(ArF_5_)_4_],^57^ yields **3-Pu** (Figure 1A) and facilitates characterization of this material along with its electrochemical analysis. In congruence with the hypothesis that **3-Pu** is generated via a PCET reaction after the chemical oxidation of **1-Pu**, chemical oxidation with [Cp_2_Fe][B(ArF_5_)_4_] in THF also yields **3-Pu** (Figure 1A). Fc^+^ is expected to be a competent oxidant based on the observed potential for the Pu^5+/4+^ couple of −0.32 V vs. Fc^+/0^.

The identity of the independently prepared **3-Pu** was established by SC-XRD and solution NMR studies. Single crystals of **3-Pu** were obtained from the oxidation reaction and SC-XRD analysis revealed that the molecular structure (Figure 1B) of this material is isomorphic with the previously reported Np complex crystallizing in *P*1̅.^38^ The unique hydrogen atom was assigned from the nearby residual electron density peak and refined well as a proton localized on N1D. A nominal decrease in M–N distance between the NPC ligands in **1-Pu** and **3-Pu** and a substantial (∼0.23 Å) lengthening in the M–N bond to the HNPC ligand (Tables 1 and S8) is found. The τ_4_ value additionally drops for **3-Pu** due to the presence of the proton altering the inner coordination sphere. All observations are consistent with the relative changes in bond metrics observed for [Np^4+^(NPC)_3_(HNPC)][B(ArF_5_)_4_] and [Np^4+^(NPC)_4_] (Table S8). This comparison illustrates the small contraction in the bond lengths, consistent with the change in effective ionic radii,^54^ between the Np and Pu analogues.

The solution NMR of **3-Pu** (heteronuclear and 2D, Figures S18–S23) agrees with the SC-XRD assignment. Observed ^1^H resonances span the chemical shift region, 0.5–5.5 ppm, as for **1-Pu**. The unique HNPC proton on **3-Pu** is observed as a very broad singlet at 3.15 ppm in the ^1^H spectrum. The ^31^P{^1^H} spectrum shows two signals in a 3:1 ratio of intensities at 78.9 (NPC) and 45.6 ppm (HNPC). Each signal experiences less paramagnetic shift from the free ligand than **1-Pu**, with the protonated ligand in **3-Pu** only slightly shifted from the ^31^P{^1^H} resonance of HNPC (47.5 ppm).^52^ The electronic absorption spectrum of **3-Pu** is similar to that of **1-Pu** and provides further confirmation of the tetravalent oxidation state of **3-Pu**. The diagnostic pair of peaks near 1100 nm in the electronic absorption spectra is observed (Figure S8).

### Computational Analysis

DFT calculations were employed to understand the origin of the PCET reactivity arising from the oxidation of **1-Pu** and draw comparisons to the Np counterpart from an electronic structure perspective (See SI for details). The molecular orbital (MO) diagram of **2-Pu** depicts five metal-dominant singly occupied frontier MOs (α-density), qualitatively describing unpaired 5*f* electrons on Pu, consistent with the expected 5*f*^5^ configuration for the Pu^3+^ species (Figure S44). In contrast, MOs with substantial Pu atomic orbital (AO) contributions in **1-Pu** reside 0.6–0.9 eV below the highest occupied molecular orbital (HOMO), displaying significant mixing with ligand orbitals (Table S11). Adaptive natural density partitioning (AdNDP)^58^ analysis of **1-Pu** was performed to delineate metal contributions to the localized nonbonding elements from those describing Pu–N bonding. Four one-center one-electron elements with an occupation number (ON) of 1.00 |e| were recovered, confirming a Pu^4+^ center with a 5*f*^4^ configuration (Figure S50).

Subsequent oxidation of **1-Pu** leads to the experimentally transient Pu^5+^ complex [**1-Pu**]^+^, giving rise to either metal-oxidized ([**1-Pu**]^M+^) or ligand-oxidized (**[1-Pu]**^L+^) species, which are structurally distinct local minima with stable wave functions (Figure S51). [**1-Pu**]^M+^ exhibits a contraction of all four Pu–N bonds by 0.068 Å (Table S9), similar to the metal–ligand bond contraction in the recently reported [Np^5+^(NPC)_4_]^+^ due to the Np^5+/4+^ oxidation (0.071 Å exp./0.085 Å theor.),^38^ indicating a metal-based oxidation. AdNDP analysis of [**1-Pu**]^M+^ recovers three unpaired 5*f* electrons with ON = 0.99–1.00 |e| (Figure S52), consistent with the Pu^5+^ oxidation state, although there is substantial spin density spread across the four N_im_ atoms in [**1-Pu**]^M+^ (e.g., −0.73), which is greater than that seen for [Np^5+^(NPC)_4_]^+^ (e.g., −0.32) (Tables S13, S15). In contrast, [**1-Pu**]^L+^ with four unpaired 5*f* electrons at the Pu^4+^ center is reminiscent of the oxidized complexes of [Ln^3+^(NP*)_4_]^−^ (Ln = Nd, Dy).^59^ Specifically, [**1-Pu**]^L+^ exhibits one elongated (2.402 Å) and three shorter (2.107 Å) metal–ligand bonds, akin to those computed for **3-Pu** (2.435 Å/2.109 Å) and [Np^4+^(NPC)_3_(HNPC)]^+^ (2.434 Å/2.119 Å),^38^ suggesting a ligand-based oxidation at one of the N_im_ atoms in this higher energy local minima (Table S9). Thermodynamically, [**1-Pu**]^M+^ is 5.7 kcal/mol more favorable than [**1-Pu**]^L+^, which is much smaller than the 21.3 kcal/mol difference seen for the Np analogues (Figure S45). Based on thermodynamic data, it may be concluded that the PCET reactivity arises from the metal-oxidized species, [Np^5+^(NPC)_4_]^+^ and [**1-Pu**]^M+^, where H^+^ is accepted by the N_im_ atom while the metal center is reduced.

From the MO perspective, the 5*f* orbitals in **1-Pu** are substantially lower in energy relative to [Np^4+^(NPC)_4_] (Figure S46, Table S11). The ligand-dominant orbitals from HOMO to HOMO–10 in **1-Pu** suggest that further oxidation may likely occur at the ligand. However, as was shown previously for [Ln^3+^(NP*)_4_]^−^ (Ln = Pr, Nd, Dy) based on the PBE0 hybrid DFT framework,^59^ metal-based oxidation is still feasible for Pr, with ligand-based oxidation (Nd, Dy) occurring only if the metal-dominant orbitals are buried sufficiently deep in energy. In this context, and using the same methodology, MO energy diagrams predict higher propensity for ligand-based oxidation compared to [Np^4+^(NPC)_4_], which has three metal-dominant singly occupied 5*f* orbitals residing 0.2–0.4 eV above the ligand-dominant orbitals. At first glance, the greater drive to form [**1-An**]^L+^ species for Pu compared to Np may suggest a correlation with the higher rate of PCET reactivity of the former. However, based on calculated redox potentials, it is appreciably harder to oxidize the ligand than the metal for both [Np^4+^(NPC)_4_] and **1-Pu**, and the metal-based oxidation aligns better with experimental redox potentials (Table S17). The higher metal-dominant MOs of [Np^4+^(NPC)_4_] are also congruent with the more facile metal-based oxidation (Table S18), with the Np^5+/4+^ couple (−0.70 V exp./–0.75 V theor.) cathodically shifted compared to the Pu^5+/4+^ couple (−0.32 V exp./–0.16 V theor.).

The Pu^4+/3+^ wave is observable within the electrochemical window unlike for U and Np.^37^ The M^4+/3+^ couple for **1-Pu** (*E*_1/2_ = −2.83 V) is quasi-reversible in contrast to that observed for [Ce(NPC)_4_] (*E*_1/2_ = −2.60 V, Figure S28). This dichotomy is suggestive of a smaller reorganization required for oxidation/reduction of **1-Pu**. The irreversibility of the Pu^5+/4+^ couple is notable in comparison to the quasi-reversible couples observed for Np^5+/4+^ (*E*_1/2_ = −0.70 V) and U^5+/4+^ (*E*_1/2_ = −1.57 V).^37^ The degree of structural rearrangement upon oxidation/reduction can be tracked in the calculated electron detachment energy (DE) or electron affinity (EA) accounting for either vertical (VDE/VEA, no rearrangement) or adiabatic (ADE/AEA, total relaxation) electron transfer (Table S18, see the SI for details). It was previously shown that the appreciable differences between vertical and adiabatic electron transfer in the Ce^4+/3+^ couple correlate with the substantial structural rearrangements occurring upon oxidation [ΔDE = VDE–ADE, 0.90 V] and reduction [ΔEA = VEA–AEA, 0.79 V], providing explanation for the irreversibility of the Ce^4+/3+^ couple.^37^ However, appreciably smaller values are found for the Np^5+/4+^ (ΔDE = 0.42 V; ΔEA = 0.49 V) and Pu^4+/3+^ (ΔDE = 0.69 V; ΔEA = 0.55 V) couples, in agreement with the quasi-reversible event for both the oxidation and reduction. In both cases, the ΔDE and ΔEA values are comparable to each other, suggesting a similar degree of structural rearrangement required for oxidation/reduction. In contrast, the reduction of the Pu^5+/4+^ couple requires noticeably more structural rearrangement (ΔEA = 0.64 V) than oxidation (ΔDE = 0.35 V), which may contribute to its irreversibility.

Upon direct protonation or oxidation and subsequent PCET reaction of **1-Pu** to form **3-Pu**, MOs with substantial Pu AO contribution are shifted lower in energy compared to **1-Pu** by about 0.5–1.1 eV (Figure S44). This effect is similar to what was seen in the Np counterparts,^38^ where MOs with substantial Np AO contribution in [Np^4+^(NPC)_3_(HNPC)]^+^ are intermediate to [Np^4+^(NPC)_4_] and [Np^5+^(NPC)_4_]^+^. To thermodynamically probe the observed PCET reactivity, calculations were performed on the bond dissociation free energy (BDFE) of the N–H bond formed in **3-Pu** (96.1 kcal/mol, Figure 4), which was found to be larger than in the Np counterpart (83.5 kcal/mol). A higher p*K*_a_ value is obtained from the Bordwell equation^60−62^ for **3-Pu** compared to its Np counterpart (27.4 vs. 24.6, Table S19). These changes reflect the increased thermodynamic driving force for the Pu PCET reaction.

**Figure 5:**
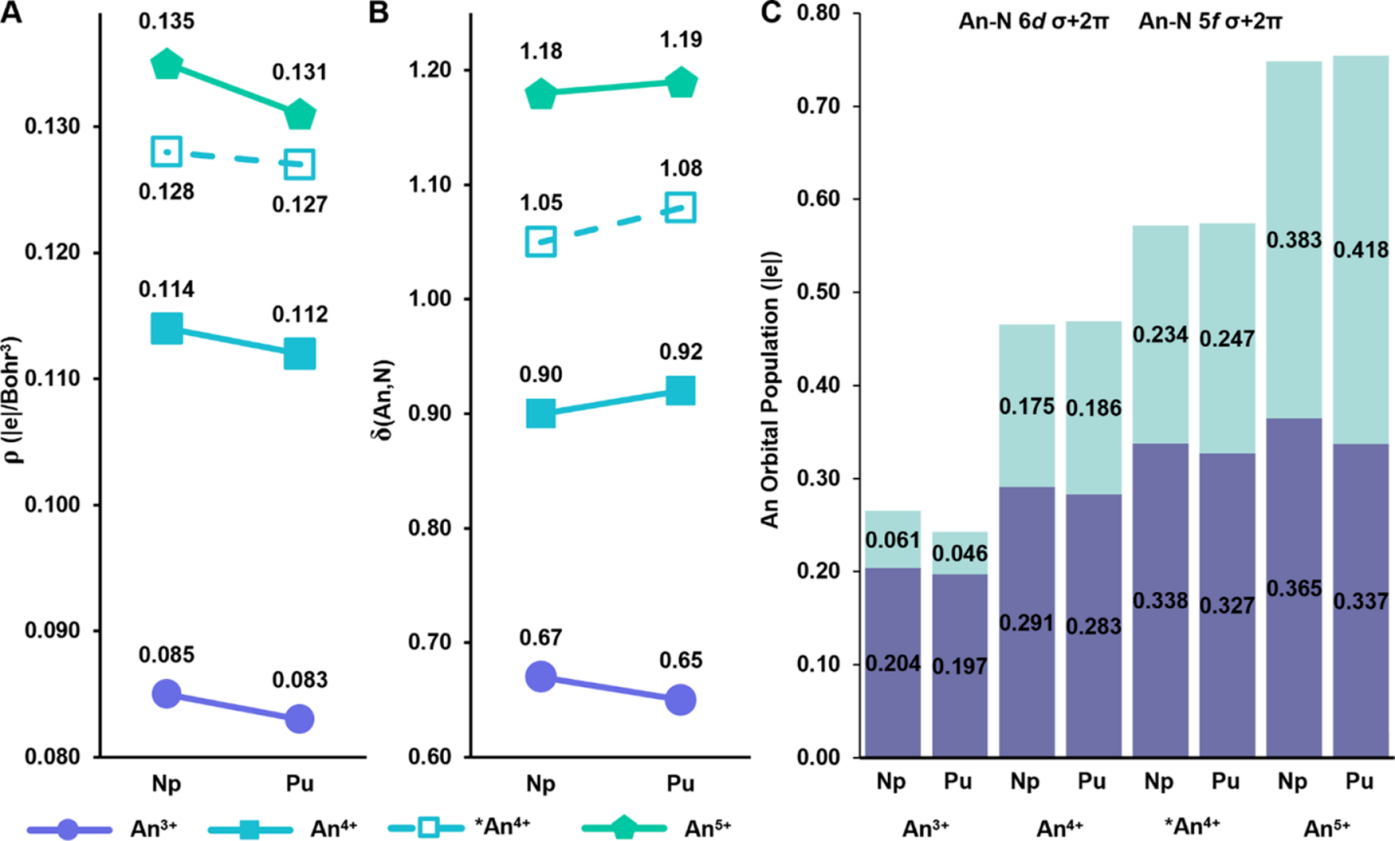
(A) Average total electron density (ρ) at the An–N bond critical point, (B) average delocalization indices (δ(An,N)), and (C) average An orbital population (|e|) for the An–N bonds. The following formula was applied to calculate the An contribution: ON (|e|) of An–N_im_ bond × An–N_im_ bond polarization (An, %) × An (6*d*/5*f*) character (%). An^*n*+^ = [An^*n*+^(NPC)_4_]; *An^4+^ = [An^4+^(NPC)_3_(HNPC)]. See SI for the An contribution to the An–NH bonds.

To understand the role of metal orbital participation in the An–N bonding in relation to the changes in N–H bond strengths and p*K*_a_ (and indirectly to the observed changes in reaction rate) between the Np and Pu complexes, analyses employing the quantum theory of atoms in molecules (QTAIM)^63^ and AdNDP methods were performed. The role of orbital overlap vs. energy degeneracy driven covalency^4,12,64−73^ were assessed as a function of the metal oxidation state and metal identity. It was previously shown that the QTAIM’s total electron density at the bond critical point, ρ, describes orbital overlap, while the delocalization index, δ(An,N), may be employed as an indicator of both orbital overlap and energy degeneracy driven covalency.^64,67,71−73^ As expected, the ρ values (|e|/Bohr^3^) increase from 0.083 to 0.131 in the Pu complexes as metal oxidation state increases from 3+ to 5+. Values for the unprotonated ligands of **3-Pu** (0.127) are intermediate to **1-Pu** and [**1-Pu**]^M+^, similar to the trends seen with the Np counterparts.^37,38^ However, these values for all Pu complexes are slightly smaller than their respective Np counterparts (Figure 5A, Tables S20–S21), in agreement with the smaller 5*f* orbital extent of Pu relative to Np. Average δ(An,N) values for **2-Pu** and [Np^3+^(NPC)_4_]^−^ replicate this trend as well (0.65 vs. 0.67), suggesting negligible contributions from the energy degeneracy driven covalency for the An^3+^ species. This result agrees with the position of the 5*f*-dominant An MOs in both species, which are higher in energy and well separated from the ligand-dominant MOs by about 0.9–2.2 eV (Figure S48).

As the 5*f* orbitals get lower in energy upon oxidation or protonation, the average δ(An,N) increases. It follows that these values are slightly higher in Pu complexes (0.92–1.19) (Figure 5B, Tables S20–S21) than their respective Np counterparts (0.90–1.18) as well. The decreasing trends in the ρ values and increasing trends in δ(An,N) from Np to Pu in these species suggest that the contribution to the covalency from the energy degeneracy between metal and ligand orbitals increases. Qualitatively, this correlates with the greater 5*f* orbital energy lowering in Pu compared to Np, suggesting their increased role in the An–N bonding for the former in higher oxidation states.

To assess the An 5*f*/6*d* contributions to these bonds, AdNDP analysis was performed. In agreement with the QTAIM data, AdNDP total metal contribution in the An–N bonding (σ+2π, see Figure S54) shows that **2-Pu** is less covalent than its Np^3+^ counterpart, with both 5*f* and 6*d* contributions slightly decreasing from Np to Pu (Figure 5C). Likewise, AdNDP data for the higher-valent/protonated **1-Pu**, **3-Pu**, and [**1-Pu**]^M+^ echoed the QTAIM results, confirming that the energy match between the An 5*f* and N 2*p* orbitals facilitates a greater covalency. While the An 6*d* orbital population decreases from Np to Pu for all species in concordance with the decreasing orbital overlap, the 5*f* contributions are slightly larger for Pu than for Np species. The 5*f* contributions also become more prominent as the oxidation state increases or protonation occurs: 0.175 |e| vs. 0.186 |e| for An^4+^, 0.234 |e| vs. 0.247 |e| for [An^4+^(NPC)_3_(HNPC)]^+^, and 0.383 |e| vs. 0.418 |e| for An^5+^. The increase in 5*f* contributions outweighs the decrease in 6*d* contributions, thus, excepting the 3+ oxidation state, all Pu complexes have a higher total 6*d*+5*f* orbital contribution than their Np counterparts (Tables S22–S23). QTAIM shows that the metal-ligand orbital energy match also facilitates an accumulation of the electron density at the N_im_ nuclear critical point (NCP). ρ at the N_im_ NCP is larger for Pu than for Np in the 4+ and 5+ oxidation states (Table S20), in accordance with the NPA spin density on N_im_ (Table S15). For the An^4+^ species, this result correlates with the higher p*K*_a_ values for **3-Pu** in comparison to [Np^4+^(NPC)_3_(HNPC)]^+^ (27.4 vs. 24.6). In the case of the An^5+^ species, this result supports the higher computed spin-orbit coupling (SOC) corrected N–H BDFE values for the Pu PCET product (96.1 kcal/mol) compared to the Np PCET product (83.5 kcal/mol).^38^ Therefore, the pronounced metal-ligand energy degeneracy found in the higher-valent An^5+^ species not only leads to higher overall covalency due to the increased An 5*f* contributions, but also helps facilitate greater electronic charge accumulation on the N_im_ atoms, which may be an important factor for determining relative changes in chemical reactivity between Np and Pu.

## Conclusions

This work presents the synthesis of three imidophosphorane plutonium complexes in the trivalent and tetravalent oxidation states. Cyclic voltammetry studies of the tetrahomoleptic Pu^4+^ species (**1-Pu**) reveal a Pu^5+/4+^ couple which is nearly reversible only at high scan rates. Electrochemical modeling and kinetic analysis were employed to show that the electrochemically generated Pu^5+^ species rapidly undergoes a PCET reaction in THF, generating the protonated Pu^4+^ species **3-Pu**. This reaction occurs at a rate 5 orders of magnitude faster than that of the Np analogue, and the newly formed N–H bond has a calculated BDFE of about 12 kcal/mol greater than the Np PCET product.

DFT studies in conjunction with these thermodynamic and kinetic data implicate changes in the role of 5*f* and 6*d* orbital participation in the An–N_im_ bonding and the electron density on the N_im_ atom as an important component of the observed reactivity differences. Lower 5*f* orbital energies for the Pu species than for their Np analogs are found, lowering more upon oxidation of the metal or protonation of a ligand, and correlating with greater participation of these orbitals in the An–N bonding via energy degeneracy driven covalency. In the lower-valent (Np/Pu)^3+^ complexes, the covalency of the An–N bonding is primarily controlled by the overlap of the An 5*f*/6*d* and N 2*p* orbitals, decreasing from Np to Pu. In contrast, the higher-valent (4+, 5+) species of Pu exhibit slightly increased covalent interactions due to the better energy match between the An and ligand orbitals.

These results reveal new opportunities for kinetic discrimination between actinide elements, and the development of novel separation processes (akin to recent redox-based approaches for the separation of americium^74,75^). Additionally, understanding kinetic differences in fundamental reaction pathways may inform environmental isotope migration modeling.^76^ More comprehensive and systematic computational studies across the An series comparing the trends in PCET reactivity, BDFE, covalency, and redox properties are underway.

## Supporting Information Available

Experimental and computational details, X-ray crystallographic, spectroscopic, and electrochemical kinetic studies (PDF)
