# Supplementary material for: Proton-Coupled Electron Transfer at the Pu5+/4+ Couple
Source: J Am Chem Soc. 2024 Jul 25;146(31):21859–67. doi: 10.1021/jacs.4c06319 (PMC11311234; doi:10.1021/jacs.4c06319)
Supplement: Supplementary file 1 — ja4c06319_si_001.pdf [file ja4c06319_si_001.pdf]

Supporting Information For

Proton-Coupled Electron Transfer at the $Pu^{5+/4+}$ Couple

Kaitlyn S. Otte,[†] Julie E. Niklas,[†] Chad M. Studvick,[§] Charlotte L. Montgomery,[◊] Alexandria R. C. Bredar,[◊] Ivan A. Popov,*[#] and Henry S. La Pierre*[†,‡,||].

†School of Chemistry and Biochemistry, Georgia Institute of Technology, Atlanta, Georgia 30332-0400, United States

§Department of Chemistry, University of Akron, Akron, Ohio 44325-3601, United States

◊Department of Chemistry, University of North Carolina at Chapel Hill, Chapel Hill, North Carolina 27599-3290, United States

#Department of Chemistry, Washington State University, Pullman, WA 99164, United States of America

‡Nuclear and Radiological Engineering and Medical Physics Program, School of Mechanical Engineering, Georgia Institute of Technology, Atlanta, Georgia 30332-0400, United States

||Physical Sciences Division, Pacific Northwest National Laboratory, Richland, Washington 99352, United States

Table of Contents

General Considerations 2

Experimental 3

UV-vis-nIR Spectroscopy 8

NMR Spectroscopy 13

Electrochemical Studies 19

Kinetic Analysis 24

Single-Crystal X-ray Diffraction 33

DFT Calculations 37

References 54

General Considerations:

Unless otherwise noted, all reagents were obtained from commercial suppliers and all syntheses and manipulations were conducted under nitrogen with exclusion of oxygen and water in an inert atmosphere negative pressure Vac glovebox in the dedicated Georgia Tech Transuranic Synthetic Facility (TSF). All glassware was oven-dried at ca. 160 °C for at least 8 h prior to use. Celite and molecular sieves were dried under vacuum at a temperature >250 °C for over 24 h.

Materials: C_6D_6 (CIL) was stored over 3 Å molecular sieves and then vacuum-transferred from purple sodium/benzophenone and stored on fresh sieves prior to use. THF-d_8 (CIL) was degassed by 3 freeze-pump-thaw cycles and dried over sodium metal, then vacuum transferred, and stored over 3 Å molecular sieves. Diethyl ether, *n*-pentane, *n*-hexane, and tetrahydrofuran were purged with UHP-grade argon (Airgas) and passed through columns containing Q-5 and molecular sieves in a solvent purification system (JC Meyer Solvent Systems). All solvents in the glovebox were stored in bottles over 3 Å molecular sieves. [^{n}Bu$_4$N][BPh$_4$] (Sigma-Aldrich, 99% or electrochemical grade >99%) was recrystallized twice from 10:90 water:acetone followed by a final recrystallization from 30:70 diethyl ether:acetone, then dried under vacuum at 100˚C for a minimum of 24 h prior to use. KNPC (NPC = [NP^{t}Bu(pyrr)$_2$]$^-$; ^{t}Bu = C(CH$_3$)$_3$; pyrr = pyrrolidinyl)[1] and KC_8[2] were prepared according to literature procedure. Immersion oil (Cargille Type NVH) was degassed on a Schlenk line by stirring under active vacuum with gentle heating (to facilitate stirring, ca. 40°C) overnight prior to use.

Caution! ^{242}Pu is a strong α-emitter ($t_{1/2}$ = 3.76x10^5 years). This species poses serious health risks if not properly contained and handled. PuO_2 was obtained through the ORNL NIDC. All manipulations with ^{242}Pu were performed in the dedicated Georgia Tech Transuranic Synthetic Facility (TSF) with proper radiological controls for the safe handling of this isotope. All free-flowing solids and air-sensitive solutions were handled in an inert atmosphere negative pressure glovebox, and air-stable solutions in a dedicated reprocessing hood. Spectroscopic analyses of Pu samples were performed in the TSF or in user facilities (UV-vis-nIR and NMR) using appropriate containment to remove the sample from the glovebox. Clean swipes of sample exteriors, and primary containment as appropriate, were confirmed using a Ludlum 3030 α/β counter prior to analysis of samples.

Analytical: Single-crystal X-ray diffraction analysis of Pu samples required transport of samples to another building under containment. Due to the radiological hazards associated with ^{242}Pu, elemental analyses were not performed on Pu samples. Pu samples were transported under triple containment to a Bruker Advance III 400 MHz NMR at 298 K utilizing a 3-mm Teflon NMR tube liner with two Teflon plugs inside a standard glass NMR tube with plastic cap (see page S11).[3] ^{1}H NMR spectra are referenced to the ^{1}H resonances of the residual protiosolvent. ^{13}C{^{1}H} NMR spectra are referenced to the ^{13}C resonance of the deuterated solvent. ^{31}P{^{1}H} NMR spectra are referenced to an external standard. Peak position is listed, followed by peak multiplicity, integration value, coupling constant (Hz), and proton or carbon assignment, where applicable. Multiplicity and shape are indicated by the following abbreviations: s (singlet); d (doublet); t (triplet); q (quartet); dd (doublet of doublets); td (triplet of doublets); m (multiplet); b (broad). ^{1}H, ^{13}C{^{1}H}, and ^{31}P{^{1}H} NMR chemical shifts are reported in δ, parts per million. UV-vis-nIR spectroscopy was performed in-house in a small-volume (1.4 mL) quartz cuvette sealed with a Teflon-lined screw cap using an Agilent Cary 60 spectrophotometer or transported under triple containment to a Hitachi UH4150 UV-vis-nIR scanning spectrophotometer between 2400-200 nm when specified. Electrochemical methods are detailed in the Electrochemical Studies section.

Experimental:

Scheme S1. Synthetic routes to **1-Pu**, **2-Pu**, and **3-Pu**.

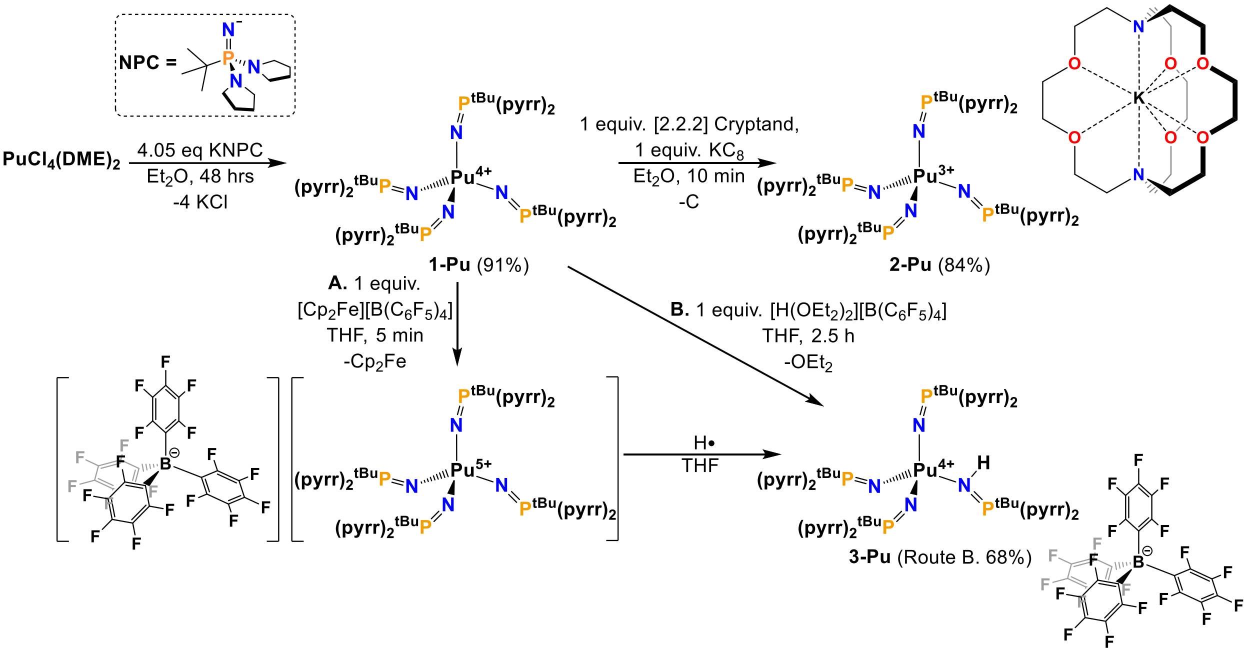

Synthesis of $[Pu^{4+}(NPC)_4]$, (1-Pu).

Inside a negative pressure glovebox, $PuCl_4(DME)_2$ (0.060 g, 0.106 mmol) was dissolved in 3 mL of diethyl ether in a 20 mL scintillation vial charged with a stir bar. The KNPC (0.121 g, 0.429 mmol) was added dropwise as a suspension in 3 mL of diethyl ether and residual solid transferred by rinsing the vial with an additional 5 mL of solvent. Immediately on addition of ligand, the solution turned pink and gradually evolved to an opaque swampy green. The mixture was stirred for 48 hours at room temperature. Volatiles were removed *in vacuo*, and the residue was then triturated three times with 1 mL of *n*-pentane. The green residue was taken up in 3 mL of *n*-pentane and filtered through a pipette packed with glass fiber and Celite, and the frit was rinsed with two additional 1 mL aliquots of *n*-pentane. The green-red dichroic solution was concentrated *in vacuo* and placed in a -35 °C freezer. Overnight, Shrek-green XRD quality crystals grew, which were observed to have a slight pink dichroic behavior under the microscope (0.117 g, 91%). ^{1}H NMR (400 MHz, C_6D_6): δ 3.06 (32 H, s, $C_\beta\underline{H}_2$), 1.39 (32 H, d, $C_\alpha\underline{H}_2$), 1.27 (36 H, d, $C\underline{H}_3$). $^{31}P\{^{1}H\}$ (162 MHz, C_6D_6): δ 139.80. $^{13}C\{^{1}H\}$ (101 MHz, C_6D_6): δ 42.19 (d, J = 104.5 Hz, $\underline{C}CH_3$), 36.76 (s, $\underline{C}_\beta H_2$), 24.55 (d, J = 5.5 Hz, $\underline{C}_\alpha H_2$), 18.69 (s, $\underline{C}H_3$).

Synthesis of $[Pu^{3+}(NPC)_4]$[K- 2.2.2.-cryptand], (2-Pu).

To a 20 mL scintillation vial containing **1-Pu** (0.024 g, 0.020 mmol), 0.5 mL of diethyl ether was added. A suspension of KC_8 (0.003 g, 0.020 mmol) in 0.5 mL of diethyl ether was added to 2.2.2. cryptand (0.008 g, 0.020 mmol). This mixture was then added dropwise to the vial containing **1-Pu** and rinsed over with an additional 0.5 mL aliquot of diethyl ether. Upon addition, a subtle color

change from coffee brown to a green-toned brown was noted, and the reaction was agitated gently by hand for fifteen minutes. The suspension was filtered through a pipette packed with glass fiber and Celite, affording a golden and red dichroic filtrate. The filtrate was concentrated *in vacuo*, then triturated with two 0.5 mL aliquots of *n*-pentane. The residue was redissolved in 2 mL of diethyl ether and concentrated *in vacuo* prior to being moved to a -35 °C freezer to crystallize overnight affording red and yellow dichroic XRD quality crystals. The remaining volatiles were removed *in vacuo* to give **2-Pu** (0.027 g, 84%).

Synthesis of [Pu^{4+}(NPC)$_3$(HNPC)][B(ArF_5)$_4$], (3-Pu).

A. A 20 mL scintillation vial was charged with a stir bar, **1-Pu** (0.010 g, 0.008 mmol), and 0.5 mL of tetrahydrofuran. Ferrocenium tetrakis(pentafluorophenyl)borate [Cp_2Fe][B(ArF_5)$_4$] (0.007 g, 0.009 mmol) was added dropwise to the reaction vial in 0.5 mL of tetrahydrofuran. Upon addition, the solution rapidly clarified and became dark amber-brown. The reaction was left stirring for 5 minutes, then volatiles were removed *in vacuo*. The reaction was rinsed with a total of six 1 mL aliquots of *n*-pentane until colorless, scraping the residue with a spatula during last three rinses. The residue was taken up in 1 mL of diethyl ether and moved to a 4 mL vial, which was concentrated to ~0.2 mL, then placed inside a 20 mL vial containing silicon grease (to promote solvent vapor diffusion) prior to being stored at -35 °C. Over the course of one week in the freezer a small quantity of SCXRD quality crystals of **3-Pu** grew.

B. 1-Pu (0.046 g, 0.038 mmol) was dissolved in 0.5 mL of tetrahydrofuran. The oxonium acid [H(OEt_2)$_2$][B(ArF_5)$_4$] (0.032 g, 0.039 mmol) was dissolved in 0.5 mL of tetrahydrofuran and transferred into the reaction vial by pipette, effecting a rapid color change from Shrek-green to amber. The reaction was stirred for 2.5 hours, then volatiles were removed *in vacuo*, and the remaining residue was triturated three times with 1 mL of *n*-pentane. The amber residue was dissolved in diethyl ether and concentrated *in vacuo* to ~ 0.2 mL and stored in a -35 °C freezer for five days to afford crystals of **3-Pu** (0.049 g, 68%). ^{1}H NMR (400 MHz, d_8-THF): δ 5.09 (4H, s, $C_\alpha\underline{H}_2^{ax}$, NPCH), 4.87 (4H, s, $C_\alpha\underline{H}_2^{eq}$, NPCH), 3.15 (1H, bs, N$\underline{H}$, NPCH), 2.56 (9H, s, C$\underline{H}_3$, NPCH), 2.49 (24H, m, $C_\alpha\underline{H}_2$, NPC), 1.78 (4H, m, $C_\beta\underline{H}_2^{ax}$, NPCH), 1.67 (4H, s, $C_\beta\underline{H}_2^{eq}$, NPCH), 1.37 (24H, m, $C_\beta\underline{H}_2$, NPC), 0.60 (27H, d, J = 14.35 Hz, C$\underline{H}_3$, NPC). ^{31}P{^{1}H} NMR (162 MHz, d_8-THF): δ 78.95, 45.62. ^{13}C{^{1}H} (101 MHz, d_8-THF): δ 147.48 (d, J = 410 Hz, B($\underline{C}_6H_5$)$_4$), 38.86 ($\underline{C_\alpha}H_2$, NPC), 31.53 ($\underline{C_\alpha}H_2$, NPCH), 26.35 ($\underline{C_\beta}H_2$, NPCH), 25.37 ($\underline{C_\beta}H_2$, NPC), 25.24 ($\underline{C}CH_3$, NPC), 19.00 ($\underline{C}CH_3$, NPC), 18.90 ($\underline{C}H_3$, NPCH). ^{19}F NMR (376 MHz, d_8-THF): δ -132.66, -165.08, -168.52.

Modified Dissolution Procedure Starting from PuO_2:

This procedure was built off work by Kozimor[4] and Gaunt[5] to follow standard procedures used at Los Alamos National Laboratory. It has been optimized for work conducted in an academic facility over a five-day, 8-h/day work week.

Inside of a negative pressure glovebox, solid PuO_2 (0.038 g) was massed into a 15 mL Falcon tube. After transferring the PuO_2 to a dedicated reprocessing hood, 2 mL of concentrated HCl was added,

followed by 10 μL of concentrated HF. The mixture was gently agitated before heating to 80 °C for 35 minutes at which point the solid had almost completely dissolved. A 1000-mL beaker of 3mm borosilicate beads (preheated approximately 1 h prior) was used as a heating bath and containment. A gentle stream of dry argon was directed over the opening of the Falcon tube (enough to just disturb the surface of the solution) while heating to 80 °C for 2.5 h, until a dry residue was obtained. The residue was taken up in 1.5 mL of 1 M HCl and stored sealed overnight.

The HCl concentration was adjusted to ~6 M, giving a final volume after the adjustment of ~2.7 mL. A valence check was conducted by UV-Vis (40 μL of Pu solution, 960 μL 1.0 M $HClO_4$) which indicated a clean tetravalent sample (Fig. S12). A 6 mL anion-exchange column was prepared by loading 3 mL of AG MP-1 anion resin (50-100 mesh). The column was conditioned with 9 mL of deionized H_2O, followed by 9 mL of 6 M HCl. The Pu solution was loaded onto the column and rinsed with an additional 0.5 mL of 6 M HCl. No bands were visible at this point, but the column residue darkened slightly in color. The column was eluted with ~5 mL of slightly acidic water (50 mL of H_2O mixed with 5 drops of concentrated HCl), and a brown band was observed moving down the column, three total fractions were collected. A final volume of ~7.5 mL solution was retained from the first two fractions. The solution can be stored capped overnight at this point without observing oxidation.

The solution was transferred to a tared 20 mL glass vial, using a minimum volume (< 0.5 mL) of slightly acidic water to rinse the Falcon tube. The glass vial was secured in a 1000-mL beaker of 3mm borosilicate beads, still at 80 °C, and a gentle stream of argon was directed over the neck of the vial (enough to just disturb the surface of the solution). The vial was heated for 5 h until a "dry" residue ($PuCl_4$•(xH_2O)) was obtained. The vial was removed from the beads, cooled, and a cored, unlined, plastic vial cap was used to secure a singly-folded Kim-wipe barrier to the neck of the vial (we find this method not only poses less risk of contamination than affixing the Kim-wipe with a rubber band, but decreases contact time with the sample) then transferred to the antechamber where it remained under vacuum for 18 h. The $PuCl_4$•(xH_2O) (0.044 g), shown in Fig. S3, was used immediately to make $PuCl_4(DME)_2$ following the reported procedure (0.060 g, 76% isotope recovery.)[5]

Synthesis of [Fc][B(ArF_5)$_4$].

The synthesis of [Fc][B(ArF_5)$_4$] was modified from literature.[6] All manipulations preceding drying of the product were conducted with no exclusion of air or moisture, and utilizing benchtop solvents. In a 100-mL round bottom flask with a stir bar, sublimed Cp_2Fe (2.00 g, 0.0107 mol; ground) was added and dissolved in acetone (16 mL), then deionized water (40 mL) was added with stirring, forming a suspension. Solid ferric nitrate ($Fe(NO_3)_3$-$(H_2O)_9$) (5.65 g, 0.0139 mol) was added with stirring (any ferric salt should be suitable for this), immediately effecting a color change to dark green. The step is exothermic. The color gradually turned to dark blue over 20 minutes. The reaction mixture was stirred until all ferrocene was consumed, approximately 45 minutes. The solution was filtered through a pad of Celite in a fine frit and rinsed with acetone (5 mL). A stir bar was added to the filtrate in the receiving flask along with [K][B(ArF_5)$_4$] (7.93 g, 0.0110 mol) as a solid, and stirred for 15 min. Ethanol (20 mL) was added to assist in precipitation

of additional dark blue solid. The dark blue solid was collected on a filter paper via vacuum filtration and rinsed with deionized water (40 mL) and hexanes (20 mL). Some additional precipitate was collected from the filtrate and the combined solids were transferred to a 50-mL Schlenk tube and dried under vacuum in a 60 °C water bath for 48 h. After drying, the [Fc][B(ArF_5)$_4$] (8.90 g, 96%) was stored in a glovebox.

We note that [Fc][B(ArF_5)$_4$] can also be prepared in the glovebox by reaction of [Ag][B(ArF_5)$_4$] and Cp_2Fe on small scales, but yields are poor (<40%).

Synthesis of [H(OEt_2)$_2$][B(ArF_5)$_4$].

The synthesis was adapted from literature.[7] In the glovebox, [K][B(ArF_5)$_4$] (1.78 g, 2.47 mmol) was massed into a 20-mL vial, charged with a teflon stir bar, and dissolved in diethyl ether (6 mL). The solution was stored at -35 °C for 30 min. HCl in Et_2O (2.5 M) was stored in the freezer at -35 °C prior to addition, then added by syringe (6.25 mL, 6 eq, 15.6 mmol), effecting immediate precipitation of colorless solid. The mixture was stirred for 20 min, transferred to the freezer for an additional 20 min, then filtered through a pipette with glass fiber filter and a pad of Celite. The filtrate (nearly-colorless/very pale yellow) was concentrated to approximately 10 mL, at which point crystals were readily forming *in vacuo*. All material was allowed to redissolve, and the solution was stored in the freezer at -35 °C overnight. The remaining solution was decanted into a new vial and concentrated to <1 mL for additional product recovery, and the colorless crystalline material dried *in vacuo* for 30 min (0.662 g). A second batch of product was collected and dried (0.135 g) for a combined yield of 39%.

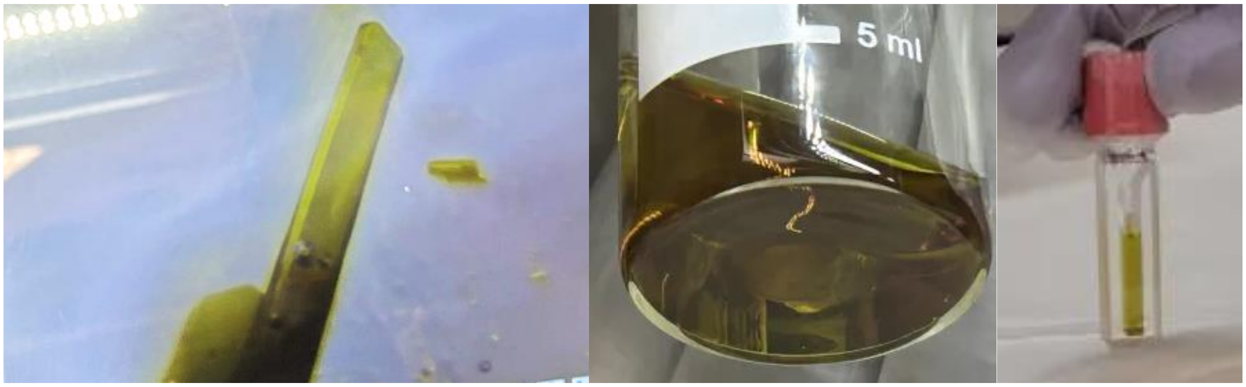

Figure S1. Crystals of **1-Pu** along with two different solution pictures to highlight the red/green dichroism observed for the compound.

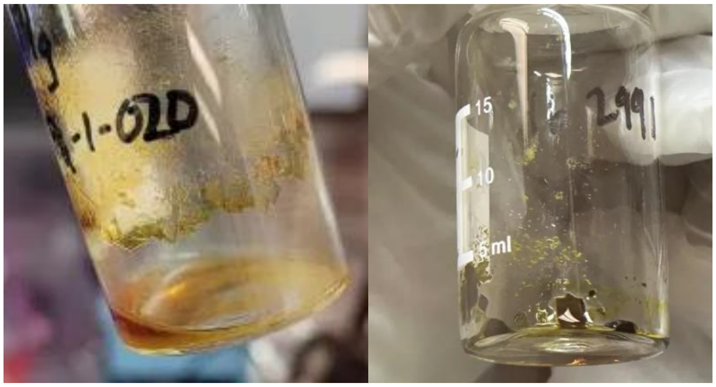

Figure S2. Left shows **2-Pu** as a gold/red dichroic solution. Right is an image of **3-Pu** crystals. Dark red/dark green dichroism was observed.

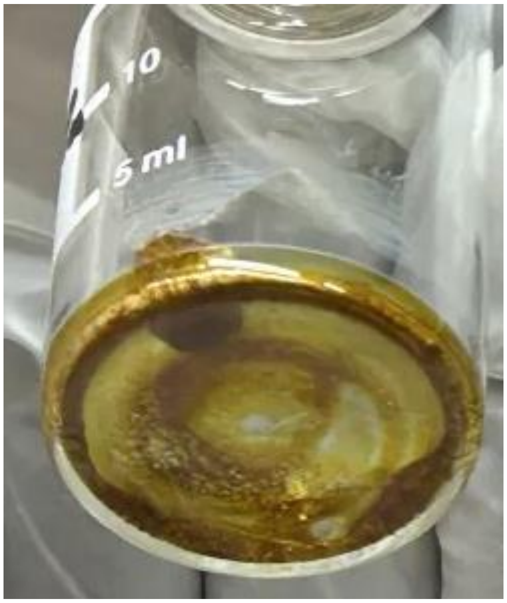

Figure S3. Picture inside glovebox of dried orange/brown $PuCl_4$•(xH_2O) residue.

UV-vis-nIR Spectroscopy

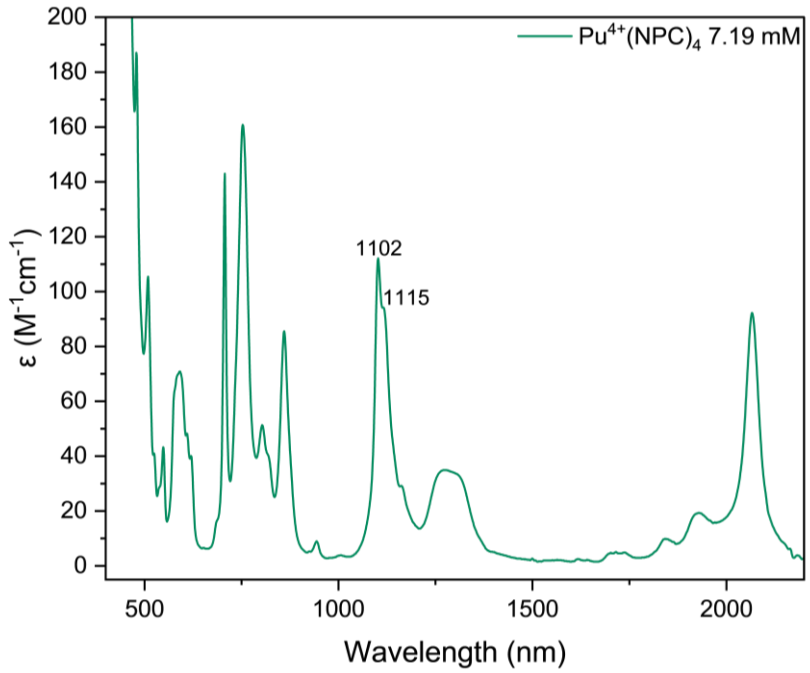

Figure S4. Vis-nIR of **1-Pu** (7.2 mM) in C_6D_6 collected on a Hitachi UH4150 UV-vis-nIR scanning spectrophotometer. Diagnostic peaks of Pu^{4+} near 1,100 nm are labeled.[8]

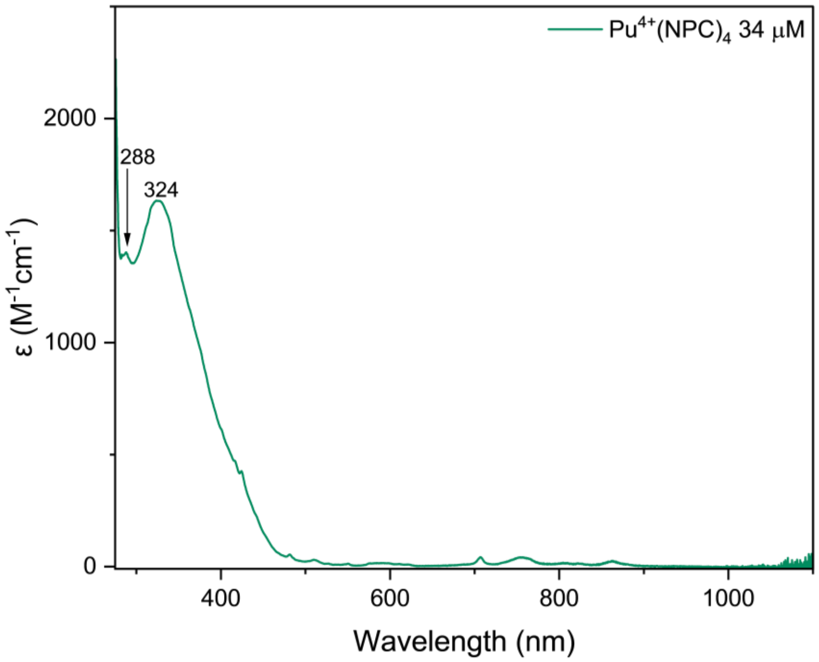

Figure S5. UV-vis-nIR spectrum of **1-Pu** at 0.034 mM concentration in C_6D_6 collected on an Agilent Cary 60 spectrophotometer.

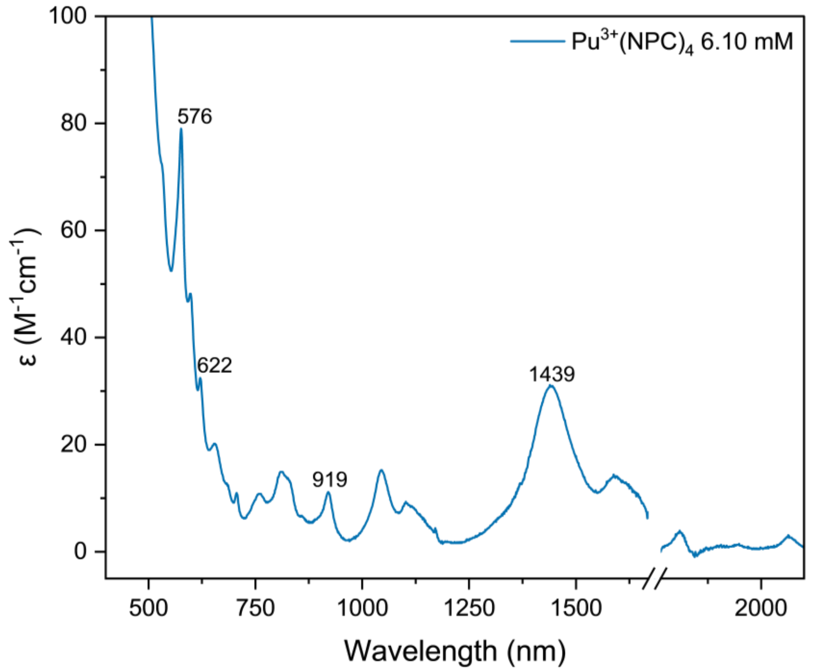

Figure S6. Vis-nIR of **2-Pu** at 6.10 mM concentration in THF collected on a Hitachi UH4150 UV-vis-nIR scanning spectrophotometer. Solvent artifact removed in break on x-axis. Diagnostic peaks of Pu^{3+} near 580, 620, 900, and 1,430 nm are labeled.[8, 9]

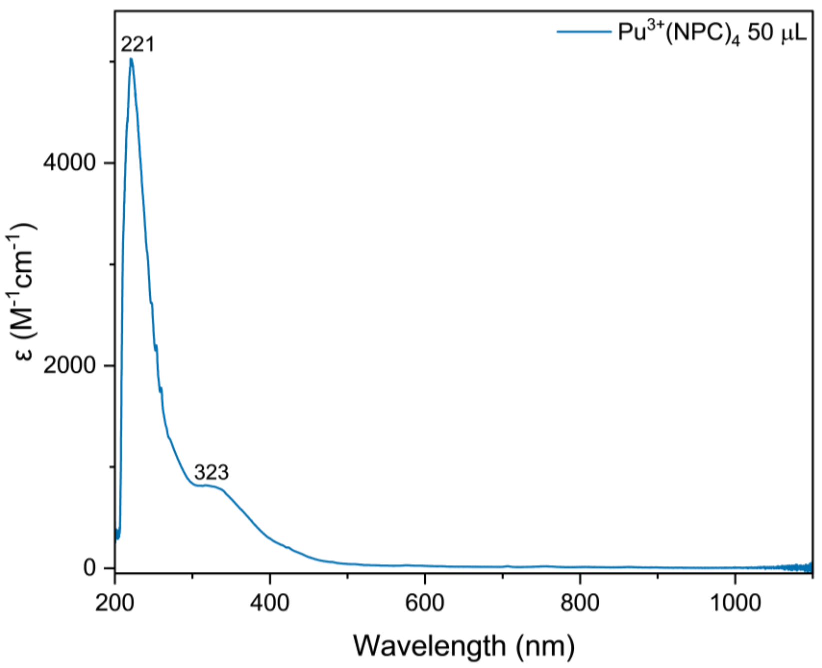

Figure S7. UV-vis-nIR spectrum of **2-Pu** at 0.050 mM concentration in THF collected on an Agilent Cary 60 spectrophotometer.

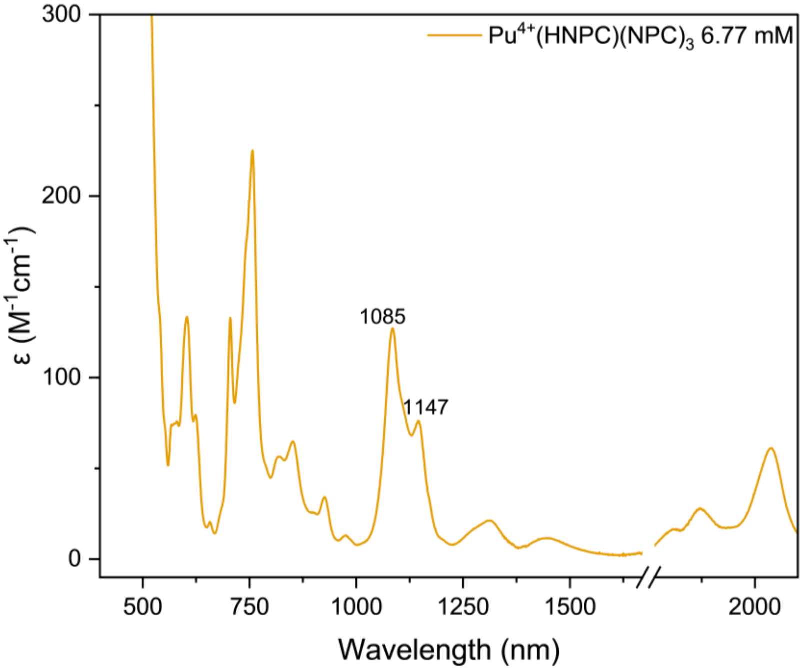

Figure S8. Vis-nIR spectrum of **3-Pu** at 6.77 mM concentration in THF collected on a Hitachi UH4150 UV-vis-nIR scanning spectrophotometer. Solvent artifact removed in break on x-axis. Diagnostic peaks of Pu^{4+} centered near 1,100 nm are labeled.[8]

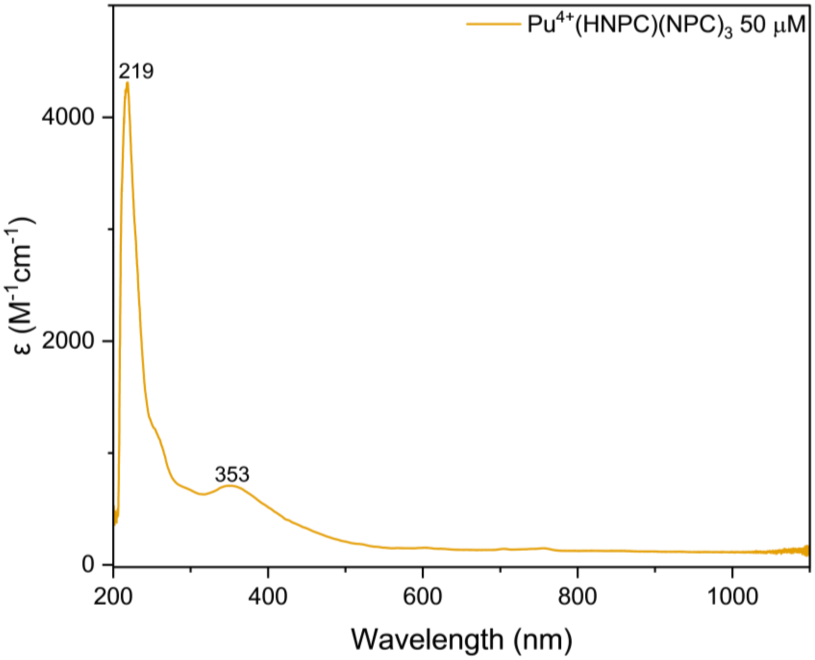

Figure S9. UV-vis-nIR spectrum of **3-Pu** at 0.050 mM concentration in THF collected on an Agilent Cary 60 spectrophotometer.

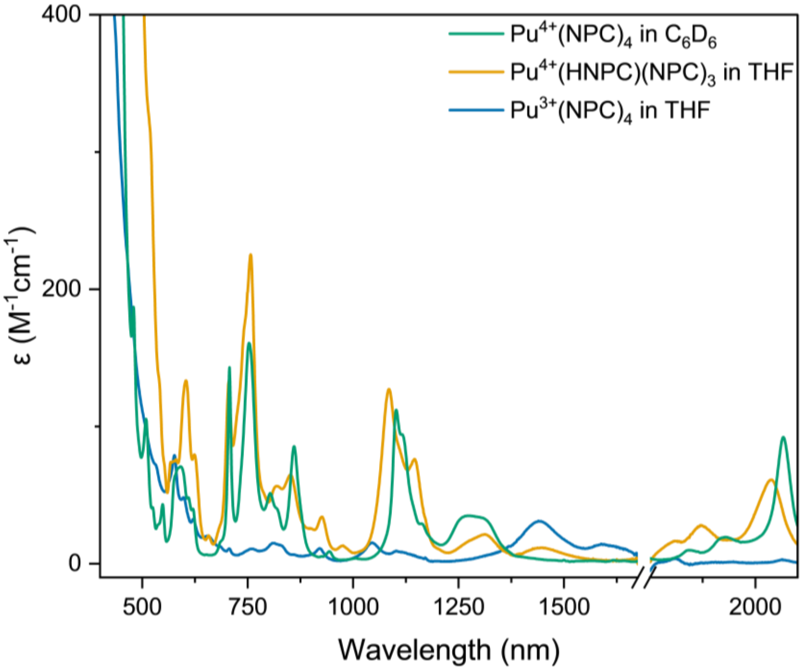

Figure S10. Vis-nIR spectra of **1-Pu**, **2-Pu**, and **3-Pu** at high concentration (~6.5 mM) in C_6D_6 or THF collected on a Hitachi UH4150 UV-vis-nIR scanning spectrophotometer. Solvent artifact removed in break on x-axis.

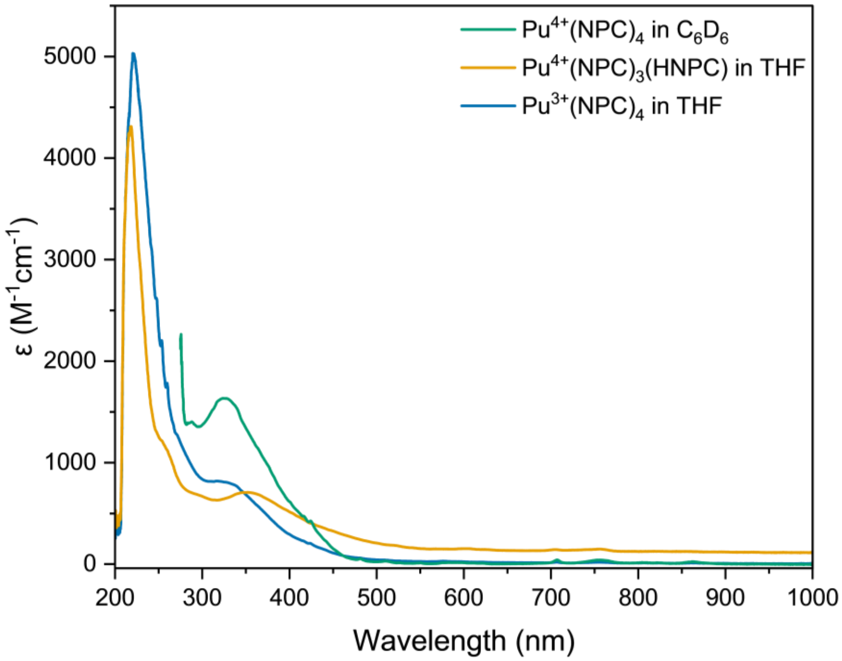

Figure S11. UV-vis-nIR coplot of **1-Pu**, **2-Pu**, and **3-Pu** at low concentration (0.034-0.050 mM) in C_6D_6 or THF collected on an Agilent Cary 60 spectrophotometer.

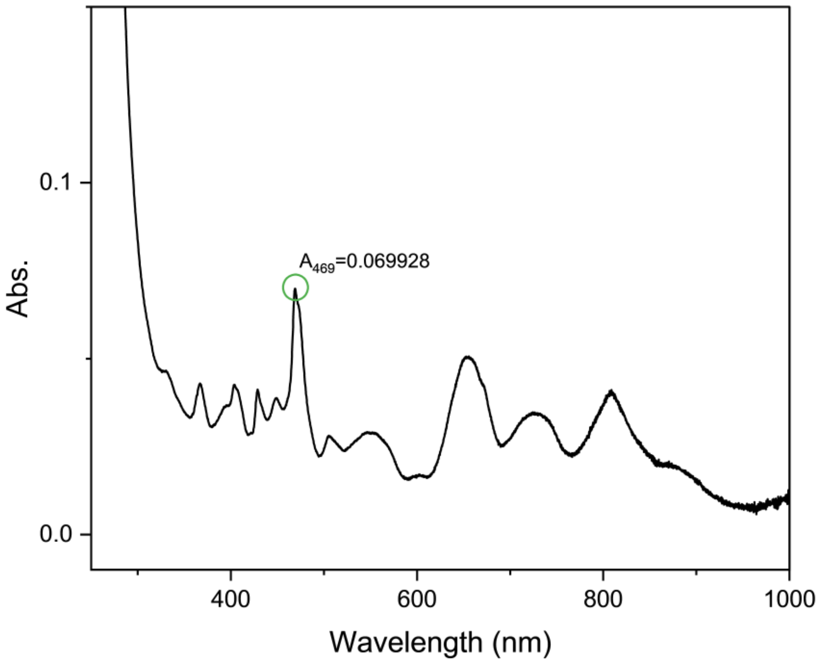

Figure S12. UV-vis-nIR of Pu^{4+} taken during dissolution of PuO_2 in 1 M $HClO_4$, collected on an Agilent Cary 60 spectrophotometer. We note that yields based off extinction coefficients have not been reliable with this setup due to settling of the material in the cuvette, which results in a low estimate of isotope in the sample. This can be mitigated somewhat by gently mixing the cuvette solution with a glass pipette, and/or by using 2 M $HClO_4$.

NMR Spectroscopy

Note: NMR of **2-Pu** ($Pu^{3+}(NPC)_4$) could not be obtained due to immediate degradation of the compound in the presence of Teflon and lack of suitable alternatives to maintain adequate containment.

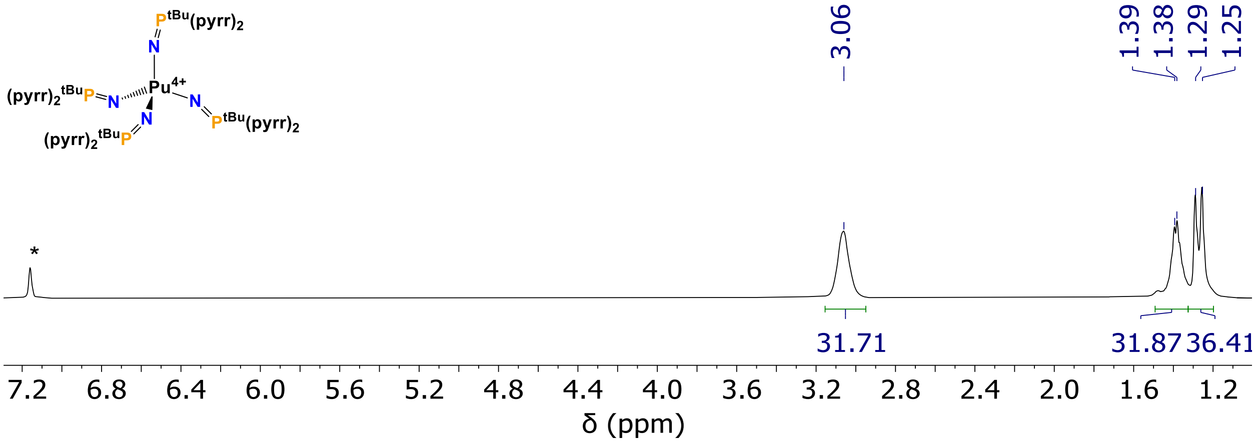

Figure S13. ^1H NMR of **1-Pu** in C_6D_6. Peak of C_6D_5H is noted as *.

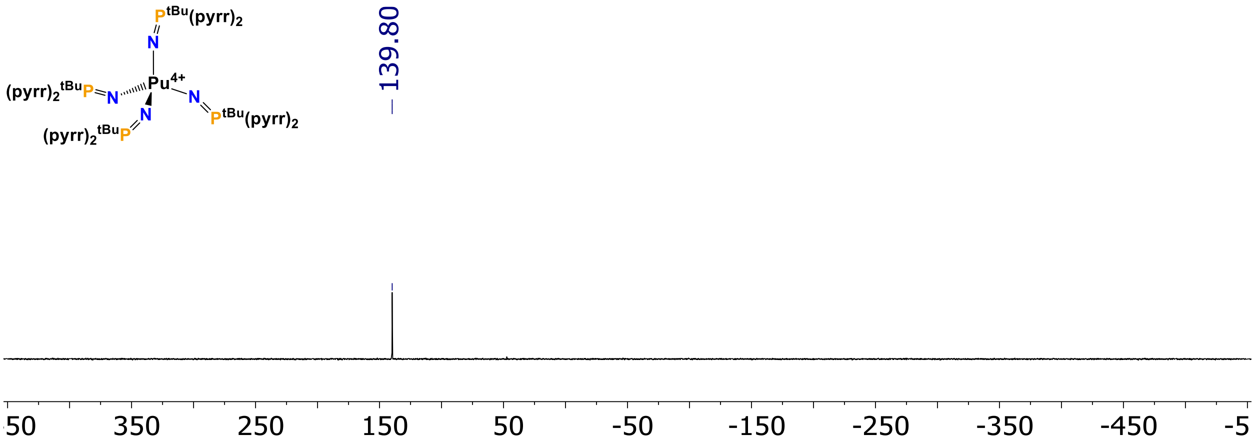

Figure S14. $^{31}P\{^1H\}$ NMR of **1-Pu** in C_6D_6.

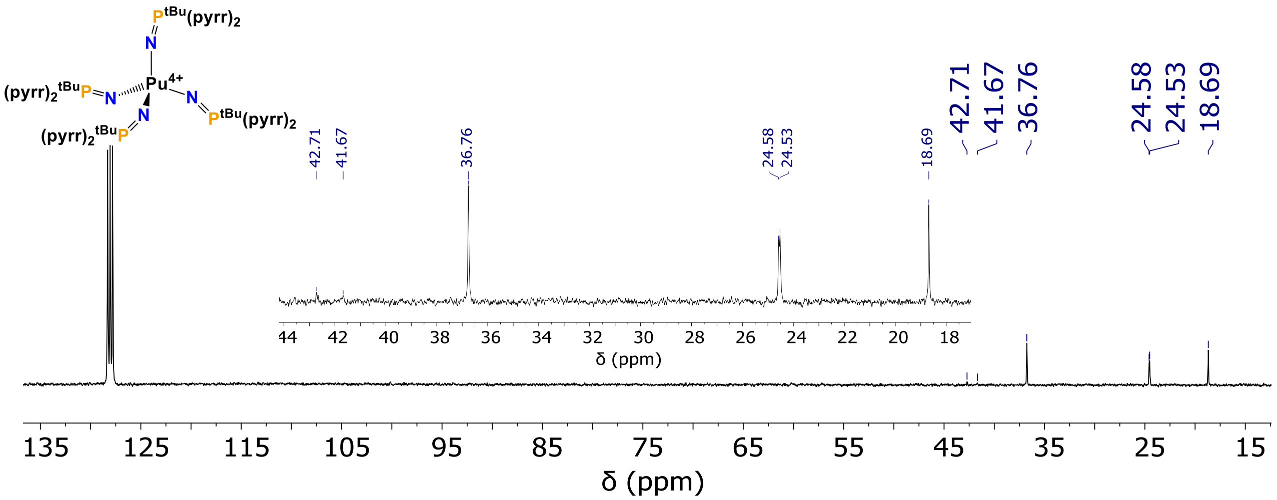

Figure S15. $^{13}C\{^{1}H\}$ NMR of **1-Pu** in C_6D_6. Residual solvent signal is at 128.06 ppm.

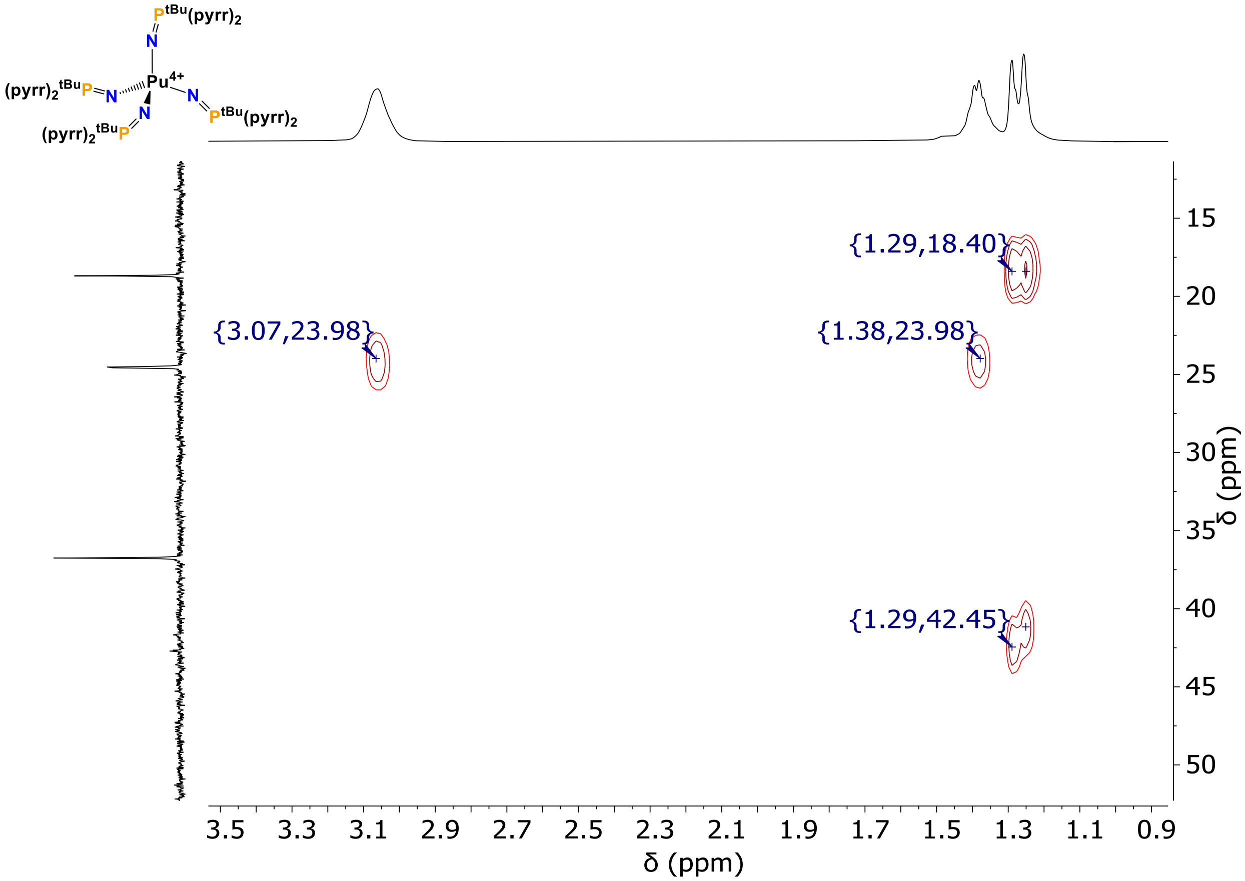

Figure S16. ^{1}H-^{13}C HMBC NMR of **1-Pu** in C_6D_6.

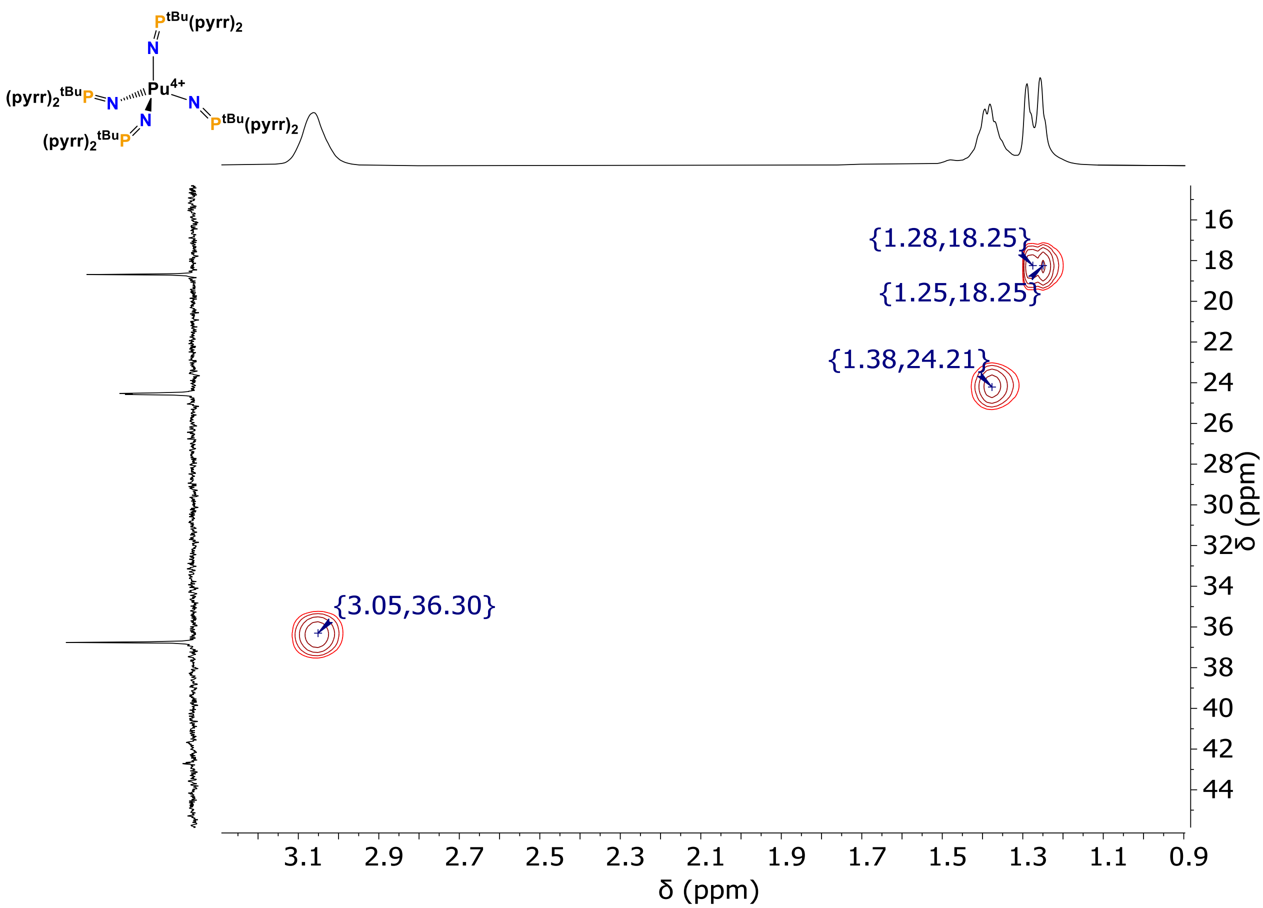

Figure S17. ^{1}H-^{13}C HSQC NMR of **1-Pu** in C_6D_6.

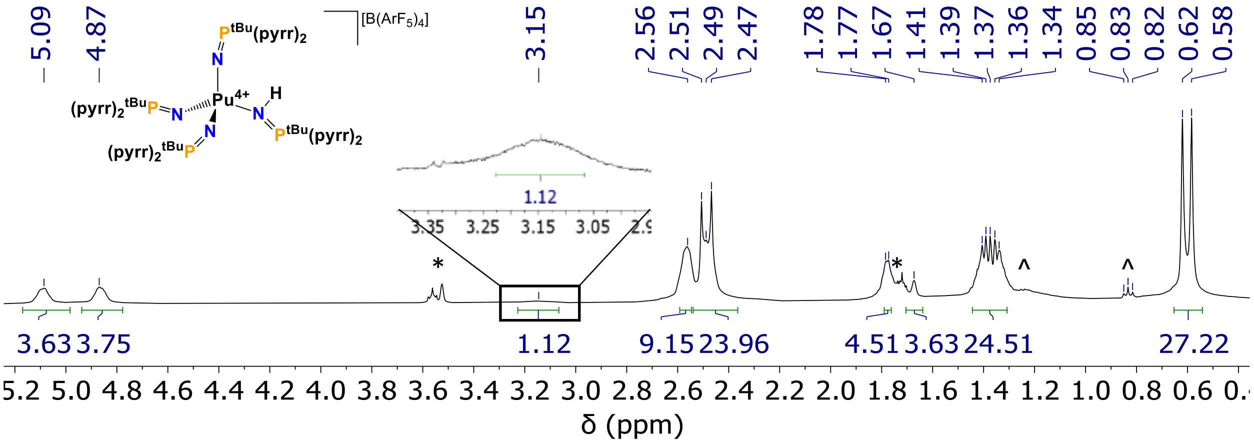

Figure S18. ^{1}H NMR of **3-Pu** in d_8-THF. Residual solvent and protio-tetrahydrofuran are both marked with *, and *n*-pentane is denoted with ^.

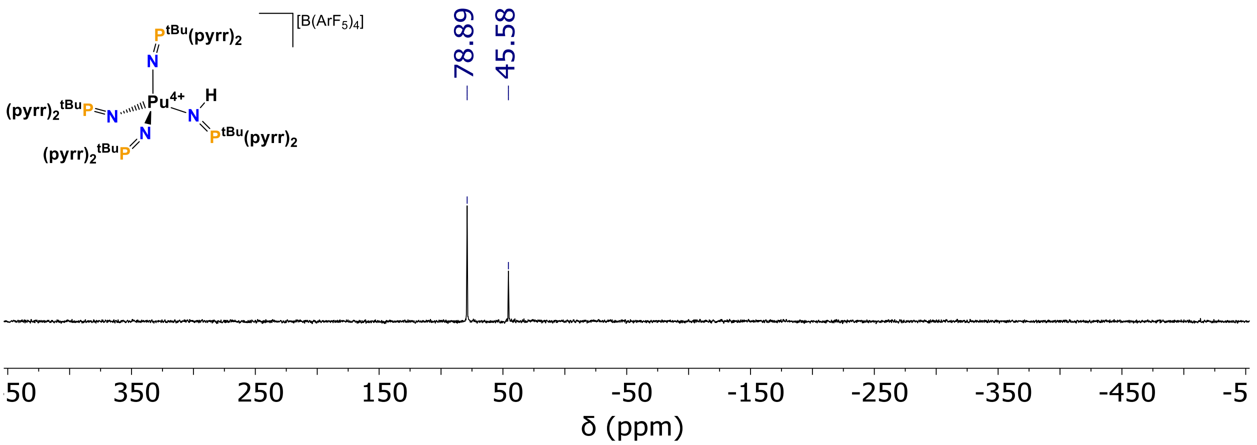

Figure S19. $^{31}P\{^{1}H\}$ NMR of **3-Pu** in d_8-THF.

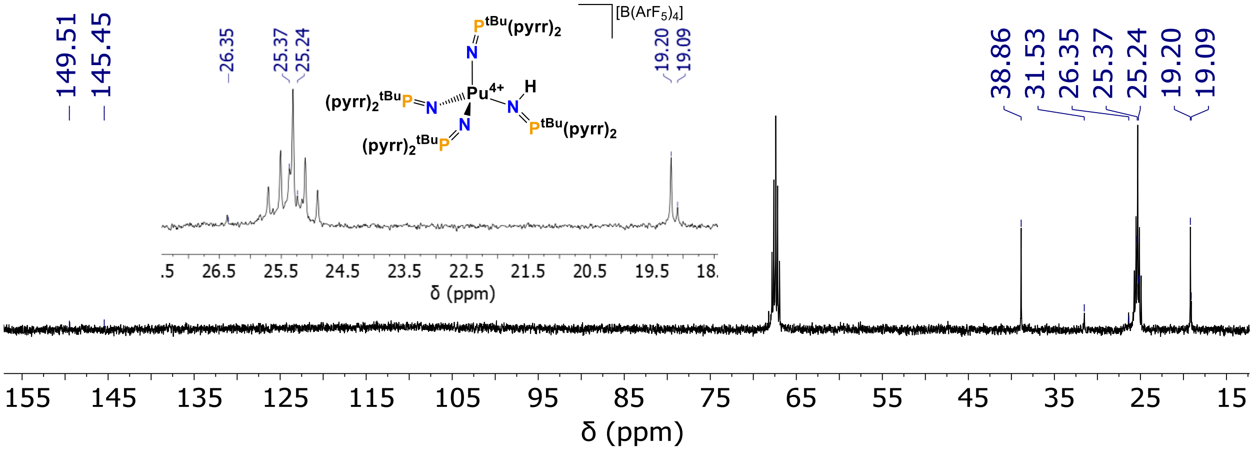

Figure S20. $^{13}C\{^{1}H\}$ NMR of **3-Pu** in d_8-THF.

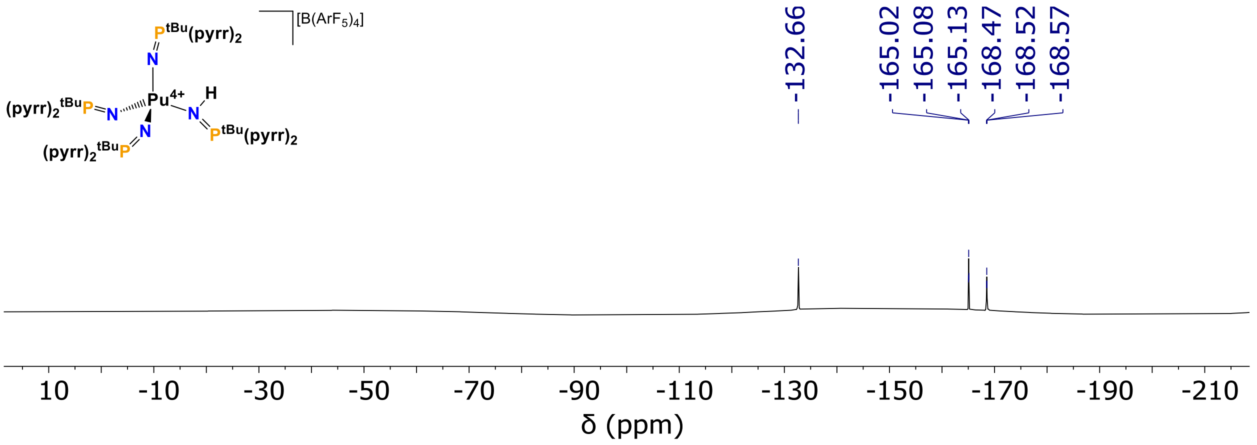

Figure S21. ^{19}F NMR of **3-Pu** in d_8-THF.

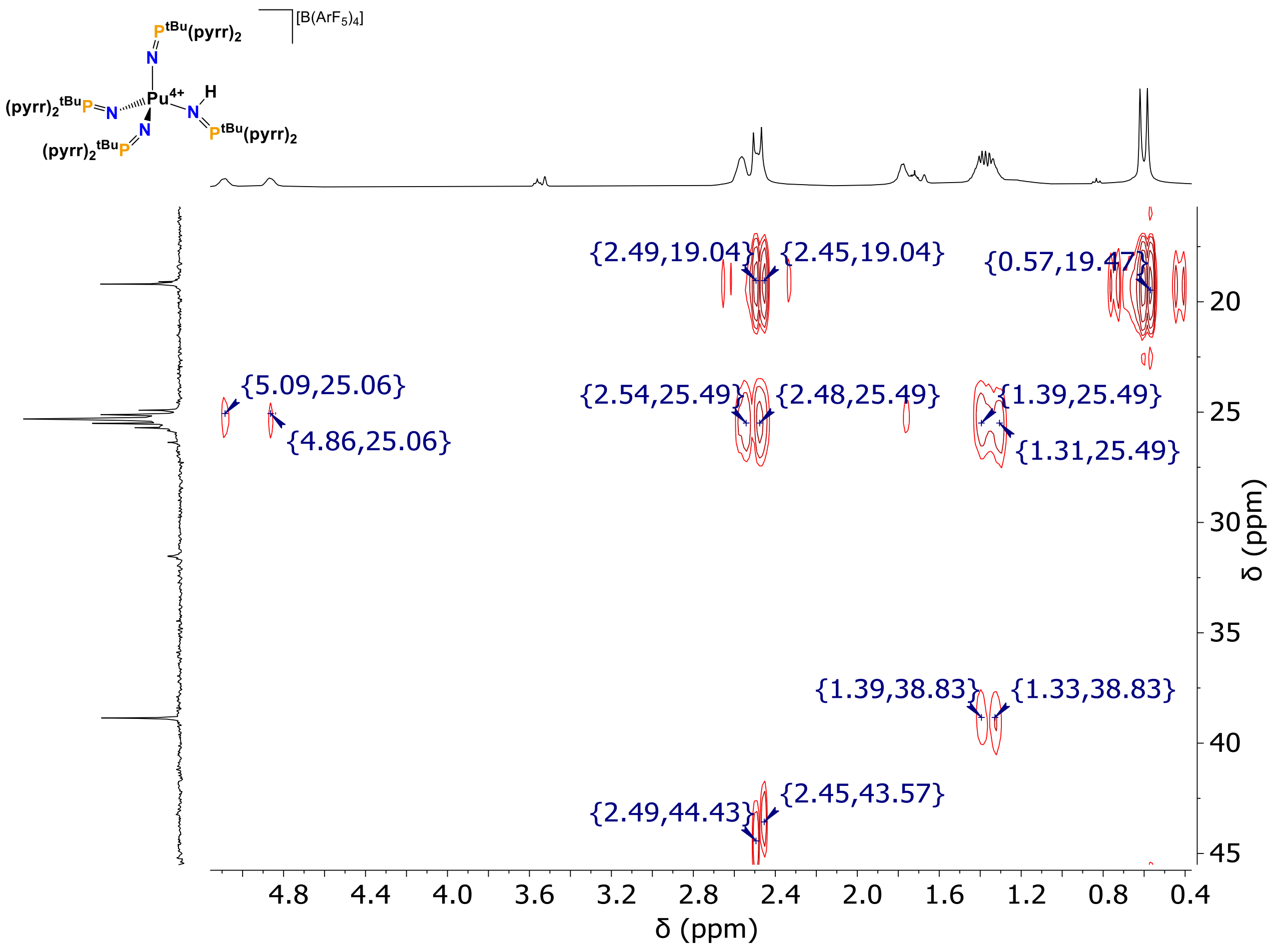

Figure S22. ^{1}H-^{13}C HMBC NMR of **3-Pu** in d_8-THF.

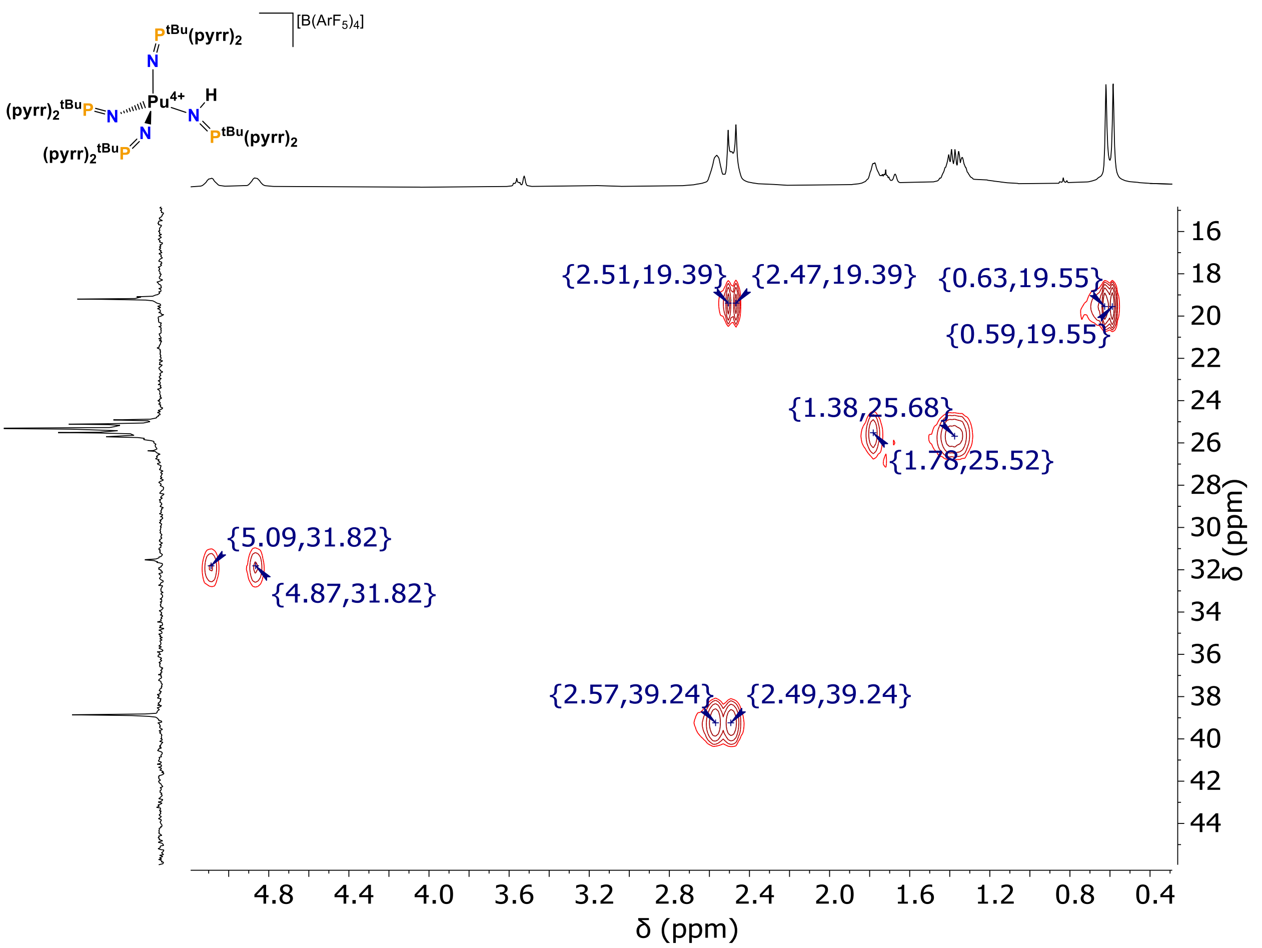

Figure S23. ^{1}H-^{13}C HSQC NMR of **3-Pu** in d$_8$-THF.

Electrochemical Studies

All electrochemical characterization was performed in an inert-atmosphere glovebox using a Pine WaveNow potentiostat and a three-electrode cell. All measurements were recorded at ~20 °C in THF dried and degassed as outlined in general considerations. We have previously optimized and detailed the critical aspects of conducting inert-atmosphere electrochemical studies on transuranic analytes (Np and Pu), as well as outlined the cleaning, preparation, and storage of electrodes, and the preparation, purification, and recovery of electrolyte.[10] The following setup was employed in this work:

The electrochemical cell consists of a borosilicate glass 20-mL scintillation vial and a custom Teflon cap fitted to securely hold a glassy carbon working electrode (WE) with a PCTFE (polychlorotrifluoroethylene) body, polished silver wire pseudo-reference electrode (fritted) (RE), and a platinum wire (straight, 0.5 mm, bare) counter electrode (CE). A working volume of 5 mL of analyte solution was used—this is the minimum volume required to provide the optimal surface area contact of the straight platinum CE. We note that the PCTFE body of the glassy carbon WE is crucial for these complexes, as the PTFE (polyetrafluoroethylene) body of more affordable WEs is incompatible with these complexes under reducing conditions.

Electrolyte solution (0.05 M $TBABPh_4$ ($[N(^nBu)_4][BPh_4]$) in THF) was prepared fresh before experiments using $TBABPh_4$ that had been triply recrystallized (2 times from 10% water in acetone, and once from 70:30 acetone:diethyl ether) and dried on a schlenk line at 100 °C for 24 h.[10] A CV of the electrolyte "blank" was collected prior to experiments (Figure S29) and used to determine the uncompensated solution resistance (R_u, Ω) via a positive feedback experiment (RuPF). The iR compensation value (Ω) is set to 90-95% of the determined R_u.

Plotting and Referencing:

All CVs are plotted according to the IUPAC convention and referenced to the $Fc^{+/0}$ couple via an internal ferrocene and/or decamethylferrocene standard. Unless otherwise noted graphically or in the caption, the scans shown are sweep segments 3 and 4 (out of 6) ("cycle 2"). The first scan segment, which is usually not depicted, is conducted starting at a resting potential 100-300 mV inside of the cycled voltage range. The direction of the scan and the starting point of the scan (or segment) is indicated graphically by arrows adjacent to the voltammogram. Ep values of all redox couples were determined using the first derivative.

Reference values for ferrocene and decamethylferrocene were established by addition of several milligrams of either (or both) compound(s) as an internal standard to the analyte solution after experiment sets were completed, and conducting an additional scan at 200 mV/s. Additionally, before and after each set of experiments, an electrolyte solution containing ferrocene and decamethylferrocene was measured using the same three-electrode cell as a comparison to the internal standard both to ensure that differences in the observed redox potentials were minimal (less than 20 mV), and to confirm that no significant electrode drift (> 20 mV) had occurred. We have established that the standard redox couple of $Cp*_2Fe^{+/0}$ is -0.50 V *vs*. C $p_2Fe^{+/0}$ in 0.05 M $TBABPh_4$ in THF.[10] While both $Cp*_2Fe$ and Cp_2Fe are used in our referencing checks before and after experiments as quality controls, our protocol most commonly uses the potential of the internal $Cp*_2Fe^{+/0}$ standard plus a standard -0.50 V adjustment to report all redox couples against the $Cp_2Fe^{+/0}$ unless otherwise specified.

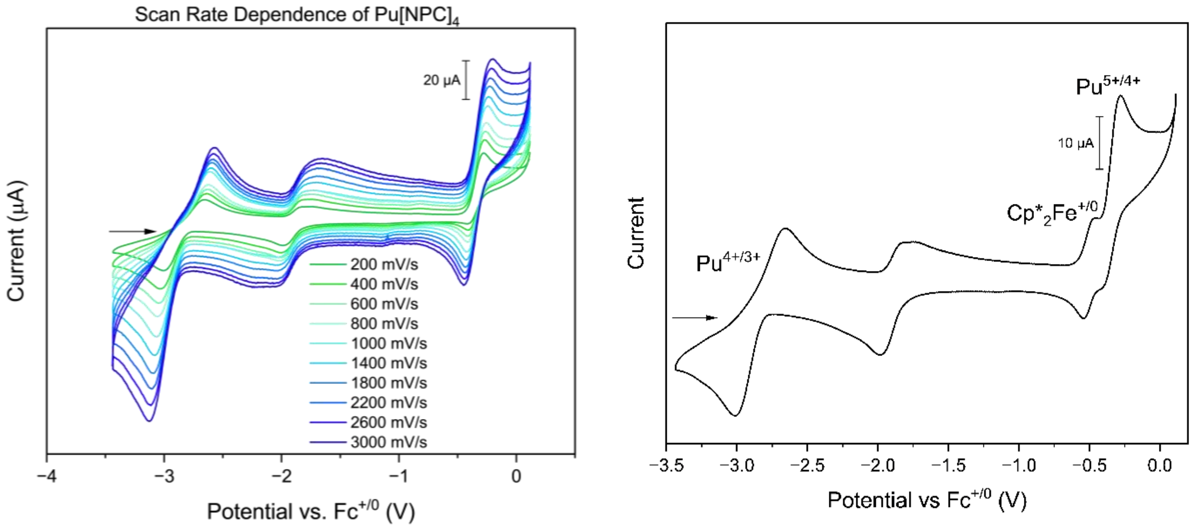

Figure S24. Left) Scan-rate dependence of **1-Pu** (2.85 mM; 0.05 M $[N(^nBu)_4][BPh_4]$ in THF); Right) Full window scan (200 mV/s) of **1-Pu** with added decamethylferrocene. iR compensation = 2,300 Ω.

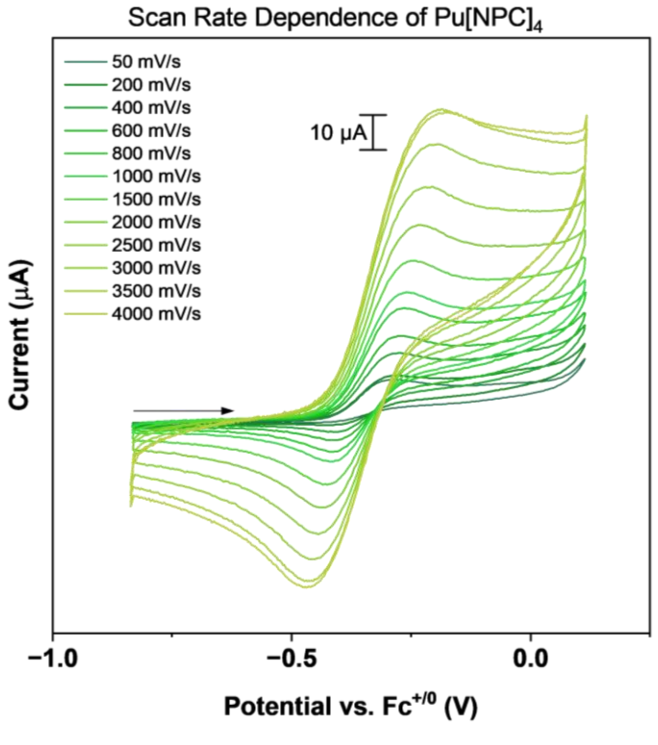

Figure S25. Scan-rate dependence of $Pu^{5+/4+}$ in **1-Pu** (2.85 mM; 0.05 M $[N(^nBu)_4][BPh_4]$ in THF). iR compensation = 2,300 Ω.

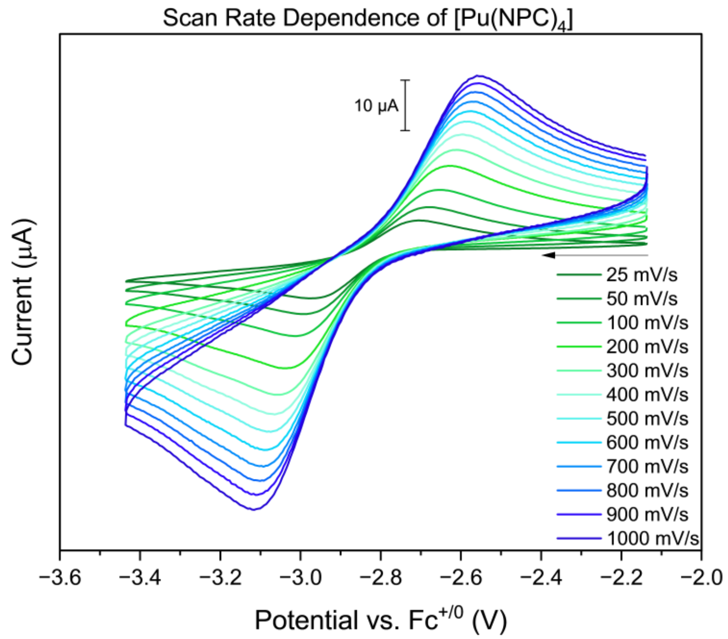

Figure S26 Scan-rate dependence of $Pu^{4+/3+}$ in **1-Pu** (2.85 mM; 0.05 M [N(^{n}Bu)$_4$][BPh$_4$] in THF). iR compensation = 2,300 Ω.

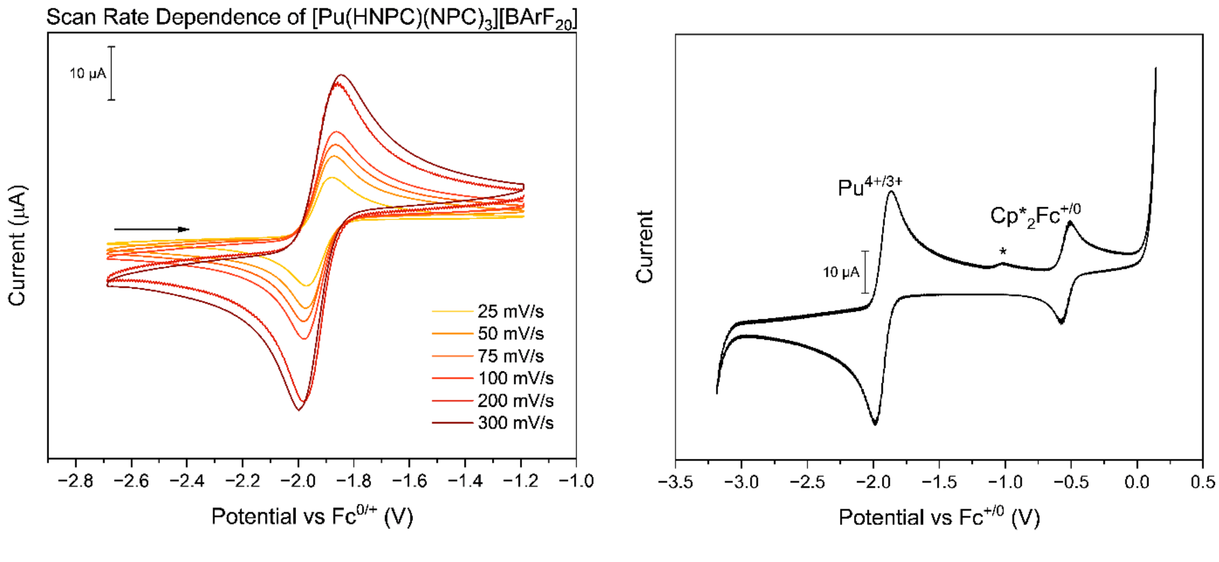

Figure S27. Left) Scan-rate dependence of **3-Pu** (2.25 mM; 0.05 M [N(^{n}Bu)$_4$][BPh$_4$] in THF). Right) Full window scan (200 mV/s) of **3-Pu** with added decamethylferrocene. * Denotes artifact present due to partial stripping of electrolyte solution at the anodic endge of the window. iR compensation = 2,200 Ω.

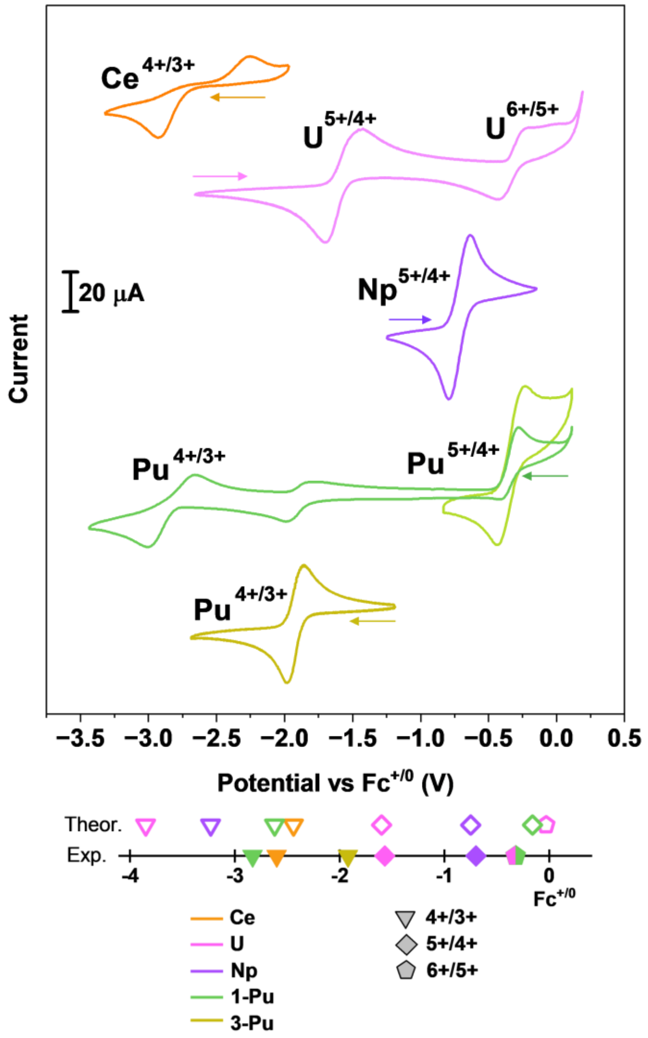

Figure S28. Cyclic voltammograms at 200 mV/s of Ce(NPC)$_4$, U(NPC)$_4$, Np(NPC)$_4$, **1-Pu** (Pu$^{5+/4+}$ wave also shown at 4000 mV/s), and **3-Pu** (~3 mM; 0.05 M [N(^{n}Bu)$_4$][BPh$_4$] in THF), and comparison of experimental (solid) and theoretical (hollow) E$_{1/2}$ values.

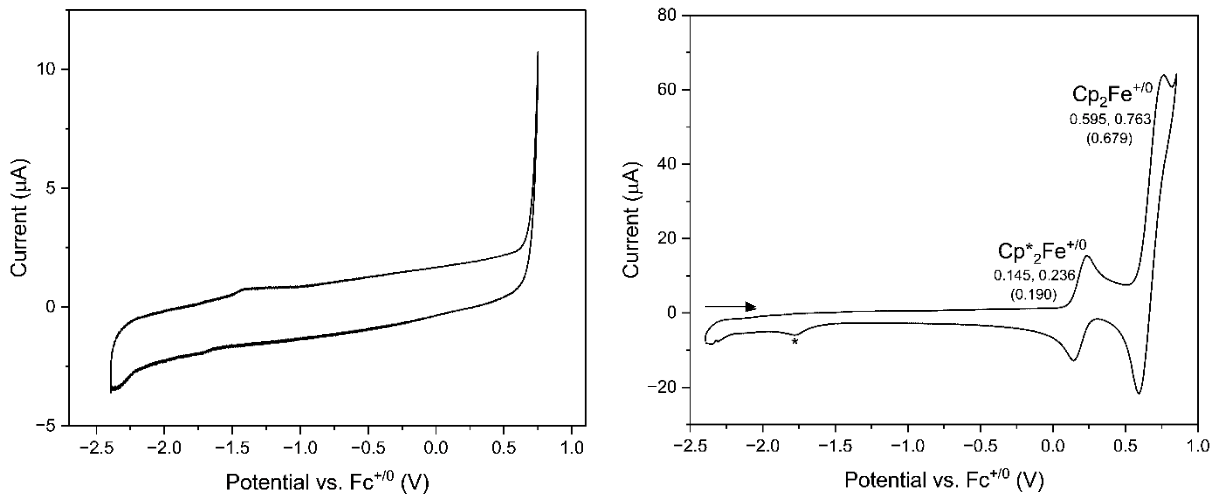

Figure S29. Cyclic voltammograms of blank electrolyte (left) (0.05 M $[N(^nBu)_4][BPh_4]$) in THF, and after addition of Cp^*_2Fe and Cp_2Fe to the solution (right). iR compensation = 2,200 Ω. * Denotes feature arising due to partial stripping of electrolyte solution at the anodic edge of the window.

Kinetic Analysis

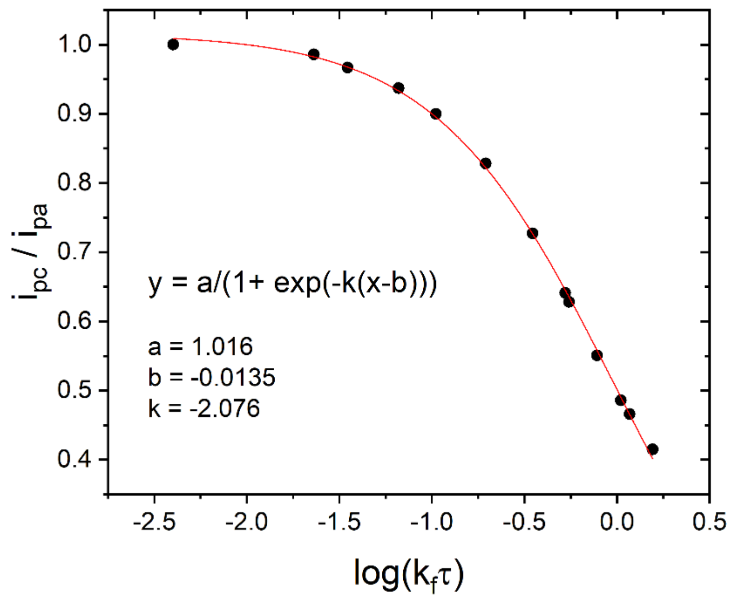

Figure S30. Working curve was fit with a sigmoid function, and the function was used to solve for k_f using the peak current ratios of the $Pu^{5+/4+}$ wave. The sigmoid function was solved for x, and i_{pc}/i_{pa} current values were substituted into the equation for y to solve for log(kft). τ was calculated by dividing the ($E_{1/2}$ -Esp)/SR, which was then used to solve for k_f. This analysis was performed for each scan rate, and the numbers were then averaged to obtain the value of 1.7 ± 0.4 s-1.

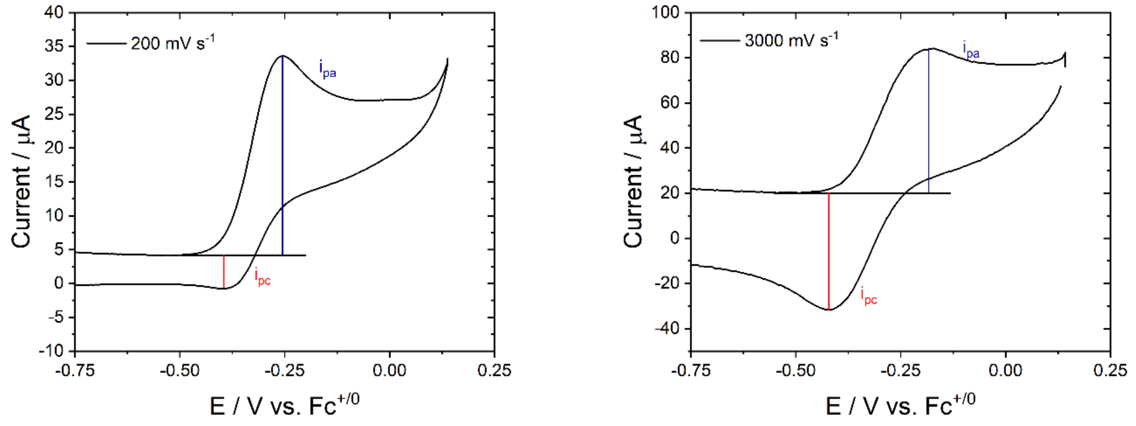

Figure S31. Examples of how i_{pa} and i_{pc} were determined at a) 200 mV s^{-1} and b) 3000 mV s^{-1} demonstrating change in reversibility of the $Pu^{5+/4+}$ wave with scan rate.

Determination of Diffusion Coefficients

The diffusion coefficients for the plutonium complexes were determined using the Randles-Sevcik equation:[11]

$$i_p = 0.4463nFAC\sqrt{nFD/RT} \times \sqrt{v}$$

Here i_p is the peak current, n is number of electrons transferred, F is the Faraday's constant (96485 C mol^{-1}), T is temperature (298 K), R is the universal gas constant (8.314 J K^{-1} mol^{-1}), A is the surface area of the electrode (0.0707 cm^2), C is the concentration of the redox-active species (in mol cm^{-3}), and v is the scan rate (in V s^{-1}). Slopes of the i_p *vs*. $v^{1/2}$ plots in Figures S32-S34 below give average diffusion coefficients for the different plutonium species.

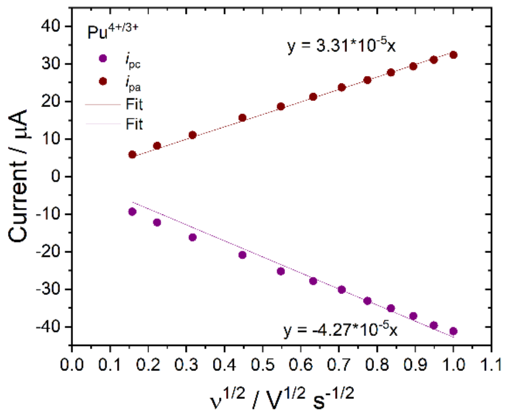

Figure S32. Peak current (corrected for capacitive current) as a function of the square root of scan rate for the $Pu^{4+/3+}$ couple. From the slopes, an average diffusion coefficient of 5.9 × 10^{-7} cm^2/s was calculated.

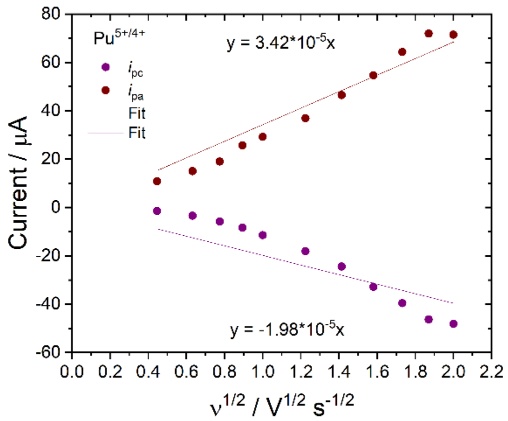

Figure S33. Peak current (corrected for capacitive current) as a function of the square root of scan rate for the $Pu^{5+/4+}$ couple. Only the anodic slope was used to calculate a diffusion coefficient of 7.3×10^{-7} cm^2/s due to the irreversibility of the $Pu^{5+/4+}$ couple.

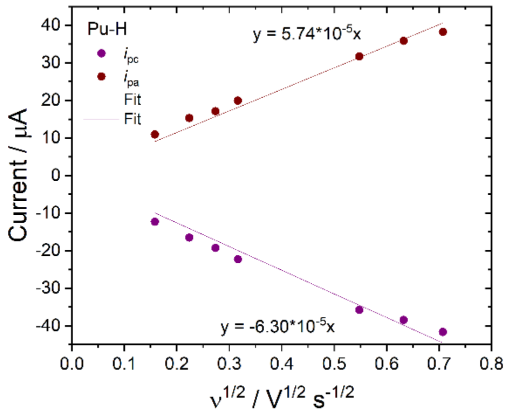

Figure S34. Peak current (corrected for capacitive current) as a function of the square root of scan rate for the **3-Pu** couple. From the slopes, an average diffusion coefficient of 1.8×10^{-6} cm^2/s was calculated.

Determination of Heterogeneous Electron Transfer Rate Constants

The heterogeneous electron transfer rate constants (k_s) for were determined through trumpet plot analysis. An in-depth analysis is described in the experimental section of reference 10.[12]

For **1-Pu**, the k_s values were determined to be 7.67 x 10^{-4} cm/s (Δ = 5.0) for the $Pu^{5+/4+}$ wave, 8.57 x 10^{-5} cm/s (Δ = 7.0) for the $Pu^{4+/3+}$ wave.

For **3-Pu**, the k_s value was determined to be 1.35 x 10^{-3} cm/s (Δ = 5.0) for the $Pu^{4+/3+}$ wave.

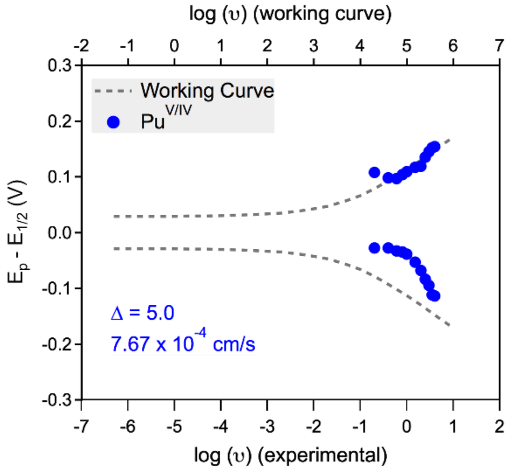

Figure S35. Trumpet plot of the $Pu^{5+/4+}$ wave of **1-Pu** overlaid with the working curve (dashed grey line). The working curve used the parameters D = 1 × 10^{-5} cm^2/s and k_s = 1 cm/s. The offset (Δ) between the data and the x-axis of the best fitting working curve is used to determine k_s value of 7.67 x 10^{-4} cm/s (Δ = 5.0) for the $Pu^{5+/4+}$ wave. Given the error associated with the overlap between the anodic $Pu^{5+/4+}$ trumpet plot and the working curve, the k_s value was only calculated from the cathodic portion of the dataset.

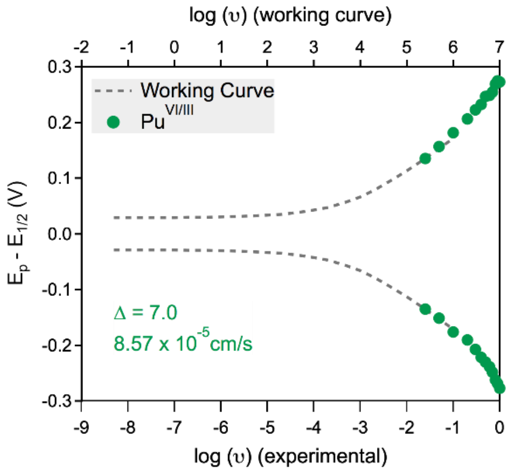

Figure S36. Trumpet plot of the $Pu^{4+/3+}$ wave of **1-Pu** overlaid with the working curve (dashed grey line). The working curve used the parameters D = 1 × 10^{-5} cm^2/s and k_s = 1 cm/s. The offset (Δ) between the data and the x-axis of the best fitting working curve is used to determine k_s value of 8.57 x 10^{-5} cm/s (Δ = 7.0) for the $Pu^{4+/3+}$ wave.

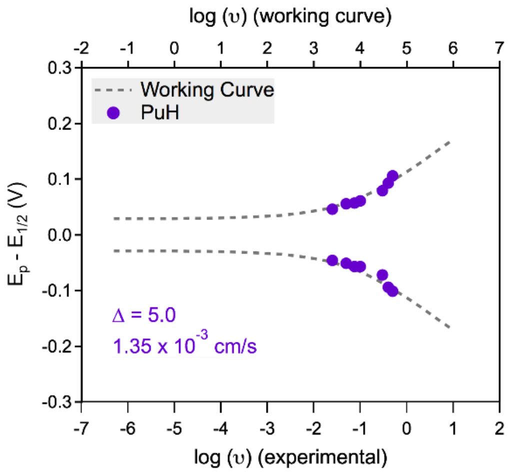

Figure S37. Trumpet plot of the $Pu^{4+/3+}$ wave of **3-Pu** wave overlaid with the working curve (dashed grey line). The working curve used the parameters D = 1 × 10^{-5} cm^2/s and k_s = 1 cm/s. The offset (Δ) between the data and the x-axis of the best fitting working curve is used to determine k_s value of 1.35 x 10^{-3} cm/s (Δ = 5.0) for the $Pu^{4+/3+}$ wave.

Digital Simulations

All simulations were performed using DigiElch 8 from ElchSoft Electrochemical Siulation Software.

Table S1. Charge transfer reactions used to simulate voltammograms of **1-Pu**

Assignment	Charge transfer reaction	$E°$ (V)	α/λ (eV)	k_s (cm s^{-1})
E_1	$[Pu^{V}] + e^{-} \rightleftharpoons [Pu^{IV}]$	–0.34*	0.5	7.7 x 10^{-4}
E_2	$[PuH^{Ox}]^{+} + e^{-} \rightleftharpoons [PuH^{Red}]$	–1.85*	0.5	1.4 x 10^{-3}
E_4	$[Pu^{IV}] + e^{-} \rightleftharpoons [Pu^{III}]$	–2.81*	0.5	8.6 x 10^{-5}

* indicates experimentally determined values.

Table S2. Chemical reactions used to simulate voltammograms of **1-Pu**

Assignment	Chemical reaction	K_{eq}	k_f (s^{-1})*	k_b (s^{-1})
C_1	$[Pu^{V}] \rightarrow [PuH^{Ox}]$	-	varied	-

*indicates experimentally determined values.

Table S3. Chemical species used to simulate voltammograms of **1-Pu**

Chemical species	D (cm^2 s^{-1})	C_{anal} (M)^a	C_{init} (M)^b
$[Pu^{V}]$	5.9 x 10^{-7}*	0	0
$[Pu^{IV}]$	7.3 x 10^{-7}*	2.3 x 10^{-3}	0
$[Pu^{III}]$	7.3 x 10^{-7}*	0	0
$[PuH^{Red}]$	1.8 x 10^{-6}*	0	0
$[PuH^{Ox}]^{+}$	1.8 x 10^{-6}*	0	0

$^a C_{anal}$ = initial concentration of species prior to any chemical or charge transfer reactions. $^b C_{init}$ = concentration of species accounting for any chemical reactions prior to charge transfer reactions. C_{init} is automatically calculated by simulation software. *indicates experimentally determined values.

Table S4. Chemical reactions used to simulate voltammograms of **1-Pu** for simulations represented in Figure S38.

Assignment	Chemical reaction	K_{eq}	k_f (s^{-1})*	k_b (s^{-1})
C_1	$[Pu^V] \rightarrow [PuH^{Ox}]$	-	1.7	-

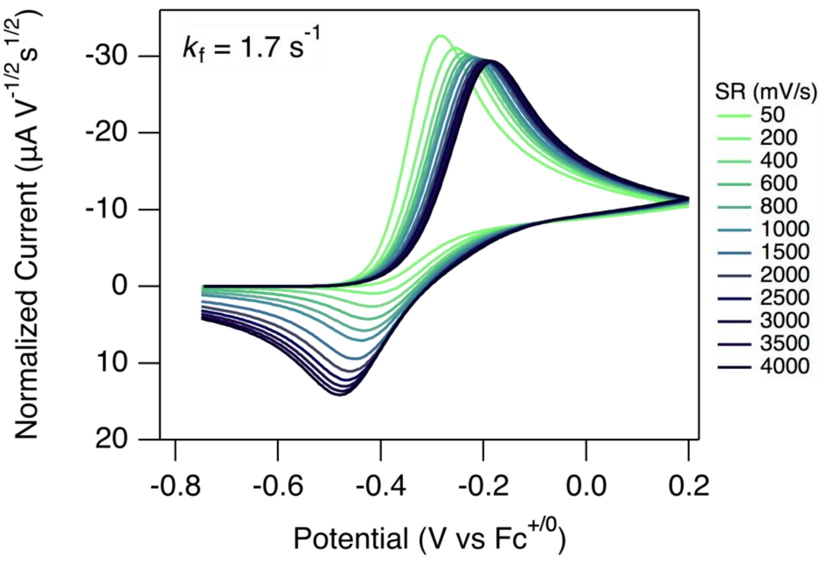

Figure S38. Simulated cyclic voltammograms of 2.3 mM **1-Pu** at rates range from 50 to 4000 mV/s using parameters detailed in Tables S1, S3, and S4. Current values have been normalized to scan rate.

Table S5. Chemical reactions used to simulate voltammograms of **1-Pu** for simulations represented in Figure S39.

**Assignment**	**Chemical reaction**	K_{eq}	k_f (s^{-1})*	k_b (s^{-1})
C_1	$[Pu^{V}] \rightarrow [PuH^{Ox}]$	-	30	-

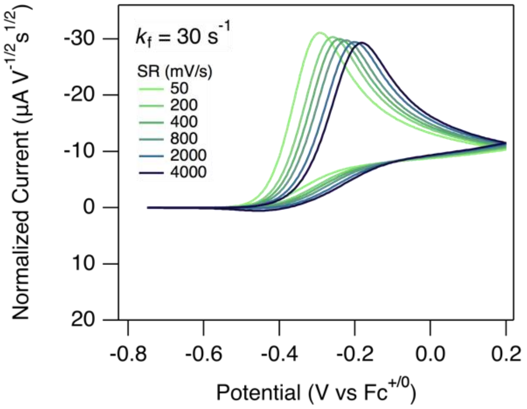

Figure S39. Simulated cyclic voltammograms of 2.3 mM **1-Pu** at rates range from 50 to 4000 mV/s using parameters detailed in Tables S1, S3, and S5. Current values have been normalized to scan rate.

Table S6. Chemical reactions used to simulate voltammograms of **1-Pu** for simulations represented in Figure S40.

Assignment	Chemical reaction	K_{eq}	k_f (s^{-1})*	k_b (s^{-1})
C_1	$[Pu^V] \rightarrow [PuH^{Ox}]$	-	0.3	-

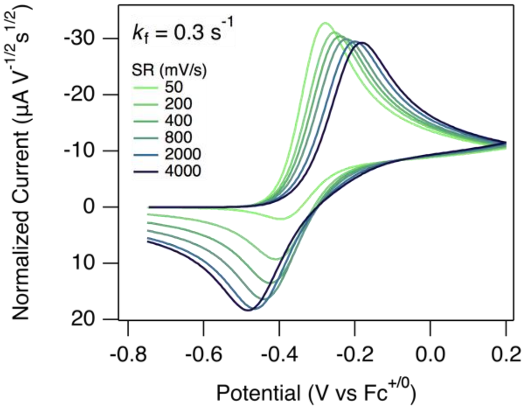

Figure S40. Simulated cyclic voltammograms of 2.3 mM **1-Pu** at rates range from 50 to 4000 mV/s using parameters detailed in Tables S1, S3, and S6. Current values have been normalized to scan rate.

Single-Crystal X-ray Diffraction

General collection details: Crystal mounting was conducted following the methods outlined previously[3] for transuranic crystals, then mounted on a dual source (Mo/Cu) Bruker D8 VENTURE four circle diffractometer with a microfocus sealed X-ray tube using a mirror optics as monochromator and a Bruker PHOTON II detector. The diffractometer was equipped with an Oxford Cryostream 800 low temperature device and used MoKα (λ = 0.71073 Å). Crystals were cooled and kept at T = 100(2) K during data collections. All data were integrated with SAINT and a multi-scan absorption correction using SADABS was applied.[13, 14] The structures were solved with the ShelXT structure solution program using the Intrinsic Phasing solution method and refined by full-matrix least squares methods against F^2 using SHELXL-2014[15] and by using Olex2 1.5-alpha[16] as the graphical interface. All non-hydrogen atoms were refined with anisotropic displacement parameters. Disordered moieties were refined using bond lengths restraints and displacement parameter restraints. Hydrogen atoms were modelled using a riding model for all but the unique atom on **3-Pu**. This atom was instead located on the electron difference map crystallographically and refined as a proton localized on N1D within an applied bond length parameter. When refined freely the H-atom remained localized on the same ligand but the model would not converge. Crystallographic data for the structures reported in this paper have been deposited with the Cambridge Crystallographic Data Centre (2347339 -234741).

The data quality across all three structures necessitated the use of heavy constraints and restraints (including RIGU, SIMU, DELU, and ISOR), to obtain convergent solutions that were chemically sensible. Crystals of **1-Pu** and **3-Pu** crystalized isomorphous with their U and Np analogues allowing an existing model for the disorder present to be used, and the disorder to be fully modeled. These have been described in detail previously (See: SI of [1, 3, 17].) Unfortunately, this strategy could not be used on **2-Pu** as it was not isomorphous with our previous Ce and Np models.

The crystal of **2-Pu** is twinned, with at least three separate components. Trying to model the system with these included increases the R1 by ~1.5% and the wR2 by ~8%, indicating a worse model for this refinement. Several different models were attempted for this dataset, the one presented here was chosen for having the best overall statistics for R1, wR2, GooF, etc. There was additional disorder present in **2-Pu** that was not modeled in the chosen solution as the system would not converge, or statistically worsened the model substantially. These models include the modeling for the groups off P4 – here two of the three ligand arms are modeled as a 50:50 exchange of a *t*-Bu group and pyrrolidine, while the third is just modeled as a pyrrolidine at 100% occupancy. An example of additional modeling of this disorder included trying to split this “100 %” pyrrolidine. However, for this model the R1, wR2, GooF all increased substantially and the model did not converge even when treating these atoms isotropically or using additional restraints/constraints. The multiple points of rotation possible in this ligand caused a smearing of the electron density that impedes further disorder modeling in this structure.

Table S7. SC-XRD collection and refinement data.

Compound	1-Pu	2-Pu	3-Pu
Empirical Formula	$C_{48}H_{100}N_{12}PuP_4$	$C_{66}H_{134.5}KN_{14}O_6PuP_4$	$C_{72}H_{101}BF_{20}N_{12}PuP_4$
Formula Weight	1211.27	1625.35	1891.33
Temperature (K)	100(2)	100(2)	100(2)
Crystal System	Monoclinic	Orthorhombic	Triclinic
Space Group	$P2_1/n$	Pbca	P-1
Resolution (Å)	0.82	0.83	0.85
I/σ	16.4	22.1	22.3
a / Å	24.357(3)	19.660(6)	13.2078(7)
b / Å	23.219(2)	26.102(8)	15.5727(8)
c / Å	40.756(4)	30.904(13)	20.2966(10)
α /∘	90	90	75.464(2)
β /∘	97.295(3)	90	84.271(2)
γ /∘	90	90	86.348(2)
Volume/$Å^3$	22863(4)	15859(10)	4017.6(4)
Z	16	8	2
Z'	4	1	1
ρ (g/cm^3)	1.408	1.361	1.563
μ (mm^{-1})	1.308	1.019	0.996
F(000)	10016.0	6796.0	1912.0
Crystal Size /mm^3	0.3277 x 0.215 x 0.096	0.572 x 0.327 x 0.262	0.361 x 0.281 x 0.277
Radiation	MoKα (λ=0.71073)	MoKα (λ=0.71073)	MoKα (λ=0.71073)
Color/Habit	Green/block	Reddish yellow/block	Brownish red/block
2θ range for data collection(∘)	3.71 to 51.364	3.698 to 50.7	3.882 to 49.426
Index Ranges	-26 ≤ h ≤ 29, -28 ≤ k ≤ 28, -49 ≤ l ≤ 49	-23 ≤ h ≤ 21, -29 ≤ k ≤ 31, -37 ≤ l ≤ 35	-15 ≤ h ≤ 15, -18 ≤ k ≤ 18, -23 ≤ l ≤ 23
Reflections Collected	332954	216518	142104
Data/Restraints/ Parameters	43255/11824/2510	14501/2284/925	13708/1081/1101
Goodness-of-Fit on F^2	1.050	1.076	1.042
Final R Indexes [I>=2σ (I)]	R_1 = 0.1261, wR_2 = 0.3148	R_1 = 0.0994, wR_2 = 0.2153	R_1 = 0.0464, wR_2 = 0.1201
Final R Indexes [all data]	R_1 = 0.1767, wR_2 = 0.3445	R_1 = 0.1237, wR_2 = 0.2302	R_1 = 0.0538, wR_2 = 0.1260
Largest Diff. Peak/Hole/(e $Å^3$)	5.71/-4.44	2.94/-1.79	1.80/-1.64
Completeness to 2θ	99.6	99.8	100
CCDC Number	2347340	2347339	2347341

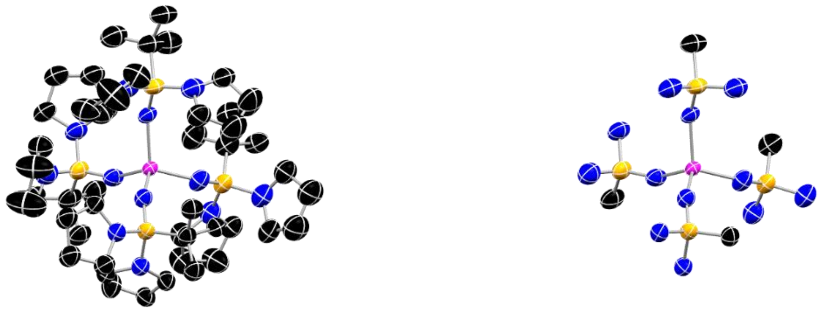

Fig. S41. Full and truncated molecular structure of **1-Pu**, as determinde by SC-XRD. Thermal ellipsoids (pink, Pu; blue, N; black, C; orange, P) shown at 50% and hydrogen atoms removed for clarity.

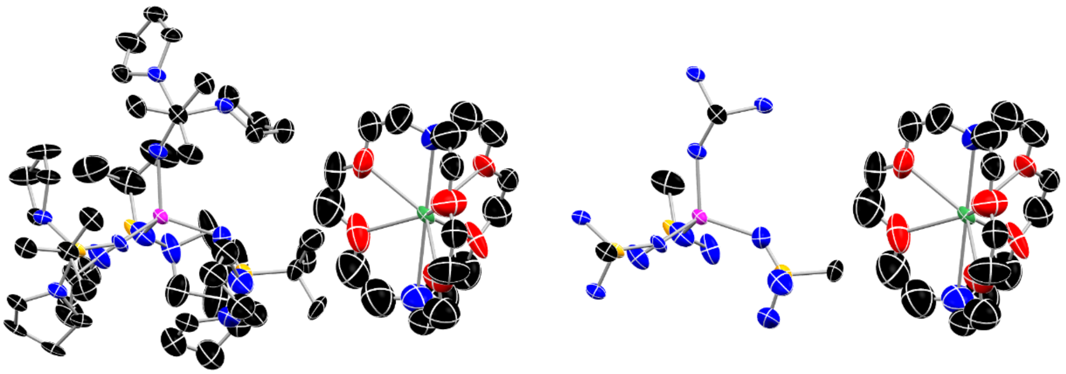

Fig. S42. Full and truncated molecular structure of **2-Pu**, as determinde by SC-XRD. Thermal ellipsoids (pink, Pu; green, K; blue, N; black, C; orange, P) shown at 50% and hydrogen atoms removed for clarity.

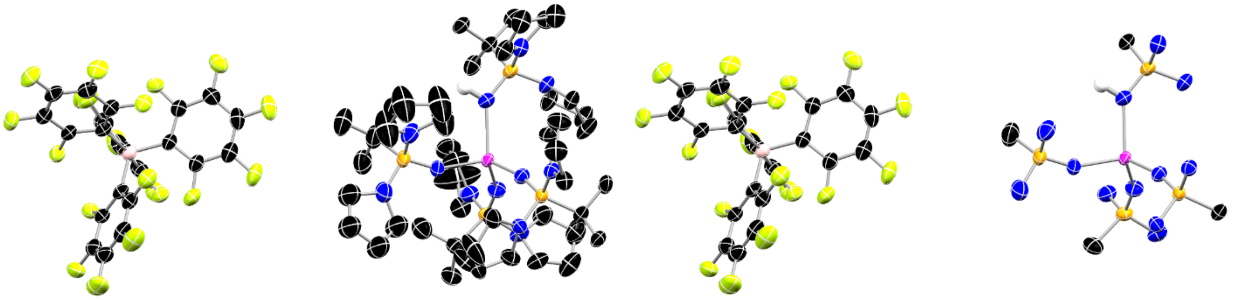

Fig. S43. Full and truncated molecular structure of **3-Pu**, as determined by SC-XRD. Thermal ellipsoids (pink, Pu; light green, F; light pink, B; blue, N; black, C; orange, P; white, H) shown at 50% and most hydrogen atoms removed for clarity.

Table S8. Selected structural metrics of $M^{n+}(NPC)_4$ complexes (n=3-5).

Complex	Average Distance (Å)		τ_4	Average Angle (°)	
	M–N*	P=N		N–M–N	M–N=P
$[Ce^{3+}(NPC)_4]$[K-2.2.2-C] [A]	2.315(5)	1.540(18)	0.94(4)	109(3)	147(3)
$[Np^{3+}(NPC)_4]$[K-2.2.2-C] [A]	2.291(5)	1.530(4)	0.95(3)	109(5)	146(1)
$[Pu^{3+}(NPC)_4]$[K-2.2.2-C] (**2-Pu**)	2.257(10)	1.537(15)	0.99(4)	109(1)	147(8)
$Ce^{4+}(NPC)_4$ [A]	2.160(6)	1.539(1)	0.94(4)	109(5)	158(6)
$U^{4+}(NPC)_4$ [B]	2.167(7)	1.538(2)	0.95(3)	110(4)	159(8)
$Np^{4+}(NPC)_4$ [A]	2.156(7)	1.533(2)	0.95(3)	109(5)	157(6)
$Pu^{4+}(NPC)_4$ (**1-Pu**)	2.141(11)	1.540(13)	0.97(2)	109(5)	157(6)
$[Np^{4+}(NPC)_3(HNPC)][B(ArF_5)_4]$ [C]	2.133(18)<br>2.390(7)**	1.557(4)<br>1.582(7)**	0.93(3)	109(5)	156(7)<br>140.2(3)**
$[Pu^{4+}(NPC)_3(HNPC)][B(ArF_5)_4]$ (**3-Pu**)	2.118(16)<br>2.378(7)**	1.557(4)<br>1.583(7)**	0.92(2)	109(5)	156(7)<br>139.7(3)**
$[U^{5+}(NPC)_4][B(ArF_5)_4]$ [A]	2.101(5)	1.565(12)	0.95(2)	109(3)	168(6)
$[Np^{5+}(NPC)_4][B(ArF_5)_4]$ [B]	2.085(16)	1.569(7)	0.96(2)	109(2)	166(4)

* Numbers given in parentheses are average esd values, as these are average distances from 4–20 independent high-precision measurements (due to symmetry, Z, and disorder) which have a wide distribution of M–N distances. ** Shows the metric pertaining to the one unique protonated ligand. [A] previously published.[3] [B] previously published.[1] [C] previously published.[17]

DFT Calculations

$Pu^{n+}(NPC)_4$ (n = 3-5) and $Pu^{n+}(NPC)_3(HNPC)$ (n = 3-4) were fully optimized in the gas phase without any constraints. Starting geometries were based on the XRD structures, when available. The starting geometries for $Pu^{5+}(NPC)_4$ and $Pu^{3+}(NPC)_3(HNPC)$ were based on the optimized structure of the closest available oxidation state. All optimizations were carried out using the PBE0[18] hybrid DFT functional as implemented in the Gaussian16 software package (version A.03).[19] The ECP60MWB[20] small core quasi-relativistic pseudopotential and ECP60MWB_ANO[21] basis sets were used to describe Pu in all complexes. All remaining atoms were described with the all-electron Pople basis set 6-311G(d).[22] Harmonic frequency calculations were performed to confirm that the optimized structures were stationary points on the potential energy surface. The wavefunctions of the complexes were checked for stability to ensure that the calculations converged to their ground electronic states. In all complexes spin contamination was obtained from single point calculations using the optimized geometries and HF method with the same basis set used for optimizations. Spin contamination was found to be less than 0.1% with $\langle S^2 \rangle$ values being close to the corresponding values of the considered spin states, *i.e.,* quintet for **1-Pu** and **3-Pu**; quartet for [**1-Pu**]$^+$; and sextet for **2-Pu** and $Pu^{3+}(NPC)_3(HNPC)$. The optimized geometries and experimental XRD crystal structures show deviations less than 1.7% for Pu–N bonds and 1.0% for P=N bonds (Tables S9-S10), providing confidence in the computational model.

Electrochemical potentials were calculated using the single-point HF energies of the oxidized and reduced species and referenced to a calculated $Fc^{+/0}$ couple (-4.75 V) employing implicit solvent modeling in THF. The potentials were calculated using an isodesmic reaction with a $Fc^{+/0}$ couple to minimize the errors. Vertical detachment energy (VDE), adiabatic detachment energy (ADE), vertical electron affinity (VEA), and adiabatic electron affinity (AEA) were calculated to assess the amount of the structural reorganization required for the oxidation (ΔDE= VDE-ADE) and reduction (ΔEA=VEA-AEA) reactions (Table S18). VDE was computed as the energy difference between the original complex and its oxidized counterpart, which has the geometry of the original complex. ADE was computed as the energy difference between the original complex and its oxidized counterpart, the geometry of which was fully optimized. VEA was computed as the energy difference between the original complex and its reduced counterpart, which has the geometry of the original complex. AEA was computed as the energy difference between the original complex and its reduced counterpart, the geometry of which was fully optimized.

The BDFE of the N–H bond in **3-Pu** was calculated using the Bordwell equation, as described previously for the analogous Np^{5+} complex.[15] The TEMPO/H system was used as the experimental reference for the dissociation of a single H atom, which has been used before to describe BDFE in transition metal complexes.[23] Since spin-orbit coupling (SOC) plays an increasingly important role as the atomic number increases due to relativistic effects on heavy atoms,[24] calculations for the BDFE were carried out using the fourth order Douglas-Kroll-Hess (DKH4) relativistic Hamiltonian including spin orbit coupling as implemented in Gaussian 16.[25-28] This method has been used in the past to calculate the effects of SOC in actinide systems.[29-31] The PBE0 functional was used along with the all-electron basis set Jorge-TZP-DKH for all atoms.[32, 33] This basis set has been used to accurately calculate dissociation energies in AnO

complexes in the past.[33] To compare only the effects of SOC, calculations were also carried out using the second order Douglas-Kroll-Hess (DKH2) relativistic Hamiltonian. Including relativistic effects or SOC is not expected to significantly impact the geometries of the structures, so we elected to use thermal and zero-point energy corrections from the gas phase optimized structures using the ECP60MWB small core quasi-relativistic pseudopotential and ECP60MWB_ANO basis set with the PBE0 functional. An estimate of the BDFE using DKH2 and DKH4 calculated single point energies was obtained with the thermal and zero-point energy corrections from the non-relativistic gas phase calculations added to it.

The electron localization methods of Adaptive Natural Density Partitioning (AdNDP),[34] using Multiwfn software,[35] and Natural Bond Orbital (NBO) analysis, using the NBO7 code,[36, 37] were implemented to obtain a more chemically intuitive picture of the bonding interactions. By applying the AdNDP algorithm, the total electron density was partitioned transforming the delocalized molecular orbitals into more localized bonding elements, where possible. Unpaired electrons, lone pairs of electrons, or traditional Lewis bonds can be recovered via AdNDP analysis as one-center one-electron (1c-1e), one-center two-electron (1c-2e), and two-center two-electron (2c-2e) interactions, respectively. AdNDP analysis also allows for the assignment of multicenter (*n*c-2e) bonds where *n* is equal to the number of atoms specified by the user. The occupation number (ON) describes the number of electrons occupying a particular identified localized state. Previously, AdNDP was shown to be insensitive to the level of theory used.[38-40] Specifically, it was found that as long as the basis set is large enough to sufficiently describe electronic structure, AdNDP showed no qualitative dependency on the basis set.

QTAIM analysis[41] was used as an alternative approach to study the covalency of the bonds in these complexes. QTAIM uses the topology of the electron density to provide a rigorous definition for an atom in a molecule and to interpret chemical bonding interactions according to stationary points of the electron density and the gradient of the paths that originate and terminate at these points, called bond paths. The properties of the point with the lowest electron density along a bond path, the bond critical point (BCP), provides a universal description of bonding,[42] which characterize a bond according to the electron density ρ, its Laplacian $\nabla^2\rho$, and the total energy density H(r) at these BCPs.[43] QTAIM analysis calculations were performed on the optimized geometries of all complexes by using Multiwfn software.[35]

Single-point energy calculations in THF were performed using the self-consistent reaction field approach based on the integral equation formalism of the polarized continuum model (PCM).[44-46] MO energy level diagrams are drawn based on the single-point energy calculations in THF using the fully optimized geometries at the employed DFT level. Chemissian 4.67[47] was used to plot the MO diagrams. Chemcraft 1.8 (build 610b)[48] was used for the visualization of the NBO/AdNDP results.

Table S9. Optimized bond lengths (Å) and angles (°) for $An^{n+}(NPC)_4$. Experimental values, when available, are shown in brackets. For **[1-Pu]**$^{L+}$ the bond metrics for the oxidized ligand are shown after the average of the three neutral ligands.

	**2-Pu**	**1-Pu**	**[1-Pu]**$^{M+}$	**[1-Pu]**$^{L+}$
An–N	2.295<br>[2.257(10)]	2.160<br>[2.141(11)]	2.092	2.107/2.402
$P{=}N_{im}$	1.538<br>[1.537(15)]	1.555<br>[1.540(13)]	1.585	1.573/1.600
An–N=P	151.2<br>[147(8)]	173.8<br>[157(6)]	175.9	172.5/166.6
τ_4	0.977<br>[0.99(4)]	0.989<br>[0.97(2)]	0.968	0.982

Table S10. Optimized bond lengths (Å) and angles (°) for $An^{n+}(NPC)_3(HNPC)$. Experimental values, when available, are shown in brackets.

	$Pu^{3+}(NPC)_3(HNPC)$	**3-Pu**
An–N	2.239	2.109 [2.118(16)]
An–N*	2.506	2.435 [2.378(7)]
$P{-}N_{im}$	1.546	1.574 [1.557(4)]
P–N*	1.592	1.609 [1.583(7)]
An–N=P	161.6	169.4 [156(7)]
An–N*=P	136.1	140.7 [139.7(3)]
τ_4	0.93	0.92 [0.92(2)]

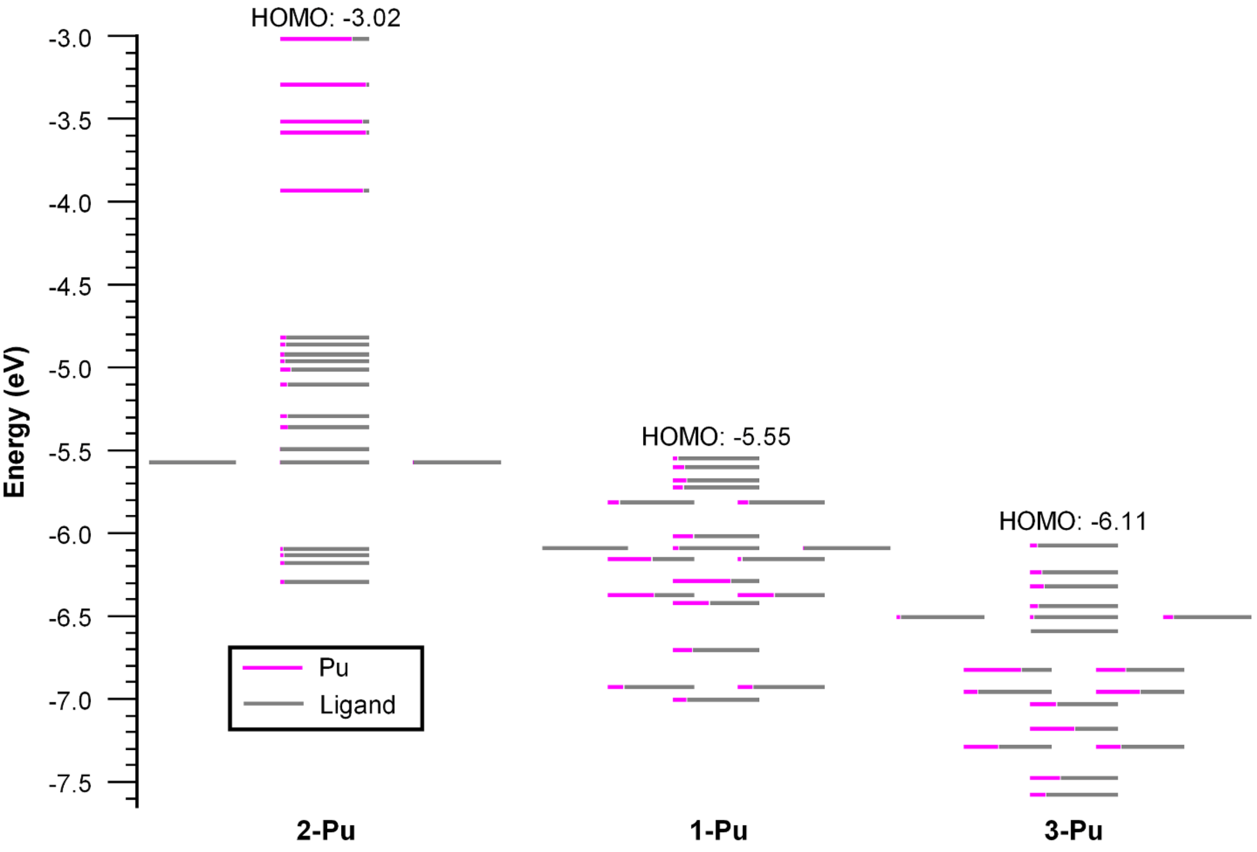

Figure S44. α-MO energy diagrams for the selected occupied frontier orbitals of **1-Pu**, **2-Pu**, and **3-Pu**. The magenta fraction of the MO lines represents the percentage of Pu AOs, and the gray lines are the ligand fraction. Degeneracy is set to 0.035 eV for visualization purposes. Combined α + β MO diagrams are given in Figures S46-S49, with the AO contributions listed in Tables S11-S12.

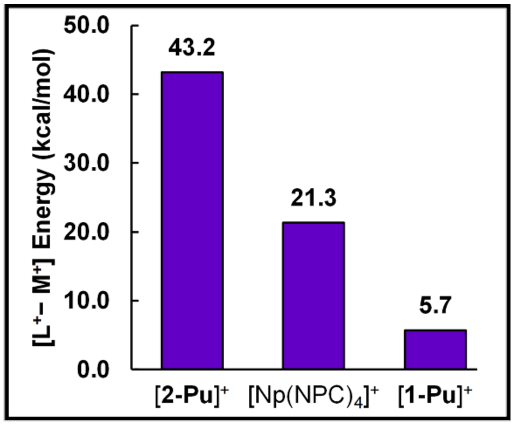

Figure S45. The energy of ligand-oxidized species relative to the energy of the metal-oxidized species in THF, with zero-point energy and thermal corrections from the corresponding gas-phase calculations.

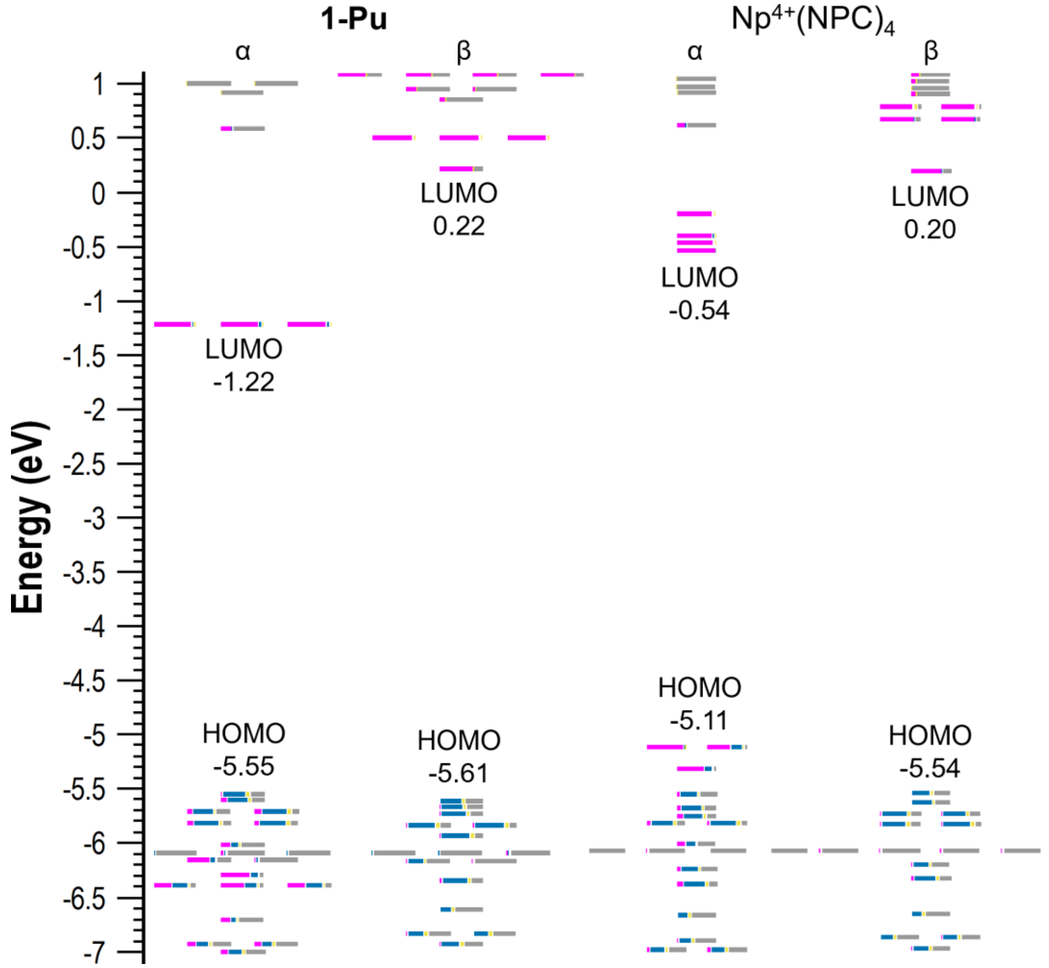

Figure S46. Combined α + β MO diagrams for the optimized [$An^{4+}(NPC)_4$] complexes. AO contributions in the MOs are shown in different colors; An is pink, N is blue, P is yellow, and all others are shown in gray here and elsewhere in the SI. Degeneracy is set to 0.05 eV for visualization purposes.

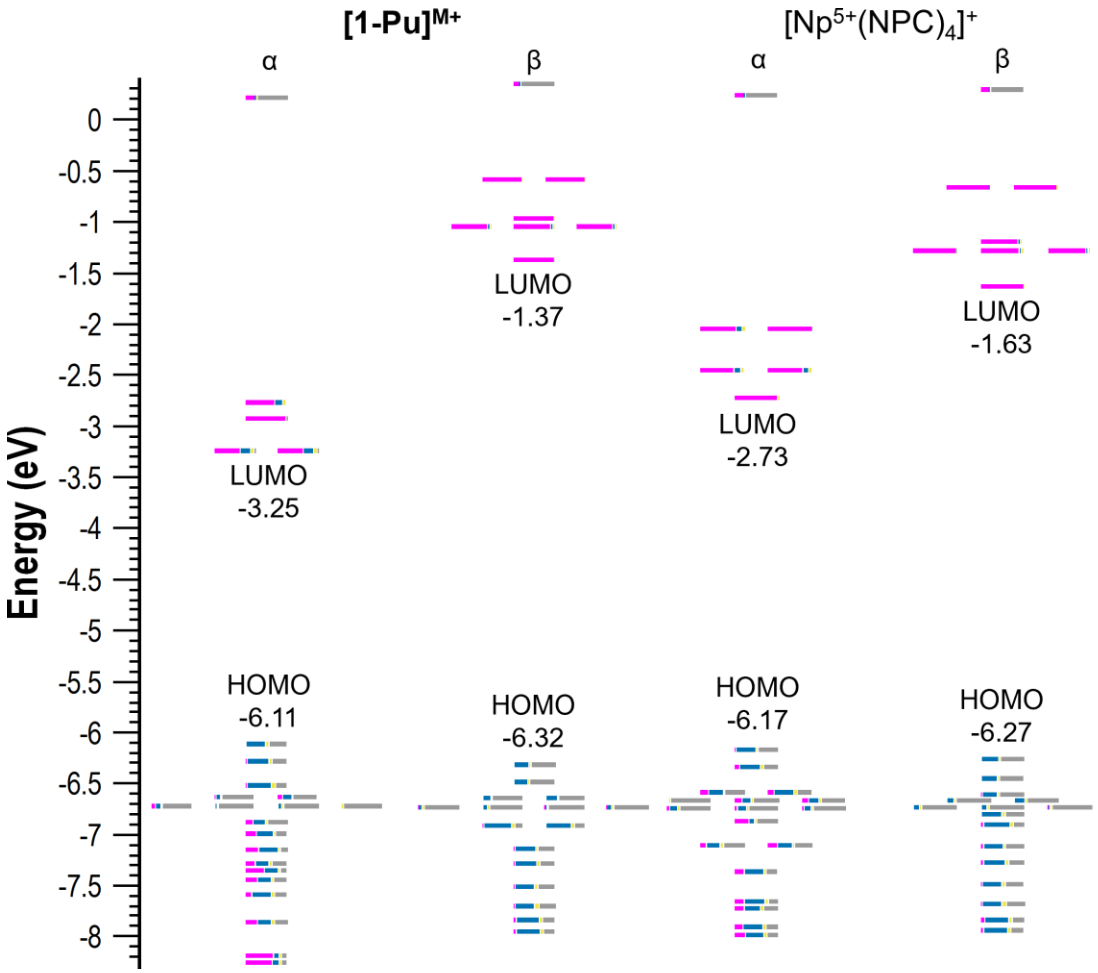

Figure S47. Combined α + β MO diagrams for the optimized $[An^{5+}(NPC)_4]^+$ complexes.

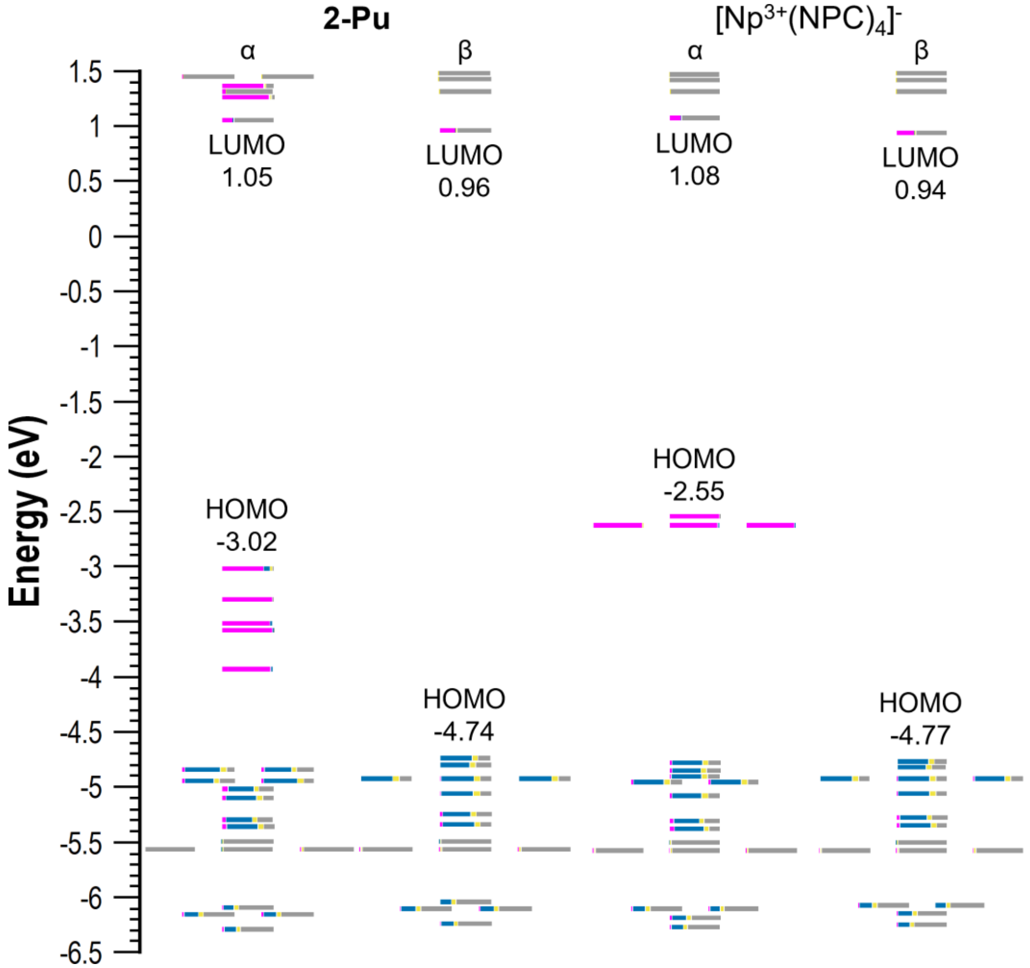

Figure S48. Combined α + β MO diagrams for the optimized $[An^{3+}(NPC)_4]^-$ complexes.

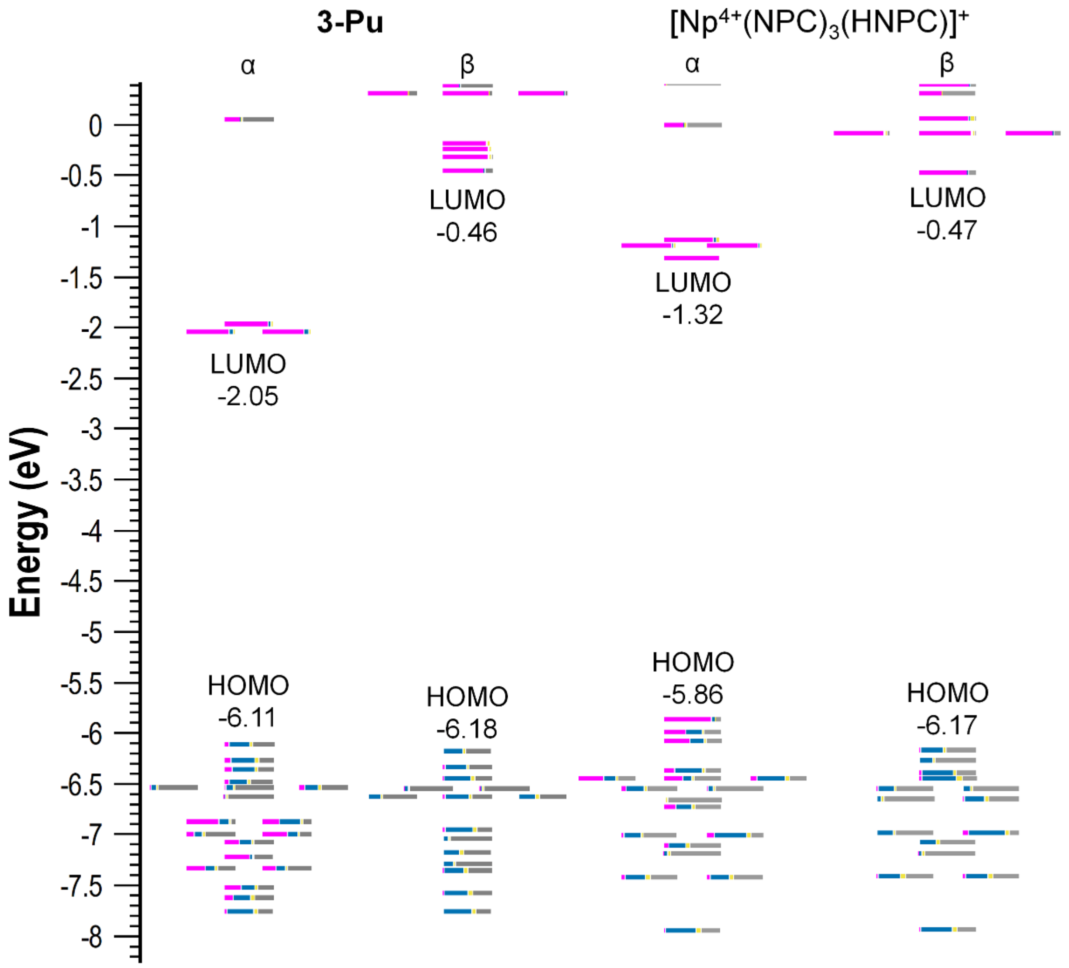

Figure S49. Combined α + β MO diagrams for the optimized $[An^{4+}(NPC)_3(HNPC)]^+$ complexes.

Table S11. Top 20 occupied orbitals of $[An^{n+}(NPC)_4]$ with the contributions of the An (%) and 7*s*/7*p*/6*d*/5*f* characters (%) shown in parentheses. Values are from α MOs only due to substantial orbital mixing in Np and Pu complexes between the unpaired An electrons and ligand-dominant orbitals.

	$[Np^{3+}(NPC)_4]^-$	**2-Pu**	$[Np^{4+}(NPC)_4]$	**1-Pu**	$[Np^{5+}(NPC)_4]^+$	$[\mathbf{1\text{-}Pu}]^{M+}$
HOMO	97.7<br>(15.0/0.0/0.2/84.7)	80.7<br>(0.0/0.0/0.0/100.0)	58.2<br>(0.3/0.0/4.6/95.2)	6.3<br>(0.0/22.2/25.4/50.8)	5.0<br>(0.0/22.0/22.0/56.0)	3.3<br>(0.0/39.4/30.3/30.3)
HOMO-1	94.3<br>(0.0/0.0/2.8/97.2)	96.7<br>(0.0/0.0/2.6/97.3)	90.6<br>(14.1/0.0/0.7/85.3)	14.5<br>(0.0/4.1/13.8/81.4)	12.1<br>(0.0/10.7/15.7/73.6)	6.1<br>(4.9/31.1/26.2/39.3)
HOMO-2	95.5<br>(0.0/0.0/2.7/97.2)	92.8<br>(2.3/0.0/2.3/95.4)	67.6<br>(0.0/0.3/7.1/92.8)	17.2<br>(0.0/7.0/18.0/75.0)	16.0<br>(0.0/12.5/10.0/77.5)	6.0<br>(0.0/53.3/18.3/26.7)
HOMO-3	96.4<br>(0.9/0.0/2.4/96.6)	96.8<br>(8.0/0.0/0.8/91.2)	7.3<br>(0.0/13.7/4.1/82.2)	13.3<br>(1.5/5.3/6.8/86.5)	20.0<br>(0.0/3.5/5.5/91.0)	14.3<br>(8.4/1.4/7.7/81.8)
HOMO-4	4.4<br>(0.0/0.0/6.8/90.9)	93.6<br>(1.8/0.0/2.7/95.5)	10.9<br>(0.0/5.5/3.7/90.8)	13.2<br>(0.0/5.3/1.5/93.2)	16.6<br>(0.0/3.6/6.0/89.8)	6.7<br>(3.0/1.5/4.5/91.0)
HOMO-5	4.9<br>(0.0/4.1/10.2/83.7)	6.4<br>(0.0/0.0/4.7/95.3)	15.8<br>(0.0/16.5/2.5/80.4)	14.3<br>(0.7/7.0/12.6/79.0)	18.5<br>(0.0/1.6/9.7/89.2)	1.6<br>(0.0/0.0/18.8/75.0)
HOMO-6	3.7<br>(0.0/27.0/16.2/56.8)	6.2<br>(0.0/3.2/8.1/88.7)	8.9<br>(0.0/16.9/11.2/69.7)	24.0<br>(17.1/0.0/12.1/70.4)	2.9<br>(0.0/3.4/6.9/89.7)	2.7<br>(0.0/11.1/11.1/77.8)
HOMO-7	4.5<br>(0.0/37.8/17.8/42.2)	4.5<br>(0.0/28.9/17.8/53.3)	11.7<br>(0.0/17.1/6.8/75.2)	0.6<br>(0.0/0.0/0.0/83.3)	5.4<br>(0.0/11.1/11.1/77.8)	3.0<br>(0.0/6.7/26.7/63.3)
HOMO-8	4.8<br>(0.0/20.8/12.5/66.7)	5.7<br>(0.0/26.3/14.0/57.9)	23.4<br>(0.0/0.4/7.3/92.3)	8.0<br>(7.5/0.0/12.5/78.8)	8.1<br>(0.0/12.3/3.7/82.7)	11.2<br>(2.7/3.6/12.5/81.3)
HOMO-9	4.6<br>(0.0/43.5/30.4/26.1)	11.9<br>(0.0/4.2/2.5/93.3)	1.7<br>(0.0/5.9/11.8/82.4)	1.7<br>(17.6/0.0/5.9/76.5)	8.3<br>(0.0/4.8/8.4/86.7)	18.9<br>(5.3/0.0/18.5/76.2)
HOMO-10	7.6<br>(0.0/1.3/94.7/3.9)	8.0<br>(0.0/21.3/12.5/66.3)	2.4<br>(0.0/4.2/16.7/79.2)	5.2<br>(0.0/0.0/15.4/84.6)	34.1<br>(0.0/0.6/12.0/87.4)	27.1<br>(4.4/1.5/5.2/88.9)
HOMO-11	8.9<br>(0.0/3.4/93.3/3.4)	7.9<br>(0.0/1.3/86.1/12.7)	1.1<br>(0.0/9.1/27.3/63.6)	51.0<br>(5.5/0.4/6.7/87.5)	21.7<br>(0.0/1.8/13.4/84.8)	31.6<br>(1.9/0.3/15.8/82.0)
HOMO-12	0.1<br>(0.0/0.0/100.0/0.0)	8.9<br>(0.0/2.2/92.1/5.6)	2.4<br>(0.0/8.3/0.0/87.5)	66.9<br>(0.4/0.4/6.0/93.1)	14.6<br>(0.0/5.5/22.6/71.2)	24.0<br>(5.0/2.1/13.3/80.0)
HOMO-13	0.2<br>(0.0/0.0/50.0/0.0)	0.1<br>(0.0/0.0/100.0/0.0)	11.5<br>(0.0/1.7/60.0/38.3)	42.0<br>(10.2/0.0/16.9/72.6)	23.0<br>(0.0/0.0/20.4/79.1)	45.6<br>(14.0/0.0/9.6/76.1)
HOMO-14	0.2<br>(0.0/0.0/50.0/0.0)	0.3<br>(0.0/0.0/0.0/66.7)	16.1<br>(0.0/1.9/55.9/41.6)	54.4<br>(0.2/2.4/4.0/93.6)	22.9<br>(0.0/0.9/31.4/67.7)	30.9<br>(6.5/0.3/19.1/74.1)
HOMO-15	0.3<br>(0.0/0.0/33.3/33.3)	0.2<br>(0.0/0.0/0.0/50.0)	3.2<br>(0.0/0.0/43.8/56.3)	42.4<br>(0.9/1.4/15.1/82.5)	22.4<br>(0.4/3.6/15.6/80.4)	15.0<br>(0.0/2.0/52.0/46.0)
HOMO-16	2.5<br>(0.0/8.0/64.0/28.0)	0.4<br>(0.0/0.0/25.0/50.0)	6.5<br>(0.0/9.2/33.8/55.4)	23.3<br>(0.4/0.0/2.6/96.6)	21.2<br>(0.9/2.4/23.1/74.1)	28.5<br>(4.9/0.0/18.9/76.1)
HOMO-17	3.1<br>(0.0/6.5/64.5/29.0)	3.2<br>(0.0/3.1/46.9/50.0)	12.1<br>(0.0/4.1/15.7/79.3)	17.9<br>(0.0/3.4/4.5/91.6)	24.6<br>(0.0/2.0/18.7/78.9)	67.6<br>(0.0/1.8/3.6/94.7)
HOMO-18	4.2<br>(0.0/7.1/64.3/26.2)	4.0<br>(0.0/5.0/50.0/47.5)	7.6<br>(1.3/5.3/56.6/38.2)	19.6<br>(0.0/2.6/10.7/86.7)	3.6<br>(2.8/11.1/36.1/50.0)	66.1<br>(0.0/1.2/3.5/95.3)
HOMO-19	3.5<br>(31.4/0.0/11.4/57.1)	4.5<br>(0.0/4.4/57.8/35.6)	6.2<br>(3.2/16.1/48.4/32.3)	16.7<br>(0.6/3.6/18.6/77.2)	1.8<br>(0.0/5.6/55.6/38.9)	4.3<br>(0.0/7.0/23.3/72.1)

Table S12. Top 20 occupied orbitals of $[An^{n+}(NPC)_3(HNPC)]$ with the contributions of the An AOs (%) and 7*s*/7*p*/6*d*/5*f* characters (%) shown in parentheses. Values are from α MOs only due to substantial orbital mixing in Np and Pu complexes between the unpaired An electrons and ligand-dominant orbitals.

	$[Np^{4+}(NPC)_3(HNPC)]^+$	**3-Pu**
HOMO	82.8<br>(13.9/0.4/1.8/84.1)	9.3<br>(6.5/10.8/14.0/69.9)
HOMO-1	38.7<br>(0.5/2.3/2.3/94.6)	14.7<br>(0.0/6.1/18.4/74.8)
HOMO-2	44.8<br>(0.0/2.5/4.0/93.3)	16.3<br>(4.3/8.6/9.8/77.9)
HOMO-3	19.8<br>(0.0/7.1/6.1/86.9)	10.5<br>(7.6/6.7/9.5/76.2)
HOMO-4	12.8<br>(0.8/5.5/13.3/79.7)	12.5<br>(6.4/0.0/15.2/77.6)
HOMO-5	32.9<br>(0.6/0.0/4.3/94.8)	4.7<br>(2.1/4.3/21.3/70.2)
HOMO-6	43.9<br>(0.0/0.0/9.1/90.9)	4.9<br>(12.2/0.0/36.7/51.0)
HOMO-7	2.8<br>(0.0/7.1/21.4/71.4)	0.8<br>(0.0/12.5/0.0/87.5)
HOMO-8	8.2<br>(2.4/13.4/35.4/47.6)	33.8<br>(5.0/0.0/26.0/68.9)
HOMO-9	0.9<br>(0.0/0.0/33.3/66.7)	65.8<br>(4.4/0.8/6.5/88.4)
HOMO-10	21.4<br>(0.0/0.0/10.3/89.3)	50.4<br>(0.6/0.8/7.7/90.9)
HOMO-11	13.3<br>(0.0/1.5/68.4/29.3)	16.6<br>(7.2/1.8/7.8/83.1)
HOMO-12	5.7<br>(0.0/10.5/24.6/64.9)	30.9<br>(3.9/1.3/13.9/80.9)
HOMO-13	9.3<br>(0.0/2.2/20.4/77.4)	51.4<br>(0.2/0.2/1.6/98.2)
HOMO-14	0.9<br>(0.0/0.0/11.1/77.8)	28.1<br>(2.8/0.0/6.8/90.4)
HOMO-15	7.6<br>(0.0/3.9/50.0/46.1)	39.4<br>(2.3/0.8/0.8/96.4)
HOMO-16	7.0<br>(0.0/8.6/38.6/52.9)	34.5<br>(0.9/1.2/1.2/96.8)
HOMO-17	4.0<br>(5.0/10.0/65.0/20.0)	18.3<br>(0.5/3.8/13.7/81.4)
HOMO-18	5.3<br>(1.9/13.2/41.5/43.4)	7.0<br>(0.0/5.7/45.7/48.6)
HOMO-19	2.6<br>(0.0/7.7/50.0/42.3)	4.1<br>(0.0/12.2/34.1/56.1)

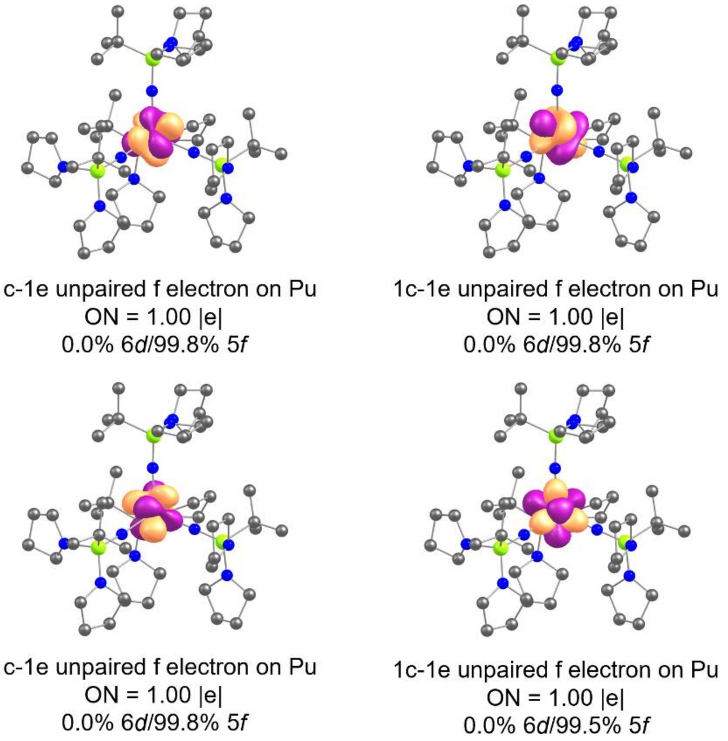

Figure S50. AdNDP results for **1-Pu**, showing four one-center one-electron elements (unpaired electrons) on Pu. C atoms are colored grey, N atoms are dark blue, P atoms are light green, and Pu is green, here and elsewhere in the SI. H atoms bonded to C are omitted for clarity, here and elsewhere in the SI.

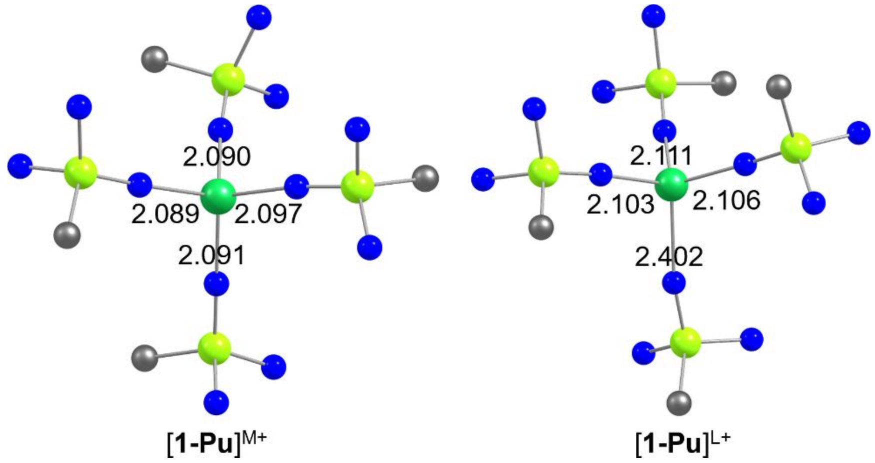

Figure S51. Optimized structures of complexes arising from metal-based oxidation ([**1-Pu**]$^{M+}$) or ligand-based oxidation ([**1-Pu**]$^{L+}$) of **1-Pu**. Pu–N bond lengths (Å) are shown for each Pu–N interaction.

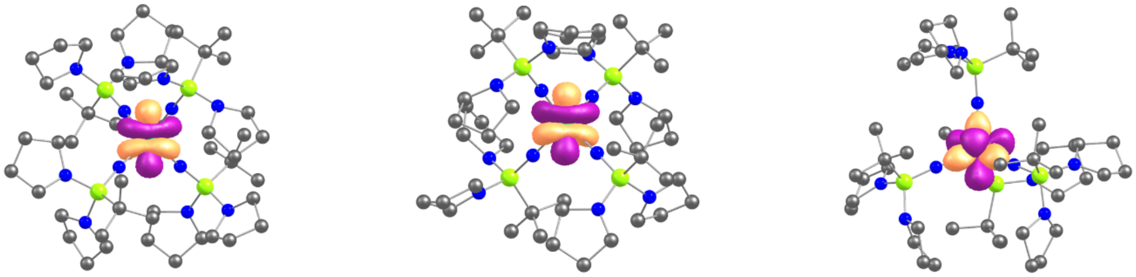

1c-1e unpaired f electron on Pu
ON = 1.00 |e|
0.0% 6*d*/99.6% 5*f*

1c-1e unpaired f electron on Pu
ON = 1.00 |e|
0.0% 6*d*/99.6% 5*f*

1c-1e unpaired f electron on Pu
ON = 0.99 |e|
0.0% 6*d*/99.7% 5*f*

Figure S52. AdNDP results for [**1-Pu**]$^{M+}$, showing three one-center one-electron elements (unpaired electrons) on Pu.

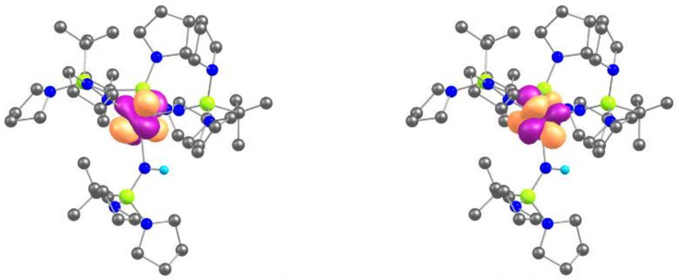

1c-1e unpaired f electron on Pu
ON = 1.00 |e|
0.0% 6*d*/99.7% 5*f*

1c-1e unpaired f electron on Pu
ON = 1.00 |e|
0.1% 6*d*/99.8% 5*f*

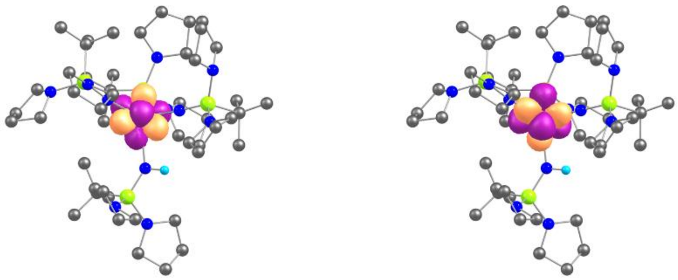

1c-1e unpaired f electron on Pu
ON = 1.00 |e|
0.0% 6*d*/99.8% 5*f*

1c-1e unpaired f electron on Pu
ON = 1.00 |e|
0.0% 6*d*/99.6% 5*f*

Figure S53. AdNDP results for **3-Pu**, showing four one-center one-electron elements (unpaired electrons) on Pu.

Table S13. QTAIM unpaired spin density on actinide (An) and N_{im} atoms ($\sum N_{im}$) in $[An^{n+}(NPC)_4]$. N^{ox}_{im} denotes the oxidized ligand.

QTAIM	$[Np^{3+}(NPC)_4]^-$	**2-Pu**	$[Np^{4+}(NPC)_4]$	**1-Pu**	$[Np^{5+}(NPC)_4]^+$	[**1-Pu**]$^{M+}$	[**1-Pu**]$^{L+}$
An	3.94	4.98	3.06	4.19	2.31	3.75	4.24
$\sum N_{im}$	0.02	0.00	-0.06	-0.16	-0.27	-0.65	-0.18
N^{ox}_{im}	-	-	-	-	-	-	0.79

Table S14. QTAIM unpaired spin density on actinide (An) and N_{im} atoms ($\sum N_{im}$) in $[An^{n+}(NPC)_3(HNPC)]$. $\sum N_{im}$ is the sum of spin density on the three non-protonated N_{im} atoms. N^*_{im} denotes the protonated ligand.

QTAIM	$[Np^{4+}(NPC)_3(HNPC)]^+$	**3-Pu**
An	3.09	4.24
$\sum N_{im}$	-0.09	-0.21
N^*_{im}	0.00	0.00

Table S15. NPA unpaired spin density on actinide (An) and N_{im} atoms ($\sum N_{im}$) in $[An^{n+}(NPC)_4]$. N^{ox}_{im} denotes the oxidized ligand.

NPA	$[Np^{3+}(NPC)_4]^-$	**2-Pu**	$Np^{4+}(NPC)_4$	**1-Pu**	$[Np^{5+}(NPC)_4]^+$	[**1-Pu**]$^{M+}$	[**1-Pu**]$^{L+}$
An	3.99	5.01	3.08	4.20	2.36	3.81	4.25
$\sum N_{im}$	-0.02	-0.03	-0.07	-0.17	-0.32	-0.73	-0.20
N^{ox}_{im}	-	-	-	-	-	-	0.82

Table S16. NPA unpaired spin density on actinide (An) and N_{im} atoms ($\sum N_{im}$) in $[An^{n+}(HNPC)(NPC)_3]$. $\sum N_{im}$ is the sum of spin density on the three non-protonated N_{im} atoms. N^*_{im} denotes the protonated ligand.

NPA	$[Np^{4+}(NPC)_3(HNPC)]^+$	**3-Pu**
An	3.11	4.26
$\sum N_{im}$	-0.10	-0.23
N^*_{im}	0.00	0.00

Table S17. Calculated $E_{1/2}$ (V *vs*. $Fc^{+/0}$) comparing the metal-based ($E_{1/2}$ M) and ligand-based ($E_{1/2}$ L) oxidations *vs*. the experimental values.

		$E_{1/2}$ M	$E_{1/2}$ L
	Exp.	Theor.	Theor.
[**1-Pu**]$^{+/0}$	-0.32	-0.16	+0.13
[**1-Pu**]$^{0/-}$	-2.83	-2.62	-0.67
$[Np(NPC)_4]^{+/0}$	-0.70	-0.75	+0.24

Table S18. Experimental and theoretical redox potentials (V *vs*. $Fc^{+/0}$).

	$E_{1/2}$ (V)		E_{pa} (V)				E_{pc} (V)			
	Exp.	Theor.	Exp.	VDE	ADE	ΔDE	Exp.	VEA	AEA	ΔEA
[**1-Pu**]$^{M+/0}$	-0.32	-0.16	-0.20	+0.28	-0.07	0.35	-0.44	-0.74	-0.10	0.64
[**3-Pu**]$^{0/-}$	-1.92	-1.79	-1.86	-1.15	-1.68	0.54	-1.98	-2.36	-1.73	0.63
[**1-Pu**]$^{0/-}$	-2.83	-2.62	-2.65	-1.89	-2.58	0.69	-3.01	-3.18	-2.63	0.55
$[Np(NPC)_4]^{M+/0}$	-0.70	-0.75	-0.62	-0.33	-0.75	0.42	-0.77	-1.24	-0.75	0.49
$[Np(NPC)_4]^{0/-}$	-	-3.23	-	-2.50	-3.15	0.65	-	-3.68	-3.22	0.46

Table S19. Calculated BDFE values (kcal/mol) including spin orbit coupling (SOC) corrections. DKH2 (without SOC correction) values are shown in brackets. Calculated pK_a using the Bordwell equation and experimental $E_{1/2}$ values.

	**BDFE**		**pK_a**	
	**Np**	**Pu**	**Np**	**Pu**
$[An^{4+}(NPC)_3(HNPC)]^+$	83.5 [83.0]	96.1 [95.1]	24.6 [24.2]	27.4 [26.6]

Table S20. QTAIM characteristics of the An–N bond critical point (BCP) and ρ at the N_{im} nuclear critical point (NCP) in the calculated structures of $[An^{n+}(NPC)_4]$.

Compound	ρ at An–N BCP (|e|/Bohr^3)	ρ at N_{im} NCP (|e|/Bohr^3)	$\nabla^2\rho$ at An–N BCP	H(r) at An–N BCP (Ha/Bohr^3)	δ(An,N)
[**1-Pu**]$^{M+}$	0.131	194.623	0.409	-0.050	1.19
$[Np^{5+}(NPC)_4]^+$	0.135	194.574	0.370	-0.056	1.18
**1-Pu**	0.112	194.551	0.349	-0.036	0.92
$[Np^{4+}(NPC)_4]$	0.114	194.531	0.328	-0.037	0.90
**2-Pu**	0.083	194.571	0.268	-0.018	0.65
$[Np^{3+}(NPC)_4]^-$	0.085	194.576	0.257	-0.019	0.67

Table S21. QTAIM characteristics of the An–N bond critical points in the calculated structures of [An^{n+}($(NPC)_3$(HNPC)]. Values are shown with the average of the three An–N interactions followed by the An–N* interaction.

	ρ (|e|/Bohr^3)	$\nabla^2\rho$	H(r) (Ha/Bohr^3)	δ(An,N)
**3-Pu**	0.127/0.063	0.380/0.202	-0.047/-0.009	1.08/0.45
[$Np^{4+}$$(NPC)_3$ (HNPC)]$^+$	0.128/0.063	0.358/0.203	-0.049/-0.009	1.05/0.46

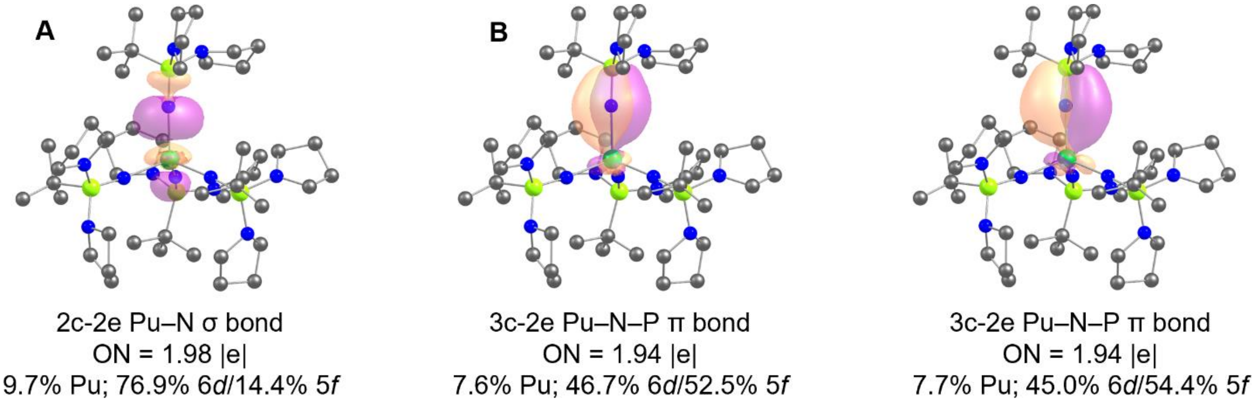

Figure S54. AdNDP elements describing Pu–N bonding orbitals for **1-Pu** showing (**A**) two-center two-electron Pu–N σ bond, and (**B**) two three-center two-electron Pu–N–P π bonds. An equivalent set of bonds is identified for the other three ligands. Qualitatively the same bonding pattern is found for all [$An^{n+}$$(NPC)_4$] complexes.

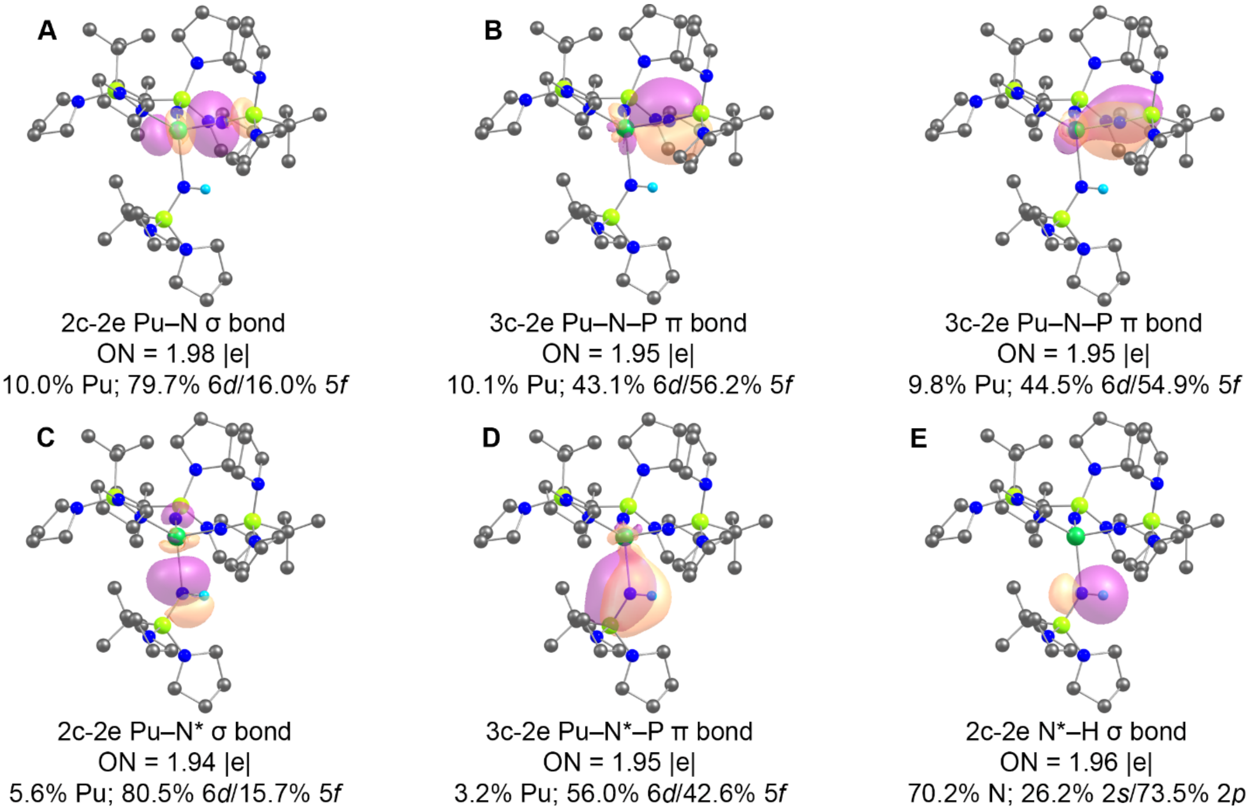

Figure S55. AdNDP Pu–N bonding orbitals for **3-Pu** showing (**A**) two-center two-electron Pu–N σ bond, (**B**) two three-center two-electron Pu–N–P π bonds, (**C**) two-center two-electron Pu–N* σ bond, (**D**) one three-center two-electron Pu–N*–P π bond, and (**E**) one two-center two-electron N*–H σ bond. N* denotes the protonated ligand. An equivalent set of bonds (**A**, **B**) is identified for the other two non-protonated ligands. The same bonding pattern is found for all $[An^{n+}(NPC)_3(HNPC)]$ complexes.

Table S22. Average population† (|e|) of the 6*d* and 5*f* orbitals of An in the σ and π An–N_{im}–(P) interactions for $[An^{n+}(NPC)_4]$.

	σ 6*d*	π 6*d*	6*d* Total‡	σ 5*f*	π 5*f*	5*f* Total‡	6*d*+5*f*
[**1-Pu**]$^{M+}$	0.174	0.081	0.337	0.054	0.182	0.418	0.754
$[Np^{5+}(NPC)_4]^+$	0.193	0.086	0.365	0.086	0.149	0.383	0.748
**1-Pu**	0.147	0.068	0.283	0.028	0.079	0.186	0.469
$Np^{4+}(NPC)_4$	0.152	0.070	0.291	0.038	0.069	0.175	0.465
**2-Pu**	0.098	0.050	0.198	0.013	0.016	0.044	0.242
$[Np^{3+}(NPC)_4]^-$	0.103	0.050	0.204	0.018	0.021	0.061	0.264

Table S23. Population† (|e|) of the 6*d* and 5*f* orbitals of An in the σ and π An–N_{im}–(P) interactions for [$An^{n+}$$(NPC)_3$(HNPC)]. Values are shown with the average of the three An–N followed by the An–NH.

	σ 6*d*	π 6*d*	6*d* Total‡	σ 5*f*	π 5*f*	5*f* Total‡	6*d*+5*f*
**3-Pu**	0.157/<br>0.088	0.085/<br>0.035	0.327/<br>0.123	0.032/<br>0.017	0.108/<br>0.026	0.247/<br>0.044	0.575/<br>0.167
[$Np^{4+}$$(NPC)_3$<br>(HNPC)]$^+$	0.163/<br>0.097	0.087/<br>0.039	0.338/<br>0.136	0.035/<br>0.034	0.099/<br>0.025	0.234/<br>0.060	0.572/<br>0.195

† The following formula was applied to calculate the orbital population: ON (|e|) of An–N_{im} bond × An–N_{im} bond polarization (An, %) × An (6*d*/5*f*) character (%)
‡ The total value stands for the σ + π + π(orthogonal) contributions.

References:

(1) Niklas, J. E.; Studvick, C. M.; Bacsa, J.; Popov, I. A.; La Pierre, H. S. Ligand Control of Oxidation and Crystallographic Disorder in the Isolation of Hexavalent Uranium Mono-Oxo Complexes. *Inorg. Chem.* **2023**, *62* (5), 2304-2316.

(2) Evans, W. J.; Kozimor, S. A.; Ziller, J. W.; Kaltsoyannis, N. Structure, Reactivity, and Density Functional Theory Analysis of the Six-Electron Reductant, $[(C_5Me_5)_2U]_2(\mu\text{-}\eta^6:\eta^6\text{-}C_6H_6)$, Synthesized via a New Mode of $(C_5Me_5)_3M$ Reactivity. *J. Am. Chem. Soc.* **2004**, *126* (44), 14533-14547.

(3) Otte, K. S.; Niklas, J. E.; Studvick, C. M.; Boggiano, A. C.; Bacsa, J.; Popov, I. A.; La Pierre, H. S. Divergent Stabilities of Tetravalent Cerium, Uranium, and Neptunium Imidophosphorane Complexes. *Angew. Chem., Int. Ed.* **2023**, *62* (62), e202306580.

(4) Cary, S. K.; Boland, K. S.; Cross, J. N.; Kozimor, S. A.; Scott, B. L. Advances in containment methods and plutonium recovery strategies that led to the structural characterization of plutonium(IV) tetrachloride tris-diphenylsulfoxide, $PuCl_4(OSPh_2)_3$. *Polyhedron* **2017**, *126*, 220-226.

(5) Goodwin, C. A. P.; Janicke, M. T.; Scott, B. L.; Gaunt, A. J. $[AnI_3(THF)_4]$ (An = Np, Pu) Preparation Bypassing An^0 Metal Precursors: Access to Np^{3+}/Pu^{3+} Nonaqueous and Organometallic Complexes. *J. Am. Chem. Soc.* **2021**, *143* (49), 20680-20696.

(6) Spinney, H. A.; Clough, C. R.; Cummins, C. C. The titanium tris-anilide cation [Ti(N[tBu]Ar)3]+ stabilized as its perfluoro-tetra-phenylborate salt: structural characterization and synthesis in connection with redox activity of 4,4′-bipyridine dititanium complexes. *Dalton Trans.* **2015**, *44* (15), 6784-6796, 10.1039/C5DT00105F.

(7) Jutzi, P.; Müller, C.; Stammler, A.; Stammler, H.-G. Synthesis, Crystal Structure, and Application of the Oxonium Acid $[H(OEt_2)_2]^+[B(C_6F_5)_4]$. *Organometallics* **2000**, *19* (7), 1442-1444.

(8) Cross, J. N.; Cary, S. K.; Stritzinger, J. T.; Polinski, M. J.; Albrecht Schmitt, T. E. Synthesis and Spectroscopy of New Plutonium(III) and -(IV) Molybdates: Comparisons of Electronic Characteristics. *Inorg. Chem.* **2014**, *53* (6), 3148-3152.

(9) Murillo, J.; Seed, J. A.; Wooles, A. J.; Oakley, M. S.; Goodwin, C. A. P.; Gregson, M.; Dan, D.; Chilton, N. F.; Gaunt, A. J.; Kozimor, S. A.; et al. Carbene Complexes of Plutonium: Structure, Bonding, and Divergent Reactivity to Lanthanide Analogs. *J. Am. Chem. Soc.* **2024**, *146* (6), 4098-4111.

(10) Otte, K. S.; Niklas, J. E.; Studvick, C. M.; Boggiano, A. C.; Bacsa, J.; Popov, I. A.; La Pierre, H. S. Divergent Stabilities of Tetravalent Cerium, Uranium, and Neptunium Imidophosphorane Complexes. *Angew. Chem. Int. Ed.* **2023**, e202306580.

(11) Savéant, J. M. *Elements of Molecular and Biomolecular Electrochemistry*; John Wiley & Sons, 2006. https://doi.org/10.1002/0471758078.ch1.

(12) Rountree, E. S.; Martin, D. J.; McCarthy, B. D.; Dempsey, J. L. Linear Free Energy Relationships in the Hydrogen Evolution Reaction: Kinetic Analysis of a Cobaloxime Catalyst. *ACS Catal.* **2016**, *6* (5), 3326-3335.

(13) Bruker. *SAINT-Plus*; Madison, Wisconsin, USA, 2016.

(14) Krause, L.; Herbst-Irmer, R.; Sheldrick, G. M.; Stalke, D. Comparison of silver and molybdenum microfocus X-ray sources for single-crystal structure determination. *J. Appl. Crystallogr.* **2015**, *48* (Pt 1), 3-10.

(15) Sheldrick, G. SHELXT – Integrated space-group and crystal-structure determination. *Acta Crystallogr. A* **2015**, *71*, 3-8.

(16) Dolomanov, O.; Bourhis, L.; Gildea, R.; Howard, J.; Puschmann, H. OLEX2: A complete structure solution, refinement and analysis program. *J. Appl. Cryst. J. Appl. Cryst.* **2009**, *42*, 339-341.

(17) Niklas, J. E.; Otte, K. S.; Studvick, C. M.; Roy Chowdhury, S.; Vlaisavljevich, B.; Bacsa, J.; Kleemiss, F.; Popov, I. A.; La Pierre, H. S. A tetrahedral neptunium(V) complex. *Nat. Chem.* **2024**, https://doi.org/10.1038/s41557-41024-01529-41556.

(18) Perdew, J. P.; Ernzerhof, M.; Burke, K. Rationale for mixing exact exchange with density functional approximations. *J. Chem. Phys.* **1996**, *105* (22), 9982-9985.

(19) Frisch, M. J.; Trucks, G. W.; Schlegel, H. B.; Scuseria, G. E.; Robb, M. A.; Cheeseman, J. R.; Scalmani, G.; Barone, V.; Petersson, G. A.; Nakatsuji, H.; et al. Gaussian 16, Revision C.01. *Gaussian Inc.* **2016**.
(20) Cao, X.; Dolg, M. Segmented contraction scheme for small-core actinide pseudopotential basis sets. *J. Molec. Struct.* **2004**, *673* (1), 203-209.
(21) Cao, X.; Dolg, M.; Stoll, H. Valence basis sets for relativistic energy-consistent small-core actinide pseudopotentials. *J. Chem. Phys.* **2003**, *118* (2), 487-496.
(22) Krishnan, R.; Binkley, J. S.; Seeger, R.; Pople, J. A. Self-consistent molecular orbital methods. XX. A basis set for correlated wave functions. *J. Chem. Phys.* **1980**, *72* (1), 650-654.
(23) Bruch, Q. J.; Connor, G. P.; Chen, C.-H.; Holland, P. L.; Mayer, J. M.; Hasanayn, F.; Miller, A. J. M. Dinitrogen Reduction to Ammonium at Rhenium Utilizing Light and Proton-Coupled Electron Transfer. *J. Am. Chem. Soc.* **2019**, *141* (51), 20198-20208.
(24) Dirac, P. A. M.; Fowler, R. H. The quantum theory of the electron. *Proc. R. Soc. London. Ser. A, Contain. Pap. a Math. Phys. Character* **1928**, *117* (778), 610-624.
(25) Reiher, M.; Wolf, A. Exact decoupling of the Dirac Hamiltonian. I. General theory. *J. Chem. Phys.* **2004**, *121* (5), 2037-2047.
(26) Douglas, M.; Kroll, N. M. Quantum electrodynamical corrections to the fine structure of helium. *Ann. Phys.* **1974**, *82* (1), 89-155.
(27) Hess, B. A. Applicability of the no-pair equation with free-particle projection operators to atomic and molecular structure calculations. *Phys. Rev. A* **1985**, *32* (2), 756-763.
(28) Hess, B. A. Relativistic electronic-structure calculations employing a two-component no-pair formalism with external-field projection operators. *Phys. Rev. A* **1986**, *33* (6), 3742-3748.
(29) Tomeček, J.; Liddle, S. T.; Kaltsoyannis, N. Actinide-Actinide Bonding: Electron Delocalisation and σ-Aromaticity in the Tri-Thorium Cluster $[\{Th(\eta^8-C_8H_8)(\mu-Cl)_2\}_3K_2]$. *Chem. Phys. Chem.* **2023**, *24* (18), e202300366.
(30) Szczepanik, D. W. The curious case of the crystalline tri-thorium cluster: cyclic delocalization without aromatic stabilization? *RSC Advances* **2023**, *13* (48), 34224-34229, 10.1039/D3RA06603G.
(31) Huang, Q.-R.; Kingham, J. R.; Kaltsoyannis, N. The strength of actinide–element bonds from the quantum theory of atoms-in-molecules. *Dalton Trans.* **2015**, *44* (6), 2554-2566, 10.1039/C4DT02323D.
(32) Jorge, F. E.; Canal Neto, A.; Camiletti, G. G.; Machado, S. F. Contracted Gaussian basis sets for Douglas–Kroll–Hess calculations: Estimating scalar relativistic effects of some atomic and molecular properties. *J. Chem. Phys.* **2009**, *130* (6).
(33) de Oliveira, A. Z.; Campos, C. T.; Jorge, F. E.; Ferreira, I. B.; Fantin, P. A. All-electron triple zeta basis sets for the actinides. *Comput. Theor. Chem.* **2018**, *1135*, 28-33.
(34) Zubarev, D. Y.; Boldyrev, A. I. Developing paradigms of chemical bonding: adaptive natural density partitioning. *Phys. Chem. Chem. Phys.* **2008**, *10* (34), 5207-5217, 10.1039/B804083D.
(35) Lu, T.; Chen, F. Multiwfn: A multifunctional wavefunction analyzer. *J. Comp. Chem.* **2012**, *33* (5), 580-592.
(36) Foster, J. P.; Weinhold, F. Natural hybrid orbitals. *J. Am. Chem. Soc.* **1980**, *102* (24), 7211-7218.
(37) Weinhold, F.; Landis, C. R. *Valency and Bonding*; Cambridge University Press, 2003.
(38) Kelley, M. P.; Popov, I. A.; Jung, J.; Batista, E. R.; Yang, P. δ and φ back-donation in AnIV metallacycles. *Nat. Commun.* **2020**, *11* (1), 1558.
(39) Kulichenko, M.; Fedik, N.; Boldyrev, A.; Muñoz-Castro, A. Expansion of Magnetic Aromaticity Criteria to Multilayer Structures: Magnetic Response and Spherical Aromaticity of Matryoshka-Like Cluster $[Sn@Cu_{12}@Sn_{20}]^{12-}$. *Chem. - Eur. J.* **2020**, *26* (10), 2263-2268.
(40) Sergeeva, A. P.; Boldyrev, A. I. The Chemical Bonding of Re_3Cl_9 and Revealed by the Adaptive Natural Density Partitioning Analyses. *Comments Inorg. Chem.* **2010**, *31* (1-2), 2-12.
(41) Bader, R. F. W. Atoms in molecules. *Acc. Chem. Res.* **1985**, *18* (1), 9-15.

(42) Bader, R. F. W. A Bond Path: A Universal Indicator of Bonded Interactions. *J. Phys. Chem. A* **1998**, *102* (37), 7314-7323.
(43) Matta, C. F.; Boyd, R. J. *The Quantum Theory of Atoms in Molecules*; Wiley-VCH, 2007.
(44) Cancès, E.; Mennucci, B. New applications of integral equations methods for solvation continuum models: ionic solutions and liquid crystals. *J. Math. Chem.* **1998**, *23* (3), 309-326.
(45) Cancès, E.; Mennucci, B.; Tomasi, J. A new integral equation formalism for the polarizable continuum model: Theoretical background and applications to isotropic and anisotropic dielectrics. *J. Chem. Phys.* **1997**, *107* (8), 3032-3041.
(46) Mennucci, B.; Cancès, E.; Tomasi, J. Evaluation of Solvent Effects in Isotropic and Anisotropic Dielectrics and in Ionic Solutions with a Unified Integral Equation Method: Theoretical Bases, Computational Implementation, and Numerical Applications. *J. Phys. Chem. B* **1997**, *101* (49), 10506-10517.
(47) Skripnikov, L. *Chemissian*; Skripnikov, L. : http://www.chemissian.com.
(48) Zhurko, G. A. *Chemcraft - Graphical Software for Visualization of Quantum Chemistry Computations*; 2005.
